# Accessing the Variability of Multicopy Genes in Complex Genomes using Unassembled Next-Generation Sequencing Reads: The Case of Trypanosoma cruzi Multigene Families

**DOI:** 10.1128/mbio.02319-22

**Published:** 2022-10-20

**Authors:** João Luís Reis-Cunha, Anderson Coqueiro-dos-Santos, Samuel Alexandre Pimenta-Carvalho, Larissa Pinheiro Marques, Gabriela F. Rodrigues-Luiz, Rodrigo P. Baptista, Laila Viana de Almeida, Nathan Ravi Medeiros Honorato, Francisco Pereira Lobo, Vanessa Gomes Fraga, Lucia Maria da Cunha Galvão, Lilian Lacerda Bueno, Ricardo Toshio Fujiwara, Mariana Santos Cardoso, Gustavo Coutinho Cerqueira, Daniella C. Bartholomeu

**Affiliations:** a Departamento de Parasitologia, Instituto de Ciências Biológicas, Universidade Federal de Minas Gerais, Belo Horizonte, Minas Gerais, Brazil; b Department of Biology, University of York, York, Yorkshire, United Kingdom; c Experimental Medicine Research Cluster (EMRC), University of Campinas (UNICAMP), Campinas, São Paulo, Brazil; d Center for Tropical and Emerging Global Diseases and Institute of Bioinformatics, The University of Georgia, Athens, Georgia, USA; e Houston Methodist Research Institute, Houston, Texas, USA; f Departamento de Genética e Evolução, Instituto de Ciências Biológicas, Universidade Federal de Minas Gerais, Belo Horizonte, Minas Gerais, Brazil; g Universidade Federal do Rio Grande do Norte, Centro de Ciências da Saúde, Programa de Pós-Graduação em Ciências Farmacêuticas, Natal, RN, Brasil; h Personal Genome Diagnostics, Baltimore, Maryland, USA; College of veterinary medicine, Cornell university

**Keywords:** multicopy genes, variability, copy number variation, complex genomes, *T. cruzi*, MASP, mucins, transsialidases, antigenicity

## Abstract

Repetitive elements cause assembly fragmentation in complex eukaryotic genomes, limiting the study of their variability. The genome of Trypanosoma cruzi, the parasite that causes Chagas disease, has a high repetitive content, including multigene families. Although many T. cruzi multigene families encode surface proteins that play pivotal roles in host-parasite interactions, their variability is currently underestimated, as their high repetitive content results in collapsed gene variants. To estimate sequence variability and copy number variation of multigene families, we developed a read-based approach that is independent of gene-specific read mapping and *de novo* assembly. This methodology was used to estimate the copy number and variability of MASP, TcMUC, and Trans-Sialidase (TS), the three largest T. cruzi multigene families, in 36 strains, including members of all six parasite discrete typing units (DTUs). We found that these three families present a specific pattern of variability and copy number among the distinct parasite DTUs. Inter-DTU hybrid strains presented a higher variability of these families, suggesting that maintaining a larger content of their members could be advantageous. In addition, in a chronic murine model and chronic Chagasic human patients, the immune response was focused on TS antigens, suggesting that targeting TS conserved sequences could be a potential avenue to improve diagnosis and vaccine design against Chagas disease. Finally, the proposed approach can be applied to study multicopy genes in any organism, opening new avenues to access sequence variability in complex genomes.

## INTRODUCTION

The low costs and increasing efficiency of sequencing technologies have enabled the assembling of genomes at an impressive rate (1, 2). However, despite many advances, the difficulties in assembling repetitive regions resulting in gene collapsing limit studies into the variability and evolution of these sequences (3). Many parasites’ genomes harbor large multigene families encoding surface antigens that play pivotal roles in host-parasite interactions (4, 5). Besides, most complex protozoan reference genomes and especially field isolates were not sequenced and assembled with methodologies that have enough resolution to generate both haplotypes from a diploid genome (6, 7). Therefore, only a mosaic haploid genome representation is usually available in public databases. Methodologies that explore the data directly from reads at high coverage can be an alternative to capture the complete genome variability, including collapsed repeats and sequence polymorphisms not incorporated into the assembled haploid genome representations.

The expansion of multigene families is especially remarkable in the genome of Trypanosoma cruzi, the etiological agent of Chagas disease, where multicopy genes encoding surface proteins, transposons, and other repeats encompass approximately 50% of the parasite genome (8), with evidence of collapsed regions (9). These T. cruzi multigene family genes are grouped in genomic clusters, which can span hundreds of kb and vary in size and content among the parasite DTUs (8, 10–12). These clusters are regions of loss of synteny not only between DTUs, but also between the haplotypes from the hybrid TcVI CL Brener strain (8, 10, 11), and account for 5.9 Mb of the genome size difference between CL Brener (TcVI) and the nonhybrid Sylvio (TcI) strain (13, 14). Multigene family content also varies within nonhybrid strains, such as Brazil (TcI) and Y (TcII) (15). The gene organization of these clusters is complex, where genes of each multigene family are not clustered together, but instead alternate in a nonorderly fashion, constantly altering the coding strand. It was speculated that this pattern of gene organization would avoid sequence homogenization through gene conversion (8, 16, 17). The largest T. cruzi multigene families are transsialidases (TS), mucin-associated surface proteins (MASPs), and mucins (TcMUCs), in which estimated gene copy numbers in each isolate can vary from 700 to 1,800 among families and DTUs (8, 11, 18). T. rangeli, a closely related parasite to T. cruzi that is nonpathogenic to the mammalian host, has a massive reduction in the gene counts of these multigene families (19), reinforcing their importance to mammalian host-parasite interaction processes. Although T. cruzi multigene families are crucial to host-parasite interplay (for a review see reference (20)), few studies compare their variability, which is usually performed at the level of assembled genomes, using a very limited number of strains (4, 8, 11, 13, 18, 19). Comparative studies of the repertoire of these families among DTUs are relevant because different groups have a specific pattern of geographical distribution, and not all DTUs are commonly found infecting humans (21–23). Accessing direct orthologs and estimating gene copy numbers in these families is complicated, not only due to genes collapsing during genome assembly but also due to the loss of synteny in these regions (8, 11, 12). Finally, assigning short reads to a specific gene in these large multigene repetitive families is challenging, as several conserved blocks are larger than the read length. Hence, the correct extent of their variability among T. cruzi DTUs and their biological impact is still poorly understood. To address these issues, we developed a read-based approach to estimate sequence variability and copy number variation of multigene-repetitive families that is independent of gene-specific read mapping and *de novo* assembly. Our approach was based on recovering reads that map to any member of each multigene family, followed by the estimation of variability, and copy number of k-mers and clusters generated by these reads. Our results showed large variability in multigene families, with DTU-specific patterns. We also employed this methodology to select representative sequences to assess the multigene families’ antigenicity, showing that these families were differentially targeted by the hosts’ immune response. This was the first work to compare the variability of multigene families from the six T. cruzi DTUs based on a large data set of sequencing reads and their antigenicity at a large scale. The proposed methodology can also be applied to assess the variability of multigene families or other repeats in any organism, once a reference genome was available.

## RESULTS

### Collapsed multicopy genes in long-reads T. cruzi genome assemblies.

To assess the potential collapsing of multigene family’s sequences in T. cruzi genomes assembled with long-reads, the coverage of each position in each chromosome of the DM28 (TcI), Ycl6 (TcII), and CL Brener (TcVI) was evaluated using Illumina reads from TcI, TcII and TcVI isolates. As seen in Fig. S1, there were several spikes of coverage in genomic regions containing clusters of multigene families, reinforcing the need for an alternative method to estimate genomic variability in these regions.

10.1128/mbio.02319-22.4 Fig S1 Evaluation of collapsing of multigene families’ sequences in different T. cruzi genome assemblies. In this image, three genome assemblies were evaluated. Dm28 (TcI) (first line) and Ycl6 (TcII) (second line) were assembled with a combination of long and short reads, while CL Brener Nonesmo (TcVI) (third line) was assembled with Sanger reads. The represented chromosomes/scaffolds were DM28, PRFA01000011; Ycl6, Chr3; CL-Brener Nonesmo, Chr23. The majority of the other chromosomes had a similar pattern (next slides). Three whole-genome sequence read libraries were assessed, a TcI (SRR3676317, first column), a TcII (SRR6357355, second column), and a TcVI (SRR6357354, third column) isolate. The blue line corresponds to the read depth of each position. Below, the protein-coding genes are depicted as rectangles drawn as proportional to their length, and their coding strand was indicated by their position above (top strand) or below (bottom strand) the central line. Colored boxes represent multigene families, where yellow, green, brown, orange, blue, and pink correspond to, respectively, Transsialidase, RHS, MASP, TcMUC, DGF-1, and GP63. Black and grey rectangles represent hypothetical and housekeeping genes, respectively. Gaps are represented by gene-less regions with no read coverage. Even in long-read assemblies, there was still a relevant increase in the read depth of multigene families, which reinforces the need for new methodologies to better access their variability. Download FIG S1, PDF file, 1.5 MB. Copyright © 2022 Reis-Cunha et al. 2022 Reis-Cunha et al. https://creativecommons.org/licenses/by/4.0/ This content is distributed under the terms of the Creative Commons Attribution 4.0 International license.

### T. cruzi isolates phylogeny and whole-genome variability.

**FIG 1:**
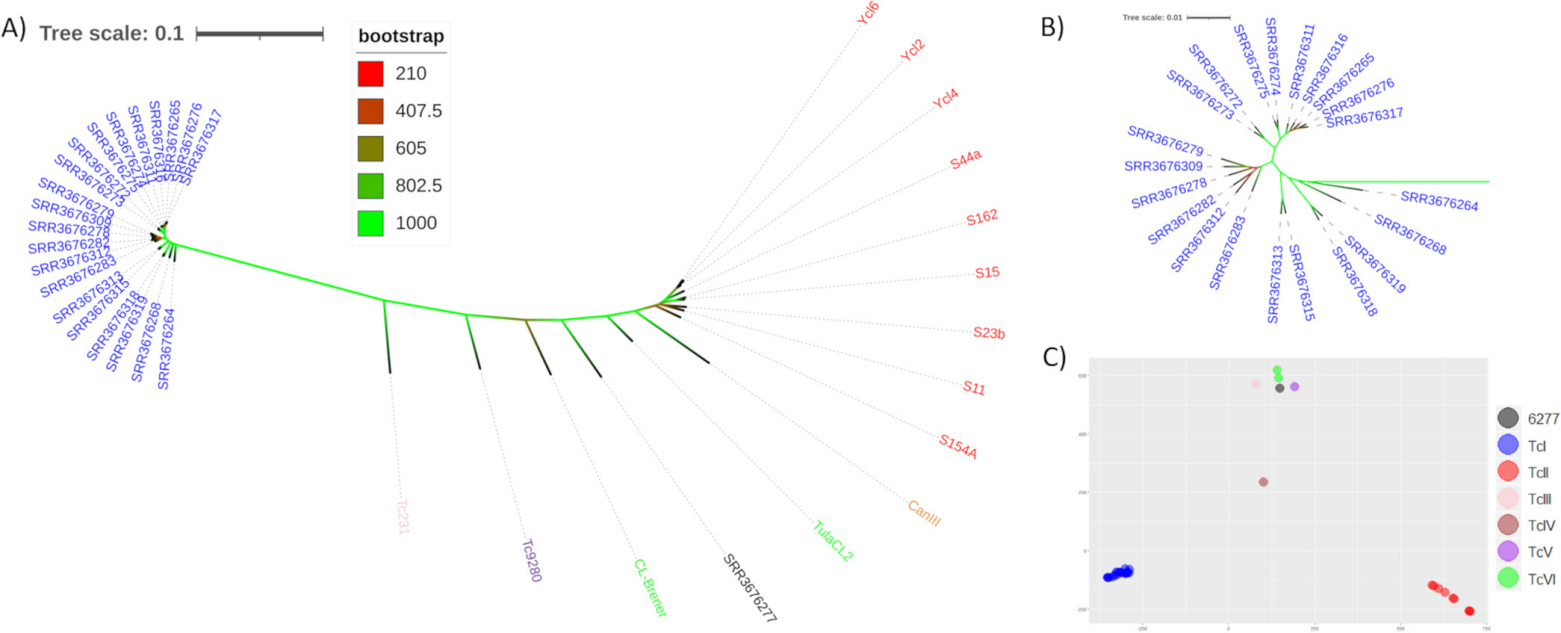
Phylogeny and whole-genome variation comparison of T. cruzi strains and isolates. (A) Unrooted maximum likelihood phylogenetic tree of the 36 T. cruzi strains based on 1,563 single copy genes (51), with 1000 bootstrap replicates. (B) Zoomed in on the TcI branch to ease visualization. (C) PCA based on SNPs of the 36 T. cruzi strains. In this image, the *x*-axis and *y*-axis represent 45.28% and 15.96% of the variability observed in the evaluated isolates, respectively. In both images, the T. cruzi DTUs TcI, TcII, TcIII, TcIV, TcV, and TcVI are represented, respectively, by the colors blue, red, pink, orange, purple, and green. The number 6277 corresponds to the sample SRR3676277.

To provide a broad evaluation of the T. cruzi multigene family content and variability, whole-genome sequence (WGS) libraries from 36 parasite isolates, including at least one representative of each DTU that infects humans (TcI-TcVI) were used (Table S1). Initially, to confirm the phylogenetic classification of the 36 T. cruzi isolates, the maximum likelihood (ML) phylogeny based on 1,563 single copy genes (totalizing 2,355,325 nucleotides) (Fig. 1A and B), as well as a principal component analysis (PCA) based on single nucleotide polymorphism (SNP) variations in the whole genomic sequence of these isolates, were estimated (Fig. 1C). Both analyses showed a clear separation between samples from the different DTUs. The T. cruzi isolate SRR3676277, which was described in SRA as a “TcI” strain, clustered with the TcVI strains in the ML phylogeny as well as in the PCA, which implies that it was also a hybrid strain. All downstream results obtained with this sample also support this assumption. Therefore, we reclassified SRR3676277 to the TcVI DTU.

10.1128/mbio.02319-22.2 Table S1 Description of the read libraries used in this work. Each line corresponds to a T. cruzi isolate. The columns “K-mer”, “Cluster” and “K-mer/Cluster” correspond, respectively, to the total number of different k-mers, and clusters, and mean the number of k-mer in each cluster for each T. cruzi strain. These counts were only based on presence/absence without accounting for the copy number of each k-mer and cluster. The column “Cluster sum” corresponds to the sum of coverage of each cluster for a given strain, which was proportional to the multigene family copy number in the genome. Download Table S1, XLSX file, 0.02 MB. Copyright © 2022 Reis-Cunha et al. 2022 Reis-Cunha et al. https://creativecommons.org/licenses/by/4.0/ This content is distributed under the terms of the Creative Commons Attribution 4.0 International license.

### T. cruzi multigene families k-mer generation and clusterization.

To generate representative k-mers for MASP, TcMUC and TS multigene families, each of the 36 WGS read libraries was mapped in a representative reference genome file as described in Materials and Methods. Reads which were mapped in any gene or pseudogene from MASP, TcMUC, or TS were recovered and used to generate 30 nt-long k-mers for each family. Redundancy and small variations among k-mers were removed by clustering based on sequence similarity (clustering parameter selection can be seen in Text S1). Table 1 summarizes the k-mers and cluster metrics. The clusterization step resulted in a higher shared proportion of sequences among T. cruzi isolates (~20 to 40%) compared with k-mers (0.27 to 0.60%). This shows that, although there were substantial differences in multigene families when all sequence variations were considered (k-mers), there were a significant number of conserved blocks (clusters) among DTUs (Table 1). TS was the most conserved family, presenting a mean of 81.52 k-mers/cluster, and ~40% of shared clusters in all evaluated strains (Table 1).

**TABLE 1:** Overall number of different k-mers and clusters in T. cruzi strains^*a*^

Gene family	K-mer Total^*b*^	Conserved^*c*^	Conserved (%)^*d*^	Cluster total	Conserved	Conserved (%)	K-mer/Cluster^*e*^
TcMUC	971,444	2,628	0.27	16,913	3,388	20.03	57.44
MASP	2,520,564	6,439	0.26	44,633	13,680	30.65	56.47
TS	4,500,268	26,913	0.60	55,204	24,050	43.57	81.50

a Variability of different k-mers and clusters in the 36 evaluated *T. cruzi* strains.

b Total: total number of different k-mers or clusters in all strains.

c Conserved: number of k-mer or clusters shared among all *T. cruzi* strains.

d Conserved (%): percentage of conserved k-mers or clusters.

e K-mer/Cluster: represents themean number of k-mers in each cluster.

10.1128/mbio.02319-22.1 Text S1 Detailed description of the methods and analysis used to support the conclusions of the manuscript. Download Text S1, DOCX file, 0.02 MB. Copyright © 2022 Reis-Cunha et al. 2022 Reis-Cunha et al. https://creativecommons.org/licenses/by/4.0/ This content is distributed under the terms of the Creative Commons Attribution 4.0 International license.

### T. cruzi multigene families’ clusters were variable among DTUs and correlated with phylogeny.

**FIG 2:**
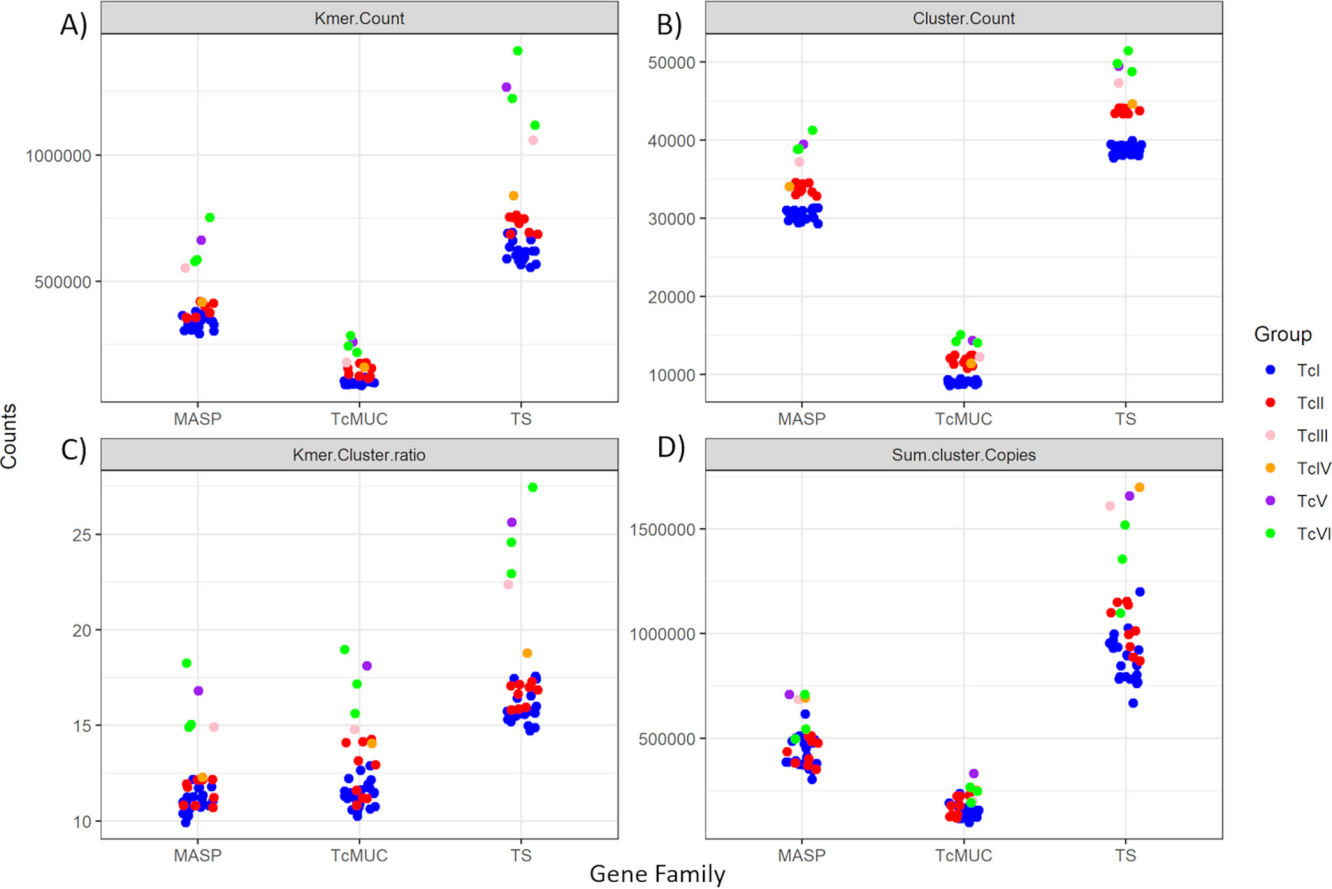
K-mers and clusters variability and copy number within each T. cruzi strain. Each dot corresponds to a T. cruzi isolate. (A) “K-mer.Count,” (B) “Cluster. Count” and (C) “K-mer.Cluster.ratio” correspond, respectively, to the total number of different k-mers, clusters, and mean a number of k-mer in each cluster for each T. cruzi strain. These counts were only based on presence/absence without accounting for the copy number of each k-mer and cluster. (D) “Sum.cluster.Copies” corresponds to the sum of coverage of each cluster for a given strain, which was proportional to the multigene family copy number in the genome. Strain-specific values can be seen in Table S2.

To estimate the multigene family’s variability within each parasite isolate, the 36 T. cruzi strains were compared based on four parameters: (i) number of different k-mers (Fig. 2A); (ii) number of clusters (Fig. 2B); (iii) mean number of k-mers per cluster (Fig. 2C); and (iv) sum of coverages of all clusters, which was proportional to the multigene family copy number in a given strain/isolate (Fig. 2D). We selected the sum of coverages of clusters in a family as a metric to represent its copy number, as it was proportional and more representative than the most conserved cluster, especially for pseudogenes in the TS family (Text S1). Because genes in each family have similar sizes in the evaluated reference genomes, the potential bias of using the sum of counts for comparing the copy number of each family among isolates was low (Text S1). TcI strains had an overall smaller number of different k-mers and clusters compared with TcII-TcVI (Fig. 2A and B). The overall copy number of the three multigene families was lower in TcI and TcII compared with TcIII-TcVI (Fig. 2D). The lower number of different clusters (Fig. 2B) but similar copy number (Fig. 2D) in TcI compared with TcII suggested an expansion of redundant sequences in TcI. The isolate SRR3676277 presented a cluster copy number and variability compared to the observed TcV-TcVI hybrid strains (Table S1), reinforcing its classification as a TcVI strain.

10.1128/mbio.02319-22.3 Table S2 Peptide array and immunoblotting of the 335 selected multigene family’s targets. In this table, the columns “Array Position”, “Peptide Sequence”, and “Multigene Family” correspond to the immunoblot membrane position, the sequence, and the multigene family of each one of the 335 selected peptides, respectively. The columns “CN Chronic”, “Colombiana Chronic”, “Y Chronic” and “CL Brener Chronic” correspond to the seroreactivity of mice in the chronic stage; while the columns “CN Acute”, “Colombiana Acute”, “Y Acute” and “CL Brener Acute” correspond to the seroreactivity of mice in the acute stage of T. cruzi infection. Download Table S2, XLSX file, 0.04 MB. Copyright © 2022 Reis-Cunha et al. 2022 Reis-Cunha et al. https://creativecommons.org/licenses/by/4.0/ This content is distributed under the terms of the Creative Commons Attribution 4.0 International license.

**FIG 3:**
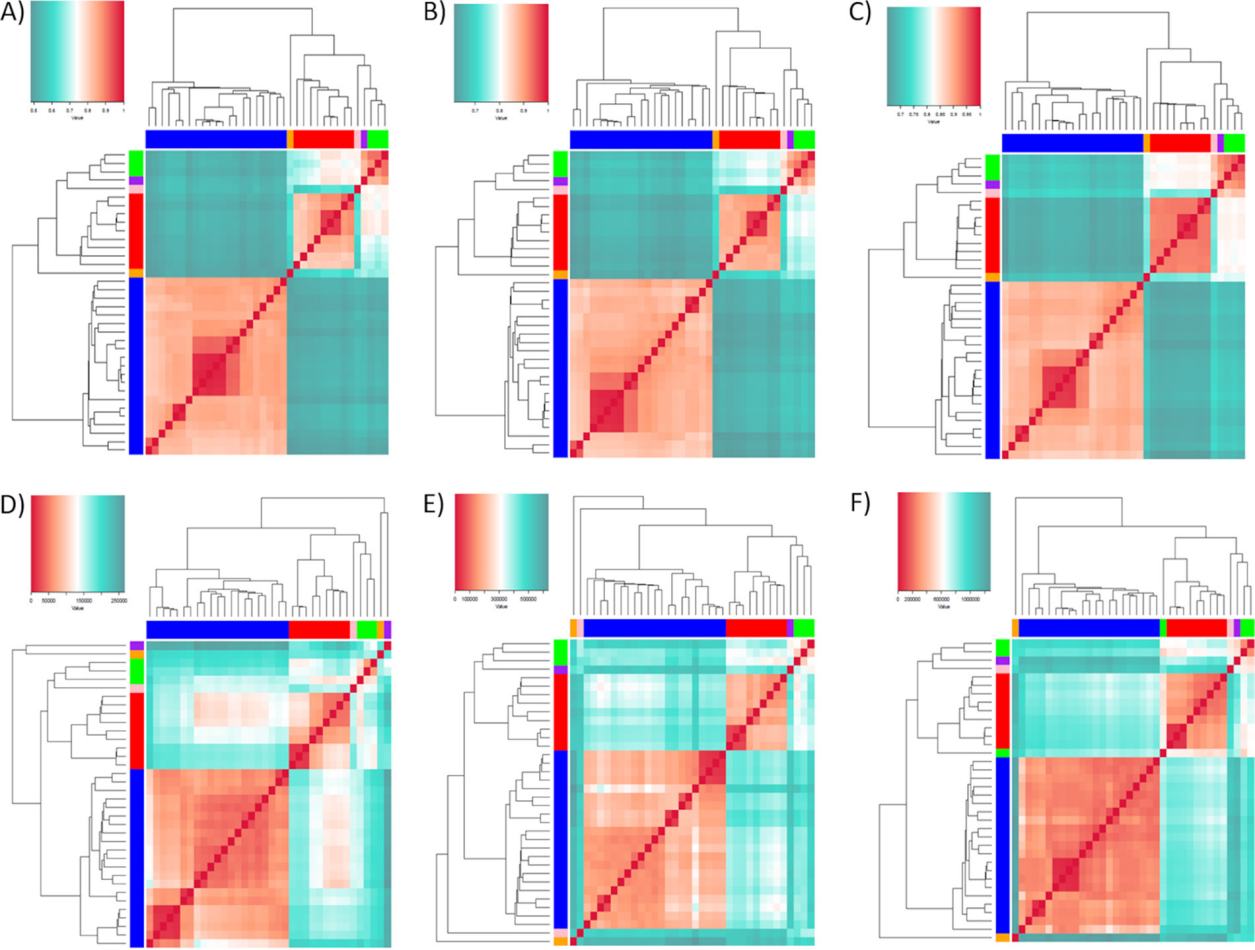
Heatmap of the cluster variability and copy number among T. cruzi isolates. Cluster variability was estimated by the Jaccard Coefficient (JC) based on the presence/absence of clusters for each multigene family: (A) TcMUC, (B) MASP, and (C) TS. JC values are represented on a scale from green (low) to white (medium) to red (high) similarity. Cluster copy number variability was estimated by Manhattan distance for each multigene family. (D) TcMUC, (E) MASP, and (F) TS. Manhattan distance values are represented in a scale from green (high), white (medium) to red (low) distances. In this image, each line and column correspond to a T. cruzi isolate. The DTU of each isolate was represented by colored lateral strips, where blue, red, pink, orange, purple and green correspond to, respectively, TcI, TcII, TcIII, TcIV, TcV, and TcVI. Lateral dendrograms were generated by UPGMA clustering. A larger version of each image with the names of each isolate is available in (Fig. S2).

The multigene family’s variability among strains was assessed based on two parameters: (i) the presence or absence of clusters (i.e., motifs) using the Jaccard coefficient (JC); and (ii) the motif copy number, using the Manhattan distance of the cluster copy numbers. When only the presence/absence of motifs was assessed, the isolates from TcI and TcII DTUs formed clear distinct groups (Fig. 3A to C), suggesting the occurrence of DTU-specific motifs. This could be caused by their long evolutive divergence and possible low occurrence of recombination between DTUs. Strains from hybrid DTUs, TcV, and TcVI, grouped with SRR3676277, and closer to TcIII and TcII strains, their parental DTUs, reinforcing their hybrid nature. In addition, the JC distance of multigene family cluster variability grouped TcV and TcVI strains closer to TcIII than to TcII, which suggests that a larger content of multigene family’s different clusters was shared between them and these parental DTUs. When the motif copy number was assessed, TcI and TcII strains also formed separated groups (Fig. 3D and Fig. E, Fig. S3). However, the clustering of TcVI hybrid strains was complex. CL Brener, Tulahuen, and SRR3676277 grouped closer to TcIII based on TcMUC cluster counts (Fig. 3D), to TcII for MASP (Fig. 3E); and both CL Brener and SRR3676277 grouped closer to TcIII and Tulahuen closer to TcII for TS (Fig. 3F). This could be a result of a differential resolution of the hybridization event by CL Brener/SRR3676277 and Tulahuen, or could also be caused by differences in chromosomal duplications, which vary between these strains. There was a high correspondence between the multigene families dendrograms and core-genome phylogeny, where most of the incongruences occurred in small branches within DTUs or the Tc231 (TcIII), CanIII (TcIV) and Tc9280 (TcV) strains (Fig. S4).

10.1128/mbio.02319-22.6 Fig S3 Dendrograms of the cluster CNV. Dendrograms with bootstrap support based on the cluster copy number of each strain were generated for the multigene families (A) TcMUC, (B) MASP, and (C) TS, with the R package Pvclust (https://cran.r-project.org/web/packages/pvclust/index.html), using Manhattan distance, “average” clustering and 1,000 bootstrap replicates. Values in green correspond to the bootstrap probability (BP), while values in red correspond to the approximately unbiased probability values (AU). Download FIG S3, PDF file, 0.3 MB. Copyright © 2022 Reis-Cunha et al. 2022 Reis-Cunha et al. https://creativecommons.org/licenses/by/4.0/ This content is distributed under the terms of the Creative Commons Attribution 4.0 International license.

10.1128/mbio.02319-22.7 Fig S4 Comparative tanglegrams of single copy core-genome phylogeny and multigene family’s clusters CNV. This image corresponds to comparative tanglegrams of (A) core-genome (CG) phylogeny and TcMUC clusters; (B) CG phylogeny and MASP clusters; (C) CG phylogeny and TS clusters; (D) MASP and TcMUC clusters; (E) MASP and TS clusters; and (F) TcMUC and TS clusters. The dendrograms were rooted in the Tc9280 clade, and the branches were flipped (but not replaced) to adjust to the linking between nodes. The DTU of origin of each sample is represented by colors, where blue, red, pink, orange, purple, green, and black correspond, respectively, to TcI, TcII, TcIII, TcIV, TcV, and TcVI. Download FIG S4, PDF file, 1.3 MB. Copyright © 2022 Reis-Cunha et al. 2022 Reis-Cunha et al. https://creativecommons.org/licenses/by/4.0/ This content is distributed under the terms of the Creative Commons Attribution 4.0 International license.

10.1128/mbio.02319-22.5 Fig S2 Large representation of Fig. 3, including the strain names. Download FIG S2, PDF file, 0.4 MB. Copyright © 2022 Reis-Cunha et al. 2022 Reis-Cunha et al. https://creativecommons.org/licenses/by/4.0/ This content is distributed under the terms of the Creative Commons Attribution 4.0 International license.

### T. cruzi multigene family’s variability correlated with genome size and hybrid nature.

**FIG 4:**
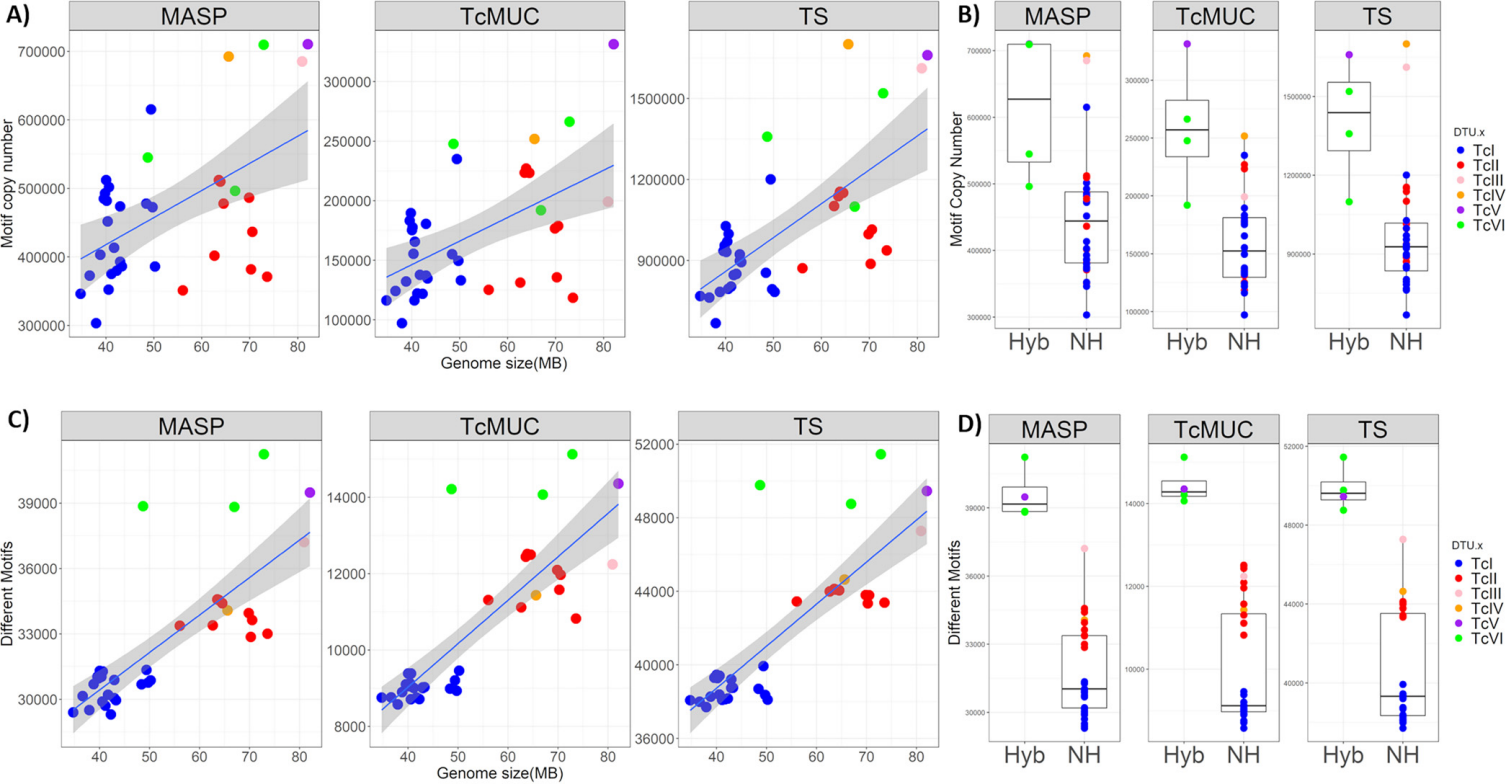
Correlation between cluster copy number, variability, and genome size in hybrid and nonhybrid DTUs. (A) Correlation between genome size and cluster copy number in the 36 T. cruzi strains. In this image, each dot corresponds to a T. cruzi strain, the *y*-axis corresponds to the sum of the copy number of all clusters in each strain and the *x*-axis corresponds to the genome size. The correlation between these two axes was estimated using Spearman’s rank order: MASP (rho = 0.393, *P* = 0.0183); TcMUC (rho = 0.469, *P* = 0.0042); TS (rho = 0.627, *P* = 6.14 × 10^−5^) (B) Boxplot of the cluster copy number in hybrid (Hyb) and nonhybrid (NH) DTUs. The statistical significance between the groups was estimated using the Mann-Whitney test: MASP (*P* = 3.19 × 10^−3^); TcMUC (*P* = 1.29 × 10^−3^); TS (*P* = 5.26 × 10^−3^). (C) Correlation between genome size and cluster variability in the 36 T. cruzi strains. In this image, each dot corresponds to a T. cruzi strain, the *y*-axis corresponds to the number of different clusters in each strain and the *x*-axis corresponds to the genome size. The correlation between these two axes was estimated using Spearman’s rank order: MASP (rho = 0.749, *P* = 7.562 × 10^−7^); TcMUC (rho = 0.778, *P* = 2.236 × 10^−8^); TS (rho = 0.752, *P* = 6,941 × 10^−7^). (D) Boxplot of the cluster variability in hybrid (Hyb) and nonhybrid (NHyb) DTUs. The statistical significance between the groups was estimated using the Mann-Whitney test: MASP (*P* = 3.39 × 10^−5^); TcMUC (*P* = 1.39 × 10^−3^); TS (*P* = 2.39 × 10^−5^).

To evaluate if there was a correlation between T. cruzi multigene family cluster variability and copy number and the parasite genome size, the number of different clusters and the sum of coverage of clusters were compared with the estimated genome size for all 36 T. cruzi strains (Fig. 4). There were moderate correlations between the multigene family’s cluster copy number and the genome size for MASP and TcMUC, and strong for TS (Fig. 4A), with a significant increase in the copy number of clusters in the hybrid compared to nonhybrid DTUs (Fig. 4B). The single isolates from TcIII and TcIV DTUs had a comparable cluster copy number with the hybrid strains for MASP and TS multigene families (Fig. 4A). More isolates from these DTUs were needed to evaluate if their copy numbers were consistently similar to the hybrid strains. Next, the correlation between multigene family’s cluster variability (number of different clusters with above zero coverage) in each isolate, was compared with the genome size (Fig. 4C). Strong positive correlation was observed for the three multigene families, suggesting that the increase in genome size results in an increased diversification of these multigene family sequences. Hybrid strains have a higher cluster variability than nonhybrid strains (Fig. 4D), reinforcing that the multigene family content from both parental strains was partially maintained after the hybridization event, resulting in an increased sequence variability. There was no strong correlation between somy variation and cluster copy numbers (Text S1). However, this lack of correlation may be caused by the differential expansion of multigene families in different chromosomes among strains.

### Antigenicity of T. cruzi multigene families.

**FIG 5:**
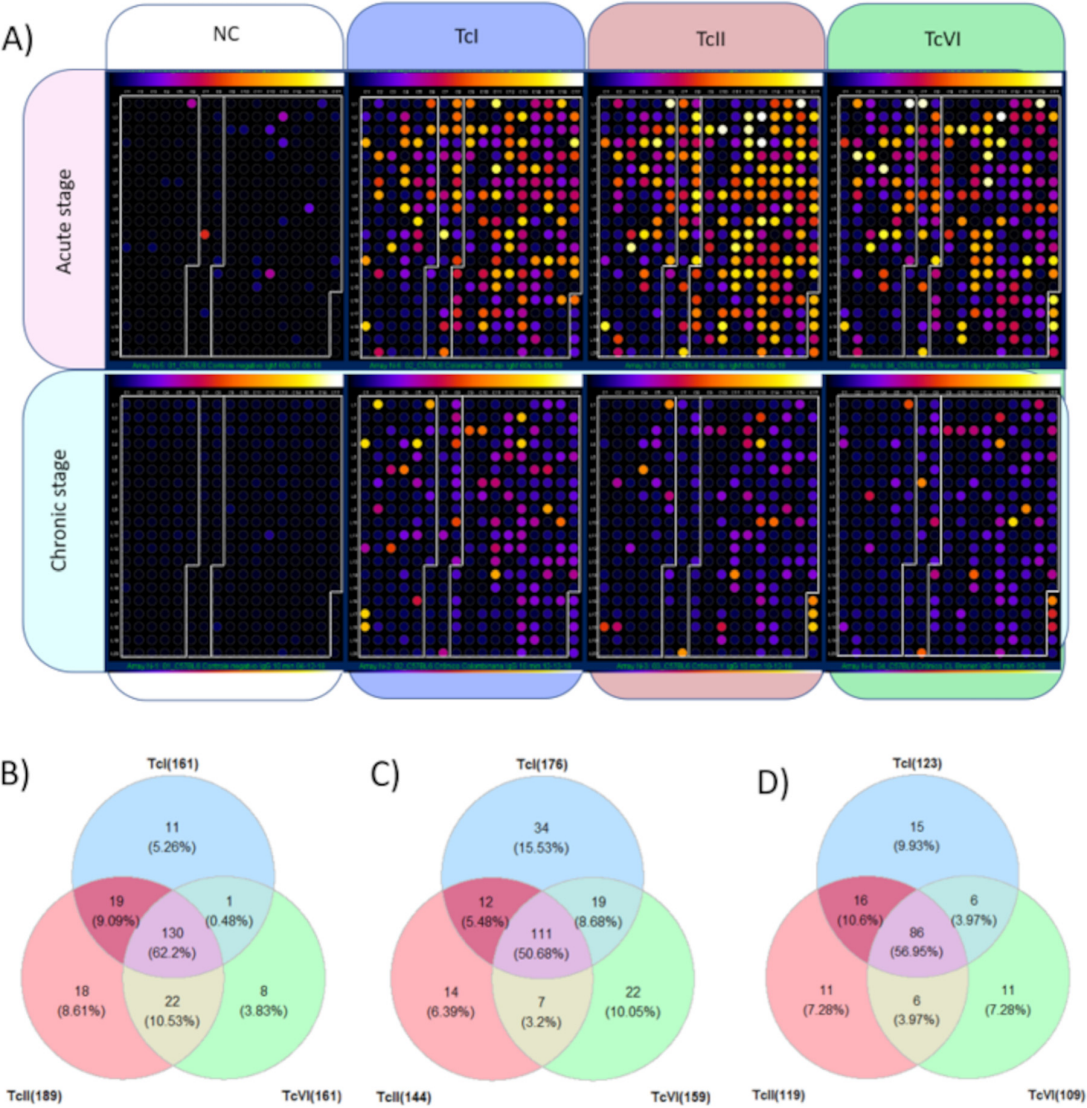
Antigenicity of peptides derived from the multigene families using sera of mice infected with different T. cruzi DTUs. (A) Each dot corresponds to a peptide, and the white boxes in each panel separate the peptides from the MASP (left), TcMUC (middle), and TS (right) multigene families. The reactivity of each peptide is represented on a scale from black (low reactivity), orange (median reactivity) to white (high reactivity). The panels representing the reactivity of the sera from mice in the acute phase were circumvented horizontally by a pink box, while the ones representing the sera from mice in the chronic phase are by a cyan box. The plots vertically circumvented by white, blue, salmon, and green boxes represent, respectively, the reactivity from the peptides to the sera of noninfected mice (NC), or mice infected with TcI, TcII, or TcVI strains. Venn diagrams representing the number of peptides with above cutoff reactivity for the pool of sera collected during the acute (B), chronic (C), or both acute and chronic (D) phases of infection. Percentage values correspond to the fraction of the reactive peptides that were observed in each quadrant.

**FIG 6:**
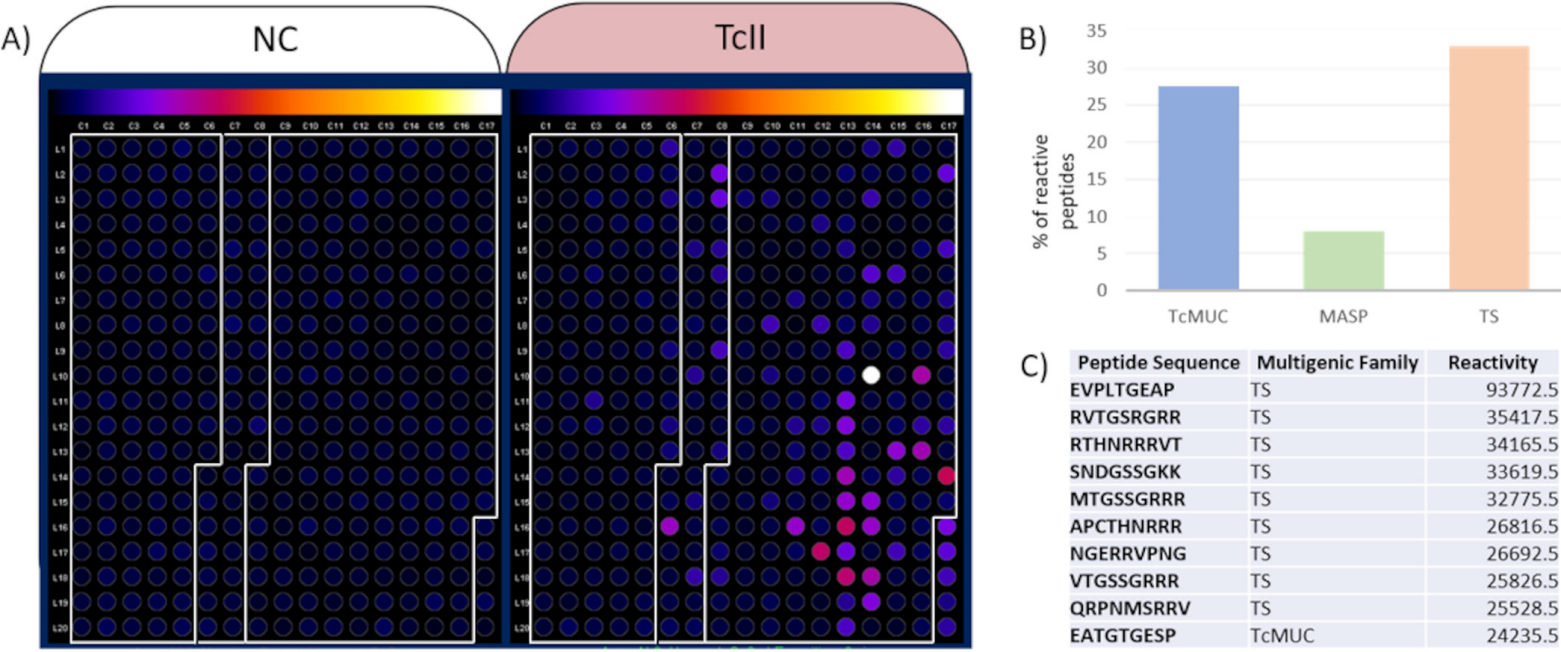
Antigenicity of peptides derived from the multigene families with sera of Chagasic human patients infected with TcII strains. (A) Each dot corresponds to a different peptide, where the white boxes in each panel separate the peptides from the MASP (left), TcMUC (middle), and TS (right). The reactivity of each peptide is represented on a scale from black (low reactivity), orange (median reactivity) to white (high reactivity). (B) Percentage of the peptides from each multigene family that presented reactivity above the cutoff. (C) Top 10 peptides with the highest human sera reactivity.

As surface proteins are one of the main interfaces between the parasite and the host, the observed MASP, TcMUC, and TS variability may impact the mammalian host's immune response to the infection. To assess if the observed variability impacts the antigenicity of these multigene families, a total of 335 representative peptides, comprising 40, 113, and 182 k-mers, respectively, for the multigene families TcMUC, MASP, and TS (Table 2 and Text S1), were screened by immunoblotting. Two sera panels were used: (i) sera from C57BL6 mice experimentally infected with TcI, TcII, and TcVI strains, collected during acute and chronic phases of T. cruzi infection to assess the variability in host recognition in the acute and early chronic stages of the infection (Fig. 5 and Table S2), and (ii) sera from chronic Chagasic patients to assess variability in host recognition in the long-lasting chronic human infection (Fig. 6).

**TABLE 2:** Multigene families representative peptides selection^*a*^

Gene family	Total K-mers	High copy	Conserved	Non-Pseudogene	B-cell epitope	Non-redundant	Im. score >0.42
TcMUC	971,444	2,628	1,928	1,456	600	59	40
MASP	2,520,564	6,439	3,434	2,443	600	150	113
TS	4,500,268	26,913	14,481	13,763	600	182	182

a The selection of representative peptides for the three multigene families was based on conservation, high copy number, and high B-cell epitope prediction score. Im score, immunogenicity score.

When the peptide array was screened with sera from mice infected with T. cruzi strains from the TcI, TcII, or TcVI DTUs (Fig. 5A), a total of 209 (62.38%) and 219 (65.37%) peptides were reactive to the sera pools, respectively, obtained in the acute or chronic stages of the infection. Of those, 151 (45.37%) were reactive with sera from both acute and chronic infection, suggesting that the recognition of these peptides was present throughout the infection. The distribution of reactive peptides among sera from TcI, TcII, and TcVI strains during acute, chronic, or both acute and chronic infections is depicted in Fig. 5B to D. The infection chronification was accompanied by a decrease in the number of peptides that were reactive to all strains (130 to 111), by an increase in the DTU specific reactive peptides for TcI (11 to 34) and TcVI (8 to 22), and by a discrete decrease in the number TcII-specific reactive peptides (18 to 14) (Fig. 5B and C). When the seroreactivity of each multigene family was evaluated separately, there was an increase in variability of the reactive peptides in different DTUs with chronification, which was more evident for the MASP family (Fig. S5). This shows that there was an expansion of DTU-specific reactivity with chronification in the mice model.

10.1128/mbio.02319-22.8 Fig S5 Venn diagram of the multigene family’s reactive peptides to the sera of mice in the acute and chronic phases of T. cruzi infection. A total of 40 TcMUC, 113 MASP, and 182 TS peptides were evaluated. The number in parenthesis below each DTU, ex. TcI (19), represents the number of peptides that were reactive with the sera of mice infected with TcI DTU, in the acute stage. “Acute”: results for the sera of mice in the acute stage. “Chronic”: result for the sera of mice in the chronic stage. “Acute and Chronic”: peptides that had above cutoff values in the acute and chronic stages simultaneously. Percentage values correspond to the fraction of the reactive peptides that were observed in each quadrant. Download FIG S5, PDF file, 0.4 MB. Copyright © 2022 Reis-Cunha et al. 2022 Reis-Cunha et al. https://creativecommons.org/licenses/by/4.0/ This content is distributed under the terms of the Creative Commons Attribution 4.0 International license.

Next, the seroreactivity of late-chronic Chagasic patients to the 335 peptides was evaluated, where 27.50% (11/40), 7.96% (9/113), and 32.96% (60/182) of TcMUC, MASP and TS derived-peptides were reactive, respectively (Fig. 6). Even though the percentage of reactive peptides from TcMUC and TS was similar (Fig. 6A and B), 9 from the top 10 most reactive peptides were from the TS family (Fig. 6C), reinforcing the relevance of TS reactivity in human chronic infections.

## DISCUSSION

In this work, we presented a new methodology to study the sequence variability and copy number variation of multicopy genes in complex genomes. This read-based approach is *de novo* assembly independent and does not require gene-specific read mapping, reducing the impact of collapsed genomic regions in copy number estimations. Here, it was applied to the study of the variability of the three largest T. cruzi multigene families, MASP, TcMUC, and TS, which are important virulence factors in the parasite, providing an unprecedented resolution in the study of T. cruzi multigene families. We showed that these families vary among and within the parasite DTUs and were differently recognized by the host immune system.

The study of T. cruzi multigene families has always been challenging, due to their large expansion in the parasite genome and highly repetitive content (8, 10, 12). Even after the sequencing and assembly of T. cruzi reference genomes (8, 11, 13–15, 18, 24–26), some genes from these families were still collapsed (Fig. S1) and read-mapping programs are not able to assign gene-specific mapping for a considerable proportion of the reads. To deal with multi-mapped reads, researchers opt for one of three approaches. (i) Remove reads that map in more than one gene, which underestimates gene counts; (ii) Report all matches of reads that have a similar matching score, overestimating counts in some genes; (iii) Let the mapping program randomly assign the reads to genes with the same matching score, which could inflate counts from low copy genes and deflate counts for high copy genes (27). The proposed methodology overcomes these issues because it does not require gene-specific mapping. In addition, the use of reads instead of assembled sequences/genes as the unity of comparison was very informative because it contains the entire repertoire of sequences, including potentially collapsed repetitive sequences in genome assemblies, and allelic variants, and accounts for recombined and mosaic genes. Finally, it allows the assessment of the variability in a wide range of isolates that have sequencing read libraries but no genome assembly. This methodology is, however, affected by the quality of the reference genomes where miss-annotation and miss-assemblies may impact the variability and copy number estimations. Because the T. cruzi reference genome does not have telomere to telomere sequence assemblies to all chromosomes, this may result in some missing genes and underestimated variability. Nevertheless, the methodology was robust to compare the copy number and variability of multigene families among isolates in relation to a reference genome.

T. cruzi multigene families are composed of conserved gene blocks, as seen for the MASP MEMEs, in which motifs vary in size between 8 (motif 3) to 50 (motif 14) amino acids, as well as in copy number and ordering among genes (8). Hence, the shortest MASP motifs (~8 to 10 amino acids, which are equivalent to 24 to 30 nucleotides) are considerably smaller than the median 100 nt Illumina sequencing read length. Hence, we generated 30 nucleotide-long k-mers from the reads and clustered them by global similarity to remove redundancy. Clustering was important to minimize the impact of subtle sequence variations that are expected in these long-diverging parasites (28–30). The relevance of the clustering became clear when the numbers of shared k-mers and clusters (motifs) among all parasite DTUs were compared. While only ~0.25 to 0.6% of the exact k-mers were found in the 36 T. cruzi strains/isolates, around 20 to 43% of the clusters were shared among them (Table 1). This showed that, although variable at the nucleotide level, T. cruzi multigene families are formed by several conserved motifs. Because they were shared among 36 strains, these conserved motifs could be important to the core functions of each family, and the antigenicity of some of them was evaluated in the present work.

TcI strains presented a reduction in k-mer variability, and TcI and TcII in cluster copy numbers compared with TcIII-TcVI strains (Fig. 2 and Table S1). The lower number of different clusters but similar copy numbers in TcI compared with TcII suggested that the expansion of multigene families in TcI is mainly caused by redundant sequences. The low copy number of TcMUC and MASP clusters observed in some TcII strains as S11 and S154a and the low copy number for all three families in S23b and S44a (Table S1), associated with a high cluster variability (Table S1), suggested that these isolates have variable low copy clusters. This assumption was also supported by their low k-mer/cluster ratio. Taken together, these results suggested that TcI strains have an expansion of redundant clusters with lower variability, compared with TcII-TcVI strains. These results are in accordance with the study by Cerqueira et al. (31), which showed greater intragenomic conservation of some multigene families in TcI isolates compared to TcII isolates. The overall smaller content of multigene families in TcI, compared to TcVI hybrid strains, is also in accordance with previous studies that compared multigene family’s content between TcI (Dm28 and Sylvio) and TcVI (CL Brener and TCC) strains (8, 11, 13, 18).

The patterns of cluster variability and copy number grouped T. cruzi strains by DTU (Fig. 3). Because these genes are directly enrolled in host-parasite interaction processes, variation in their functional motifs could be important to the potentially different niches occupied by the parasite DTUs (20, 22). Differences observed in cluster variability (Fig. 3A to C) and copy number (Fig. 3D to F) could be caused by several factors, such as (i) strain or DTU-specific motif sequences; (ii) gene and segmental duplications and genome size; (iii) chromosomal somy variation patterns, and (iv) in the case of hybrid DTUs, different resolution of the hybridization events. The latter is supported by the different clustering of hybrid DTUs based on cluster presence or copy number (Fig. 3). Alternatively, this pattern might be generated by different hybridization events. While a strong correlation was observed between the genome size and TS cluster counts, only moderate association was detected for MASP and TcMUC (Fig. 4), suggesting that expansion of these multigene families may have differential contributions to T. cruzi genome size variations. We observed a higher copy number of clusters in hybrids compared to nonhybrids strains (Fig. 4B), which is in accordance with previous studies that showed a larger gene count of multigene families in CL Brener and TCC (both TcVI hybrid strains) compared with SylvioX10 and Dm28 (both TcI) (11, 13). It is important to note that the evaluated TcIII and TcIV nonhybrid DTUs had a similar copy number to the hybrid strains. A higher number of isolates from TcIII-TcVI DTUs are needed to correctly evaluate the correlation between genome size and multigene family copy numbers in all T. cruzi groups.

There were strong correlations between the genome size and cluster variability in T. cruzi strains for all three gene families (Fig. 4C and D). Hybrid strains had a statistically significant higher variability of multigene families compared with nonhybrid isolates, showing that they retain a larger variability of the multigene family’s repertoire. This was observed even in cases where the overall copy number was not different from the observed in TcI and TcII, as in Tulahuen (TcVI). After the hybridization, there could be a selective pressure to maintain a broader set of variant multigene families, keeping alleles from both parental strains, which is in accordance with a process of hybridization followed by temporary tetrasomy and genome erosion, recently confirmed to occur in T. cruzi (32). This assumption is further supported by the synteny loss in genomic regions encoding multigene families when comparing both haplotypes in CL Brener and TCC hybrid strains (8, 11). The SRR3676277 strain presented a pattern of cluster copy number and variability comparable to hybrid strains, corroborating the phylogenetic analysis based on the single-copy genes that classified it as a TcVI (Fig. 1). However, its estimated genome size was smaller than the observed in the evaluated TcV and TcVI strains, which suggests that it could be a result of a different hybridization event.

As surface proteins are important targets for the host’s humoral immune response (20, 33–35), we evaluated the antigenicity of k-mers-encoded peptides. We were able to identify antigenic determinants in these multigene families that are simultaneously present in isolates from all T. cruzi DTUs, providing a unique data set for the comparison of the host immune response across the parasite subgroups. The host humoral immune response against k-mers-derived peptides was initially evaluated using sera from mice infected with strains from TcI, TcII, and TcVI DTUs (Fig. 5), as they are the most prevalent T. cruzi DTUs associated with human infections (22, 23). A similar proportion of peptides were reactive to the sera of the mice infected with at least one T. cruzi strain, both in the acute (62.38%) and chronic stages (~65.37%). However, there was a lower count of peptides that were simultaneously reactive to the sera of the mice infected with all three T. cruzi DTUs in the chronic (33.13%) compared to the acute (38.80%) stage. This suggests that there are core conserved sequences in these families, which are constantly recognized by the host immune system. With chronification, there is an increased diversification in the antigen’s recognition among the parasite’s subgroups (Fig. 5C). It is known that during T. cruzi’s initial infection, a complex combination of different variants of multigene family’s proteins is coexpressed resulting in a “smoke screen” effect, where the host immune system must simultaneously respond to variable immunogenic antigens, leading to a diffuse immune response (36). This phenomenon, caused by the large combination of variable antigens, could be one of the reasons for the long acute phase observed in T. cruzi infection (37).

Next, the reactivity of each multigene family was evaluated separately (Fig. S5). While the proportion of TS peptides reactive to the sera of mice infected with the three DTUs slightly increased from 65.62% to 69.17% with chronification, MASP reactivity had a drastic reduction from 54.55% to 20.54%. This reduction was accompanied by the highest DTU-specific reactivity (Fig. 5), which suggests the occurence of variations in the pattern of MASP being expressed during chronification or a selective IgM to IgG class switching. This could be important to parasite survival and chronification, as parasites expressing different variants could evade the host’s immune response. Changes in MASP expression were already reported after several passages in culture cells and mice (38, 39), and a dispersed immune response for MASP antigens was observed in high-density peptide arrays (33). These peptide arrays have shown that the most antigenic MASP motifs are localized in its central hypervariable region, which is in accordance with a potential immune evasion function for the family (33).

A reduced immune response to MASP and enhanced immune response to TS during the chronic stage of the infection were also observed in the sera of chronic Chagasic patients. Only 8% of the MASP peptides were reactive with the sera of human patients in the chronic stage of the disease, compared to reactivity levels of, respectively, 27.5% and 32.96% of the TcMUC and TS (Fig. 6B). Even though a comparable proportion of TS and TcMUC were reactive to the sera of human patients in the chronic stage, the reactivity score of the TS peptides was higher, where 9 of the top 10 most reactive peptides were from the TS family (Fig. 6C). The higher reactivity and conservation of immune response to TS during the chronic stage reinforces that immune response against this family is important for controlling T. cruzi infection. Antibodies inhibiting TS function are important for parasite control (40, 41), and TS members were already proposed to be used in the Chagas disease serodiagnosis (41), and vaccine candidates (42–44). TS sequences that are widespread among family members and conserved among DTUs could constitute a promising vaccine for Chagas disease, circumventing the lack of cross-DTU protection (45).

Taken together, the approach presented here allows the study of the variability of multigene families based only on genomic read libraries and a reference genome. It does not require gene-specific read mapping and allows a comparison of the variability of gene families of many isolates that have sequencing read libraries but no isolate-specific genome assembly. By using this methodology, we showed that clusters from TcMUC, MASP, and TS multigene families vary among and within DTUs, where hybrid strains present a higher variability compared to nonhybrid strains. The impressive repertoire of different motifs derived from these surface proteins may allow the parasite to explore a large range of hosts and niches (46). In addition, albeit these families were variable, they contain core sequences that are conserved in all evaluated strains. These family signatures could be important for the protein function, participate in crucial host-parasite interaction processes, and could be potential targets for vaccine or diagnostic tests for Chagas disease. The sequencing of other T. cruzi isolates, especially from the less studied TcIII, TcIV, and TcV DTUs could help the elucidation of the real extent of variability of all multigene families among T. cruzi DTUs. The genome sequencing, assembly at the chromosomal level, and careful annotation of the genomes of T. cruzi close-related parasites, such as T. rangeli and Tcbat (19, 47), which were also present these multigene families but are nonpathogenic or low pathogenic to humans, could contribute to a better understanding of the parasitism evolution in trypanosomatids. RNA-seq and proteomic analysis could also be used to evaluate if the expansion of clusters in these families results in increased gene expression and protein levels, providing functional relevance for these genomic expansions. Finally, the approach proposed here can also be used to study the variability of multicopy genes in any organism.

## MATERIALS AND METHODS

### Ethics statement.

All the design and methodology involving mice were performed in accordance with the guidelines of Colégio Brasileiro de Experimentação animal COBEA, strictly following the Brazilian law for “Procedures for the scientific use of animals” (11.794/2008) and were approved by the animal-care ethics committee of Universidade Federal de Minas Gerais (protocol number 143/2009).

The study protocol involving human samples was approved by the Ethics Committee of the Universidade Federal de Minas Gerais (UFMG) under protocol number 0559.0.203.000-11. All subjects provided written informed consent before blood samples were collected.

### Whole-genome sequencing libraries acquisition and processing.

A total of 36 whole-genome sequence read libraries were downloaded from the NCBI’s Sequencing Read Archive (SRA), with at least one representative of each of the six T. cruzi DTUs. The full description of the T. cruzi strains can be seen in Table S1. The selection of the read libraries was based on the four criteria: (i) sequenced using Illumina technology; (ii) reads must have a size between 100 and 150 nucleotides; (iii) genome coverage of at least 30×; and (iv) at least 80% of its reads mapped to the T. cruzi CL Brener reference genome. The accession number, genome coverage, total number of reads and read length of each library are described in Table S1. The evaluation of the read quality, estimation of genome coverage, and size were performed with standard tools and are described in Text S1. The phylogeny of the T. cruzi isolates was estimated by maximum likelihood, using the concatenated partial sequence of 1,563 single-copy genes (Text S1). To evaluate if there were multigene family genes collapsed in current genomes assembled with long reads, the coverage of each position in each chromosome was evaluated using reads from TcI, TcII, and TcVI isolates (Text S1).

### Generating k-mers from each T. cruzi multigene family.

To generate representative k-mers for each of the three major T. cruzi multigene families MASP, TcMUC, and TS, each WGS read library was mapped, using BWA-MEM.v.1.22 (48) in a representative reference genome file, containing the combination of (i) all the assembled CL Brener genomic regions (41 pseudochromosomes from the Esmeraldo-like and Non-Esmeraldo-like haplotypes and the CL Brener unassigned contigs), and (ii) the Dm28 reference genome MBSY00000000.1 obtained from the NCBI (https://www.ncbi.nlm.nih.gov/). For each library, the reads that were mapped in genes from each of the three T. cruzi major multigene families were recovered using SAMtools v1.1 as well as the corresponding gene coordinates from the GFF annotation file. These reads were submitted to Jellyfish v2.2.4 (49) to generate and count the occurrence of 30 nt-long k-mers for each family, which was size compatible with the smallest conserved motifs that constitute these families (8). K-mer counts were normalized by genome coverage, and only k-mers with a count of at least 30% of the genome coverage and a minimum depth of 10× were used in downstream analysis.

### K-mer clustering parametrization.

To reduce k-mer redundancy and generate clusters that represent the multigene families’ motifs, the high-performance greedy clustering program UCLUSTv1.2.22 was used (50). The selection of UCLUST's best global identity cutoff to cluster k-mers was estimated using the peptide sequences of the 20 MASP MEMEs described in El-Sayed et al. (4), as a gold standard (8) (Text S1). Based on these analyses, the global identity of 0.75 was selected for downstream analysis.

### Multigene family clusters generation and comparison among T. cruzi strains.

The k-mers from each of the three multigene families generated from the 36 T. cruzi strains were clustered using UCLUST v1.2.22 with the –optimal and –nofastalign; –rev; –nucleo and –id 0.75, representing a global identity similarity threshold of 75%. Next, the copy number of each k-mer was normalized by the genome coverage, and the normalized coverage of each k-mer in a cluster was summed and assumed as the cluster copy number. To compare the different clusters in each strain, the pairwise Jaccard Coefficient (JC) (intersection/union) of clusters with counts higher than zero were estimated, and clustered by UPGMA based on Jaccard distance (1-JC), in R. To compare cluster variability and copy number among T. cruzi DTUs, the pairwise Manhattan distance of the clusters copy number from all evaluated DTUs were estimated, and clustered by UPGMA. The images were generated in R, using gplots (https://cran.r-project.org/web/packages/gplots/index.html). To compare the cluster copy number variation among the three multigene families, and the phylogeny based on the single copy genes, tanglegrams were generated for each pair of comparisons. To that end, a distance-based clustering analysis for each multigene family was generated using hclust in R, based on the Manhattan distance and the UPGMA clustering method. The comparative tanglegram for each pairwise comparison of the MASP, TcMUC, TS, and single copy genes phylogeny was generated using the dendextend tanglegram function, in R and rooted in the Tc9280 strain. Dendrograms with bootstrap support based on the cluster copy number of each strain were also generated, with the R package Pvclust (https://cran.r-project.org/web/packages/pvclust/index.html), using Manhattan distance, average clustering method, and 1,000 bootstrap replicates.

### Comparison between clusters variability in hybrid and nonhybrid DTUs.

To evaluate if there were differences in the expansion of clusters among DTU-hybrid strains (TcV and TcVI) compared to non-DTU-hybrid strains (TcI, TcII, TcIII, and TcIV), two methodologies were applied. First, to evaluate if there was an expansion in the copy number of multigene families in hybrid strains, compared with nonhybrid strains, the sum of the coverage of all clusters for each T. cruzi isolates was estimated. The sum of cluster counts directly correlates with the copy number of the most conserved cluster for each family (Text S1) and was a suitable metric to compare each family copy number among isolates. The median of the sums of clusters from nonhybrid strains was compared with the ones from hybrid strains, using the Mann-Whitney test, with a significant *P* value of 0.05 in R. To evaluate if there was an expansion in the variability of multigenic clusters in hybrid strains, the number of clusters with coverage above “0” in each strain was estimated and the values obtained for hybrid strains were compared with the values obtained for nonhybrid strains using Mann-Whitney test, with the significance *P* value of 0.05.

### Comparison between cluster copy number and genome size or somy variation.

To evaluate if the genome size impacts the cluster’s composition, the Spearman correlation between the genome size, cluster copy number, or cluster variability was estimated using Rv3.6.0. The genome size of each strain was estimated by dividing the total number of generated nucleotides (read number x read size) by the genome coverage. The total copy number of clusters was estimated as the sum of the coverage of all clusters in each strain. The “cluster variability” was estimated as the number of clusters with coverage above zero in each strain. The methodology used to compare the cluster expansions and aneuploidies is available in Text S1.

### *In silico* selection of conserved and potentially immunogenic k-mers.

The selection of conserved and potentially immunogenic k-mers-derived peptides from T. cruzi multigene families was performed *in silico*, using five steps: (i) high copy number in all T. cruzi evaluated strains; (ii) exact k-mer presence in all T. cruzi evaluated strains; (iii) k-mer encoded by complete genes; (iv) rank of the k-mer-generated peptide sequences based on B-cell epitope and disorder predictions; (v) filtering identical and highly similar peptides. A further description of each of these steps can be seen in Text S1.

### Sera libraries.

To evaluate the antigenicity of the peptides derived from multigene families, the reactivity of the selected peptides to the sera of mice infected with strains from different T. cruzi DTUs in the acute and chronic infection stages, as well as sera from chronic Chagasic patients, were evaluated. The sera from C57BL/6 mice infected with T. cruzi Colombiana (TcI), Y (TcII), or CL Brener (TcVI) strains in the acute or chronic stage, and noninfected mice were obtained. This is further described in Text S1.

To access the multigene family’s antigenicity in human infections, a total of 9 sera from chronic Chagasic patients infected with parasites from the TcII DTU (51) and the sera from 14 healthy humans (negative control), were used in immunoblotting assays.

### Spot synthesis and immunoblotting.

A total of 335 peptides were selected as above, and five control peptides were synthesized in a cellulose membrane, using an automatic synthesizer ResPep SL (Intavis) and the program MultiPep (Intavis), according to the SPOT synthesis technique described in (52). This membrane was used in immunoblotting assays with pools of sera from six C57BL/6 mice acutely and chronically infected with the T. cruzi strains Colombiana (TcI), Y (TcII) or CL Brener (TcVI); uninfected mice; or with the pool of sera from 9 chronic-Chagasic patients infected with parasites from the TcII DTU, or 14 healthy individuals. The immunoblotting and densitometric analysis are further described in Text S1.

10.1128/mbio.02319-22.9 Fig S6 Selection of the UCLUST clustering identity cutoff. To select the UCLUST clustering cutoff, the correspondence between k-mers which match the MASP MEMEs described in EL-Sayed 2005 and the generated clusters were evaluated. (A) Several parameters were estimated, such as the time spent in clustering, number of clusters generated, k-mers from different MEMEs that grouped in the same cluster, number of singlets (k-mers not assigned to any cluster), the number of clusters with less than 10 k-mers, mean and median k-mer/cluster ratios. The “Errors (%)” column corresponds to the sum of the values from the “k-mers clustered in incorrect clusters” and “Singlets”, representing the percentage of erroneous or nonassigned k-mers. (B) Density plot of the distribution of the number of k-mers in clusters for the 0.75 to 0.90% cutoffs. The 0.95% was not represented as it collapsed the image, as most of its clusters presented a low k-mer count (median 2). Download FIG S6, PDF file, 0.2 MB. Copyright © 2022 Reis-Cunha et al. 2022 Reis-Cunha et al. https://creativecommons.org/licenses/by/4.0/ This content is distributed under the terms of the Creative Commons Attribution 4.0 International license.

10.1128/mbio.02319-22.10 Fig S7 Estimating the gene copy number of multigene families: (A) Consensus sequence of all the k-mers in the most conserved cluster for each multigene family: MASP Cluster 25448; TcMUC Cluster 1488 and TS Cluster 3938. In the image, “M” represents a gap position. (B) The number of BLAST matches in genes for each consensus of the conserved clusters. Freq, number of genes with a match with the consensus sequence; Total genes: Total number of annotated genes of the family in the evaluated references; Rep (representativity), proportion of genes with matches with the consensus of the cluster. (C to E) correspond to the density of the distribution of the initial (I coord, red) and final (F coord, cyan) BLAST match coordinates, respectively, for the multigene families MASP, TcMUC, and TS. This shows that the selected conserved motifs were localized in the 5’ region of the genes, for the three families. (F) Correlation between the copy number of the most relevant cluster (*x*-axis) and the sum of coverages of all motifs (*y*-axis). (G) Multigene family’s gene size. CBEL, CL Brener Esmo-like; CBNE, CL Brener Non-Esmeraldo-like; CBUC, CL Brener Unassigned contigs; DM28C, DM28 strain. Download FIG S7, PDF file, 0.5 MB. Copyright © 2022 Reis-Cunha et al. 2022 Reis-Cunha et al. https://creativecommons.org/licenses/by/4.0/ This content is distributed under the terms of the Creative Commons Attribution 4.0 International license.
