# Supplementary material for: Accessing the Variability of Multicopy Genes in Complex Genomes using Unassembled Next-Generation Sequencing Reads: The Case of Trypanosoma cruzi Multigene Families
Source: mBio. 2022 Oct 20;13(6):e02319-22. doi: 10.1128/mbio.02319-22 (PMC9765020; doi:10.1128/mbio.02319-22)

S1 Figure: Evaluation of collapsing of multigene families' sequences in different *T. cruzi* genome assemblies. In this image, three genome assemblies were evaluated. Dm28 (TcI) (first line) and Ycl6 (TcII) (second line) were assembled with a combination of long and short reads, while CL Brener Nonesmo (TcVI) (third line) was assembled with Sanger reads. The represented chromosomes/scaffolds are: DM28: PRFA01000011; Ycl6: Chr3; CL-Brener Nonesmo: Chr23. The majority of the other chromosomes had a similar pattern (next slides). Three whole genome sequencing read libraries were assessed, a TcI (SRR3676317 – First column), a TcII (SRR6357355 – Second column) and a TcVI (SRR6357354 – Third column) isolate. The blue line corresponds to the read depth of each position. Below, the protein-coding genes are depicted as rectangles drawn as proportional to their length, and their coding strand is indicated by their position above (top strand) or below (bottom strand) the central line. Coloured boxes represent multigene families, where yellow, green, brown, orange, blue and pink corresponds to, respectively, Trans-sialidase, RHS, MASP, TcMUC, DGF-1 and GP63. Black and grey rectangles represent hypothetical and housekeeping genes, respectively. Gaps are represented by gene-less regions with no read coverage. Even in long-read assemblies, there is still a relevant increase in the read depth of multigene families, which reinforces the need of new methodologies to better access their variability.

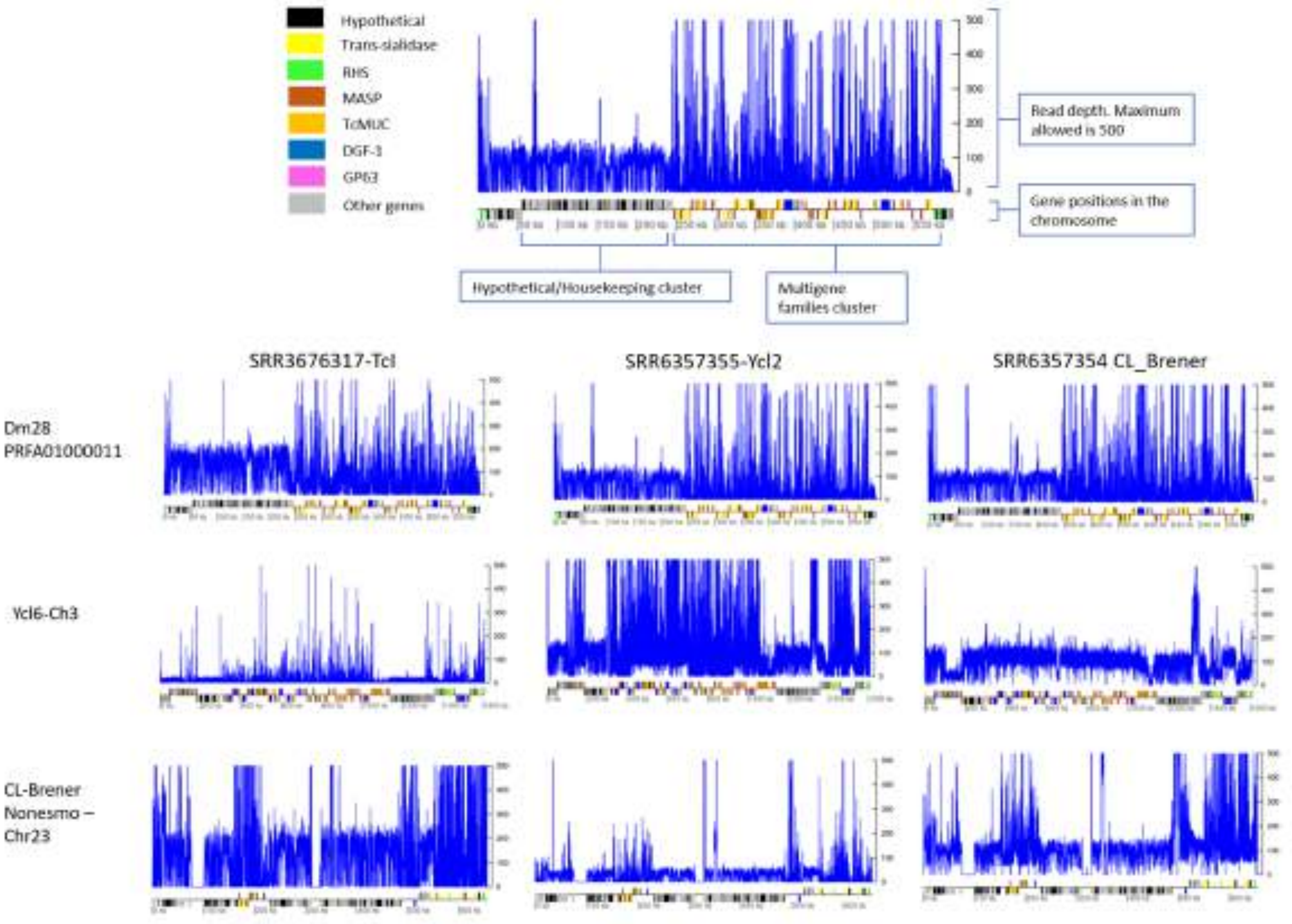

Reference - CL Brener (TcVI) - reads -TcI

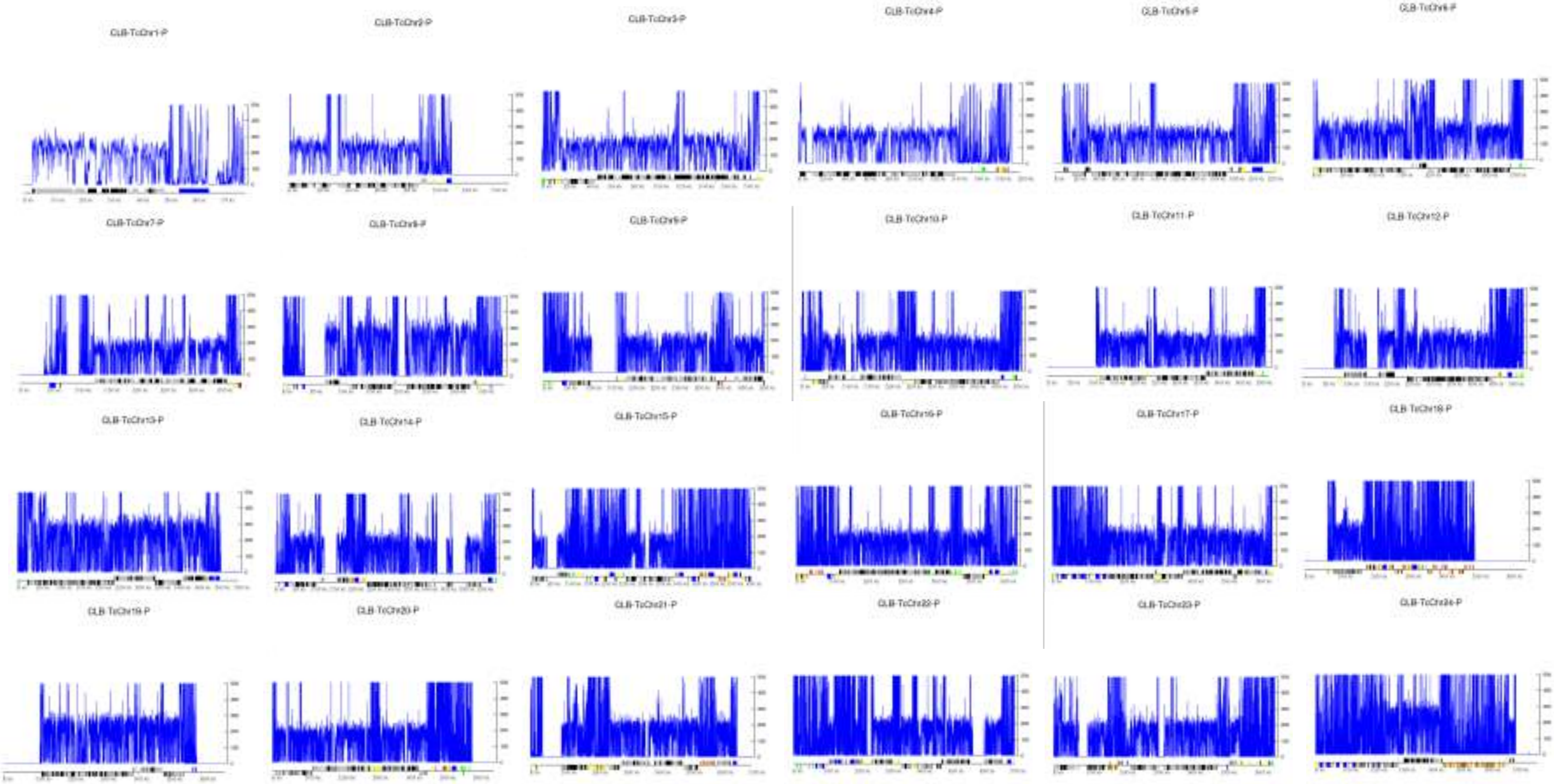

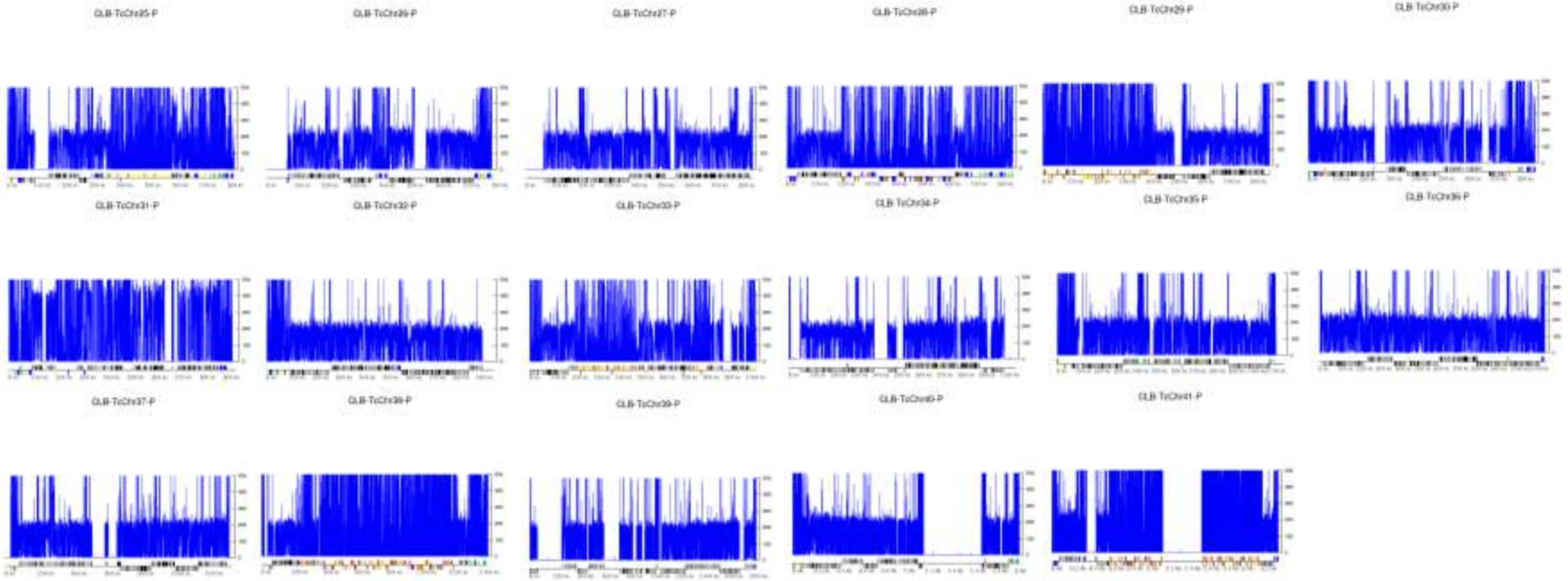
GLB-TcChr25-P
GLB-TcChr26-P
GLB-TcChr27-P
GLB-TcChr28-P
GLB-TcChr29-P
GLB-TcChr30-P
GLB-TcChr31-P
GLB-TcChr32-P
GLB-TcChr33-P
GLB-TcChr34-P
GLB-TcChr35-P
GLB-TcChr36-P
GLB-TcChr37-P
GLB-TcChr38-P
GLB-TcChr39-P
GLB-TcChr40-P
GLB-TcChr41-P

Reference - CL Brener (TcVI) - reads -TcII

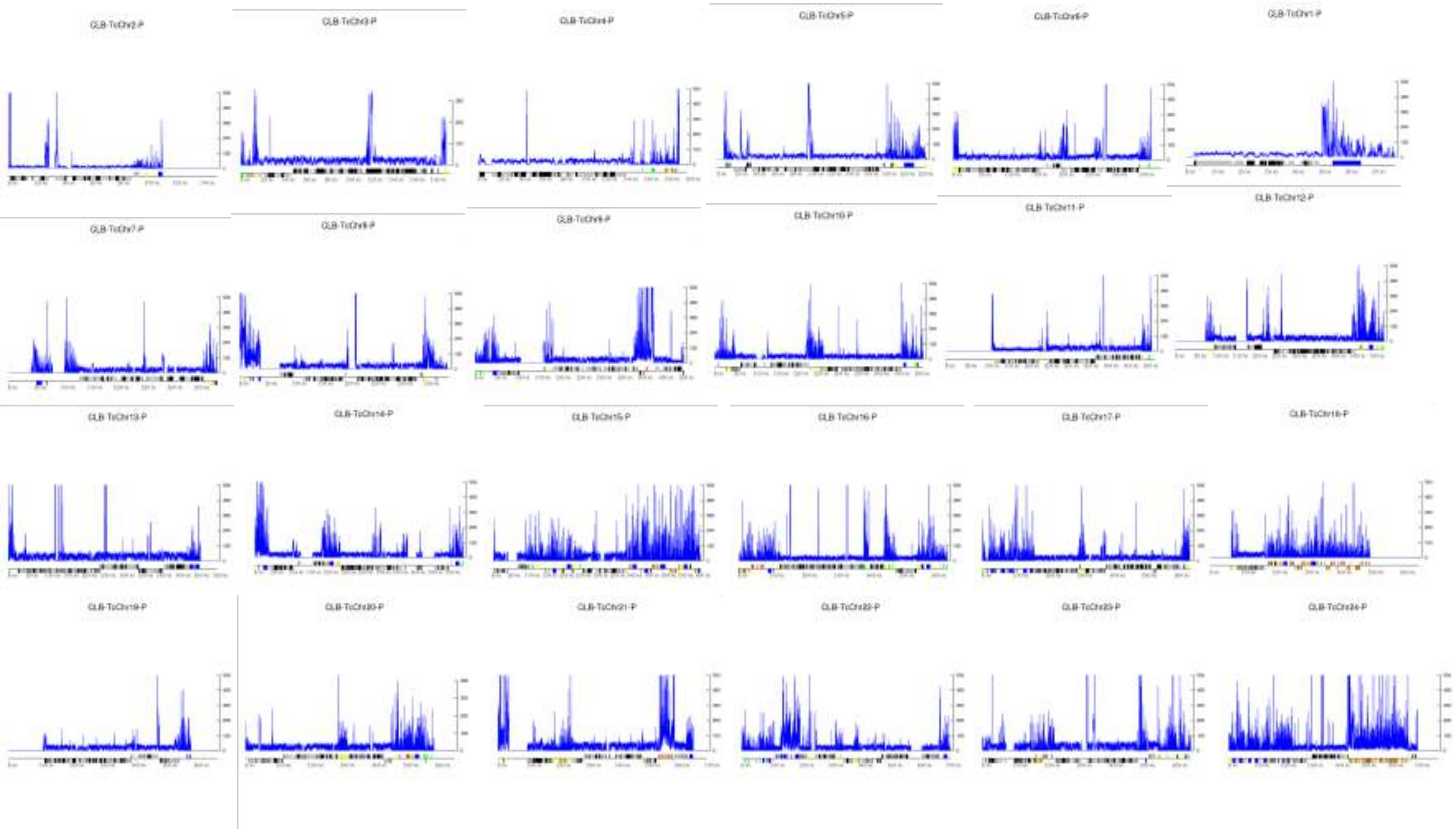

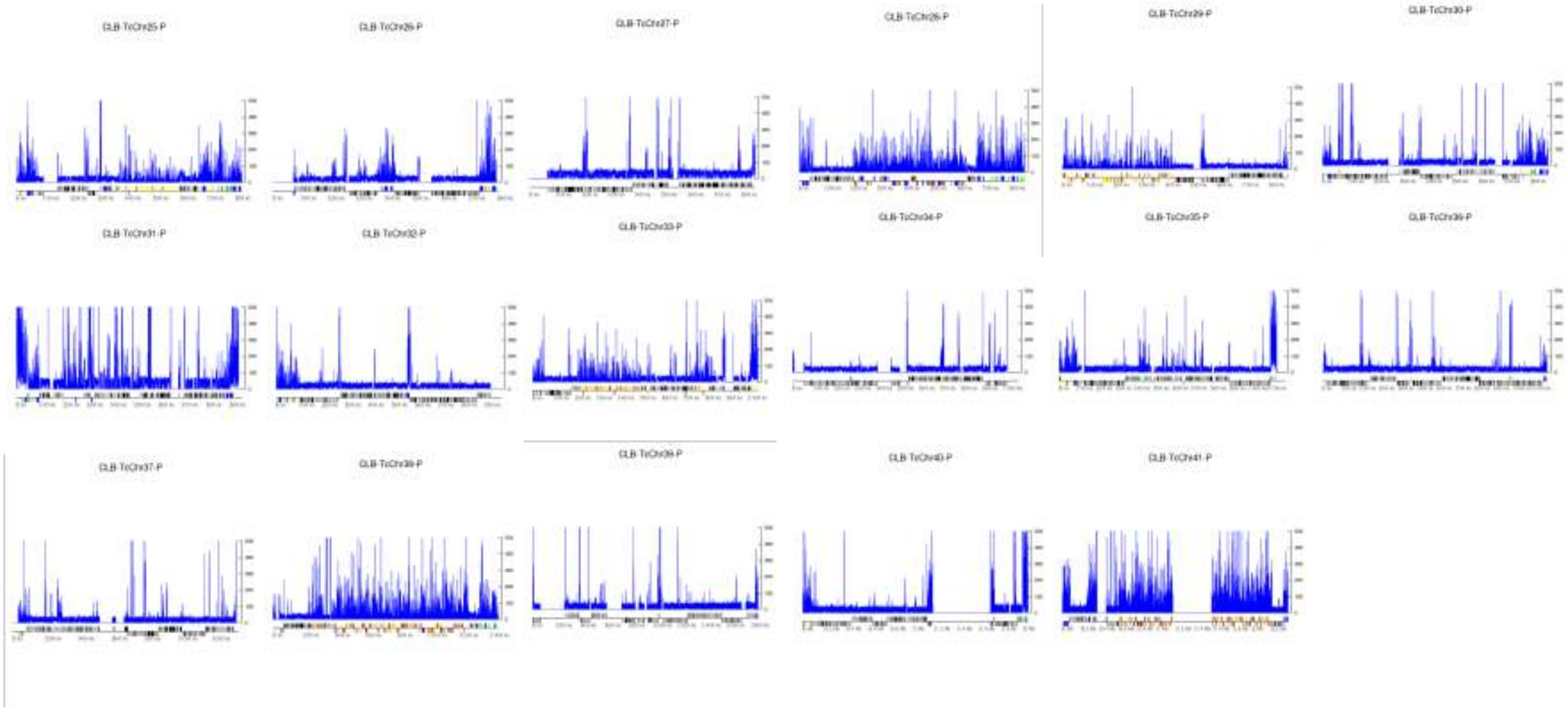
CLB-TcChr25-P
CLB-TcChr26-P
CLB-TcChr27-P
CLB-TcChr28-P
CLB-TcChr29-P
CLB-TcChr30-P
CLB-TcChr31-P
CLB-TcChr32-P
CLB-TcChr33-P
CLB-TcChr34-P
CLB-TcChr35-P
CLB-TcChr36-P
CLB-TcChr37-P
CLB-TcChr38-P
CLB-TcChr39-P
CLB-TcChr40-P
CLB-TcChr41-P

Reference - CL Brener (TcVI) - reads -TcVI

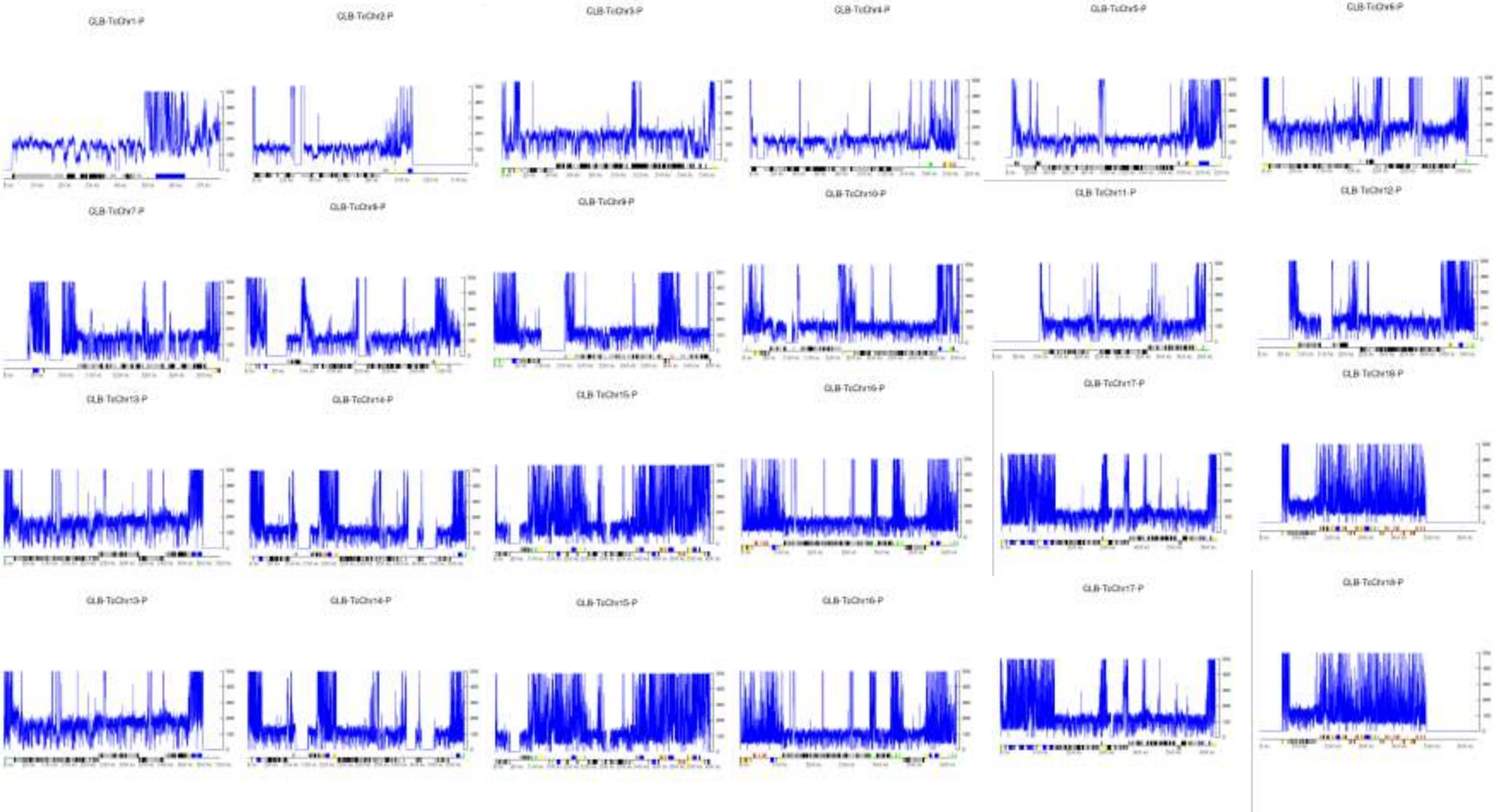

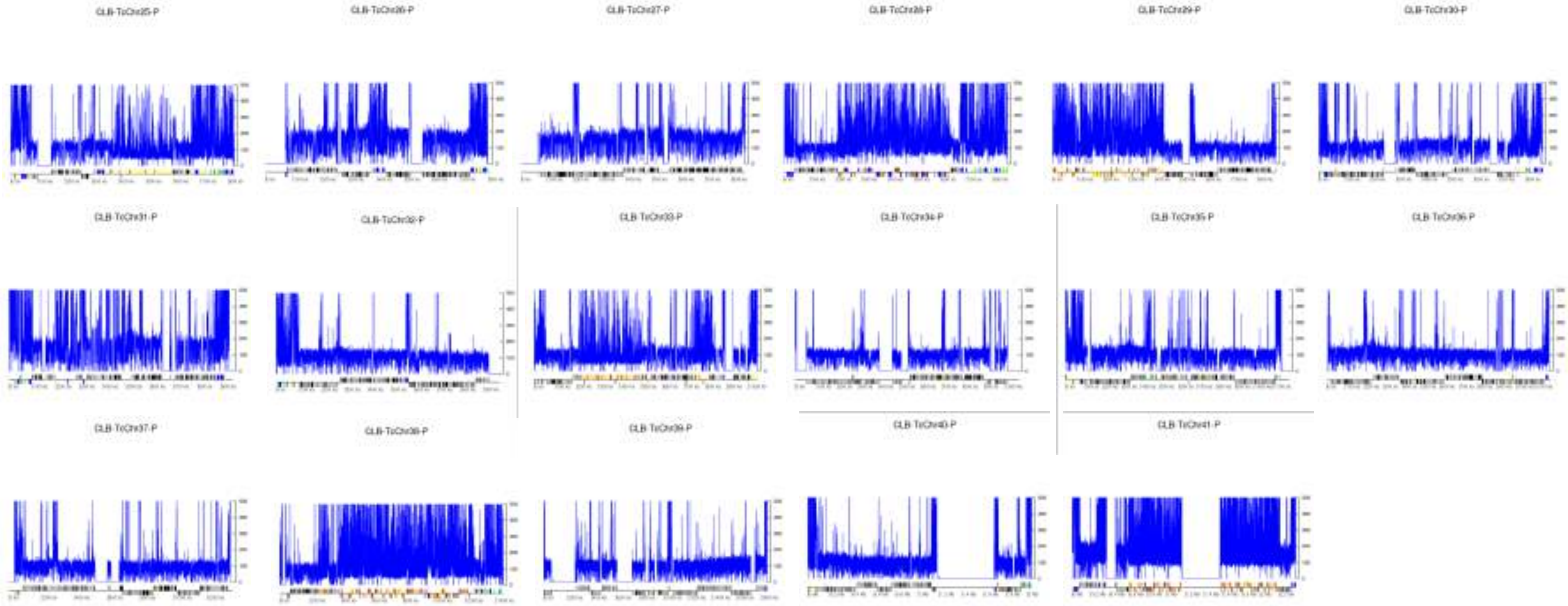
CLB-TcChr25-P
CLB-TcChr26-P
CLB-TcChr27-P
CLB-TcChr28-P
CLB-TcChr29-P
CLB-TcChr30-P
CLB-TcChr31-P
CLB-TcChr32-P
CLB-TcChr33-P
CLB-TcChr34-P
CLB-TcChr35-P
CLB-TcChr36-P
CLB-TcChr37-P
CLB-TcChr38-P
CLB-TcChr39-P
CLB-TcChr40-P
CLB-TcChr41-P

Reference - Sylvio (TcI) - reads -TcI

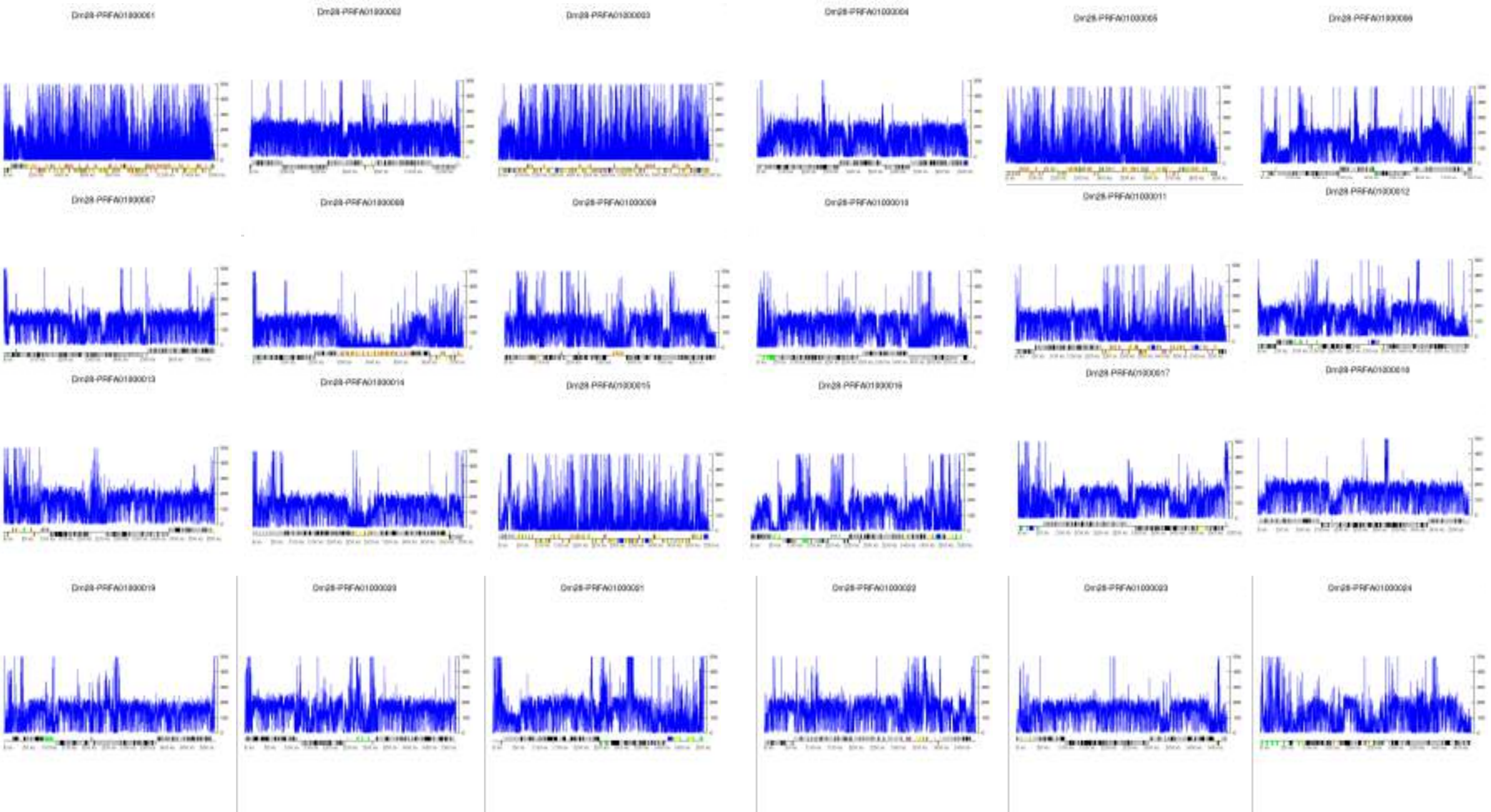

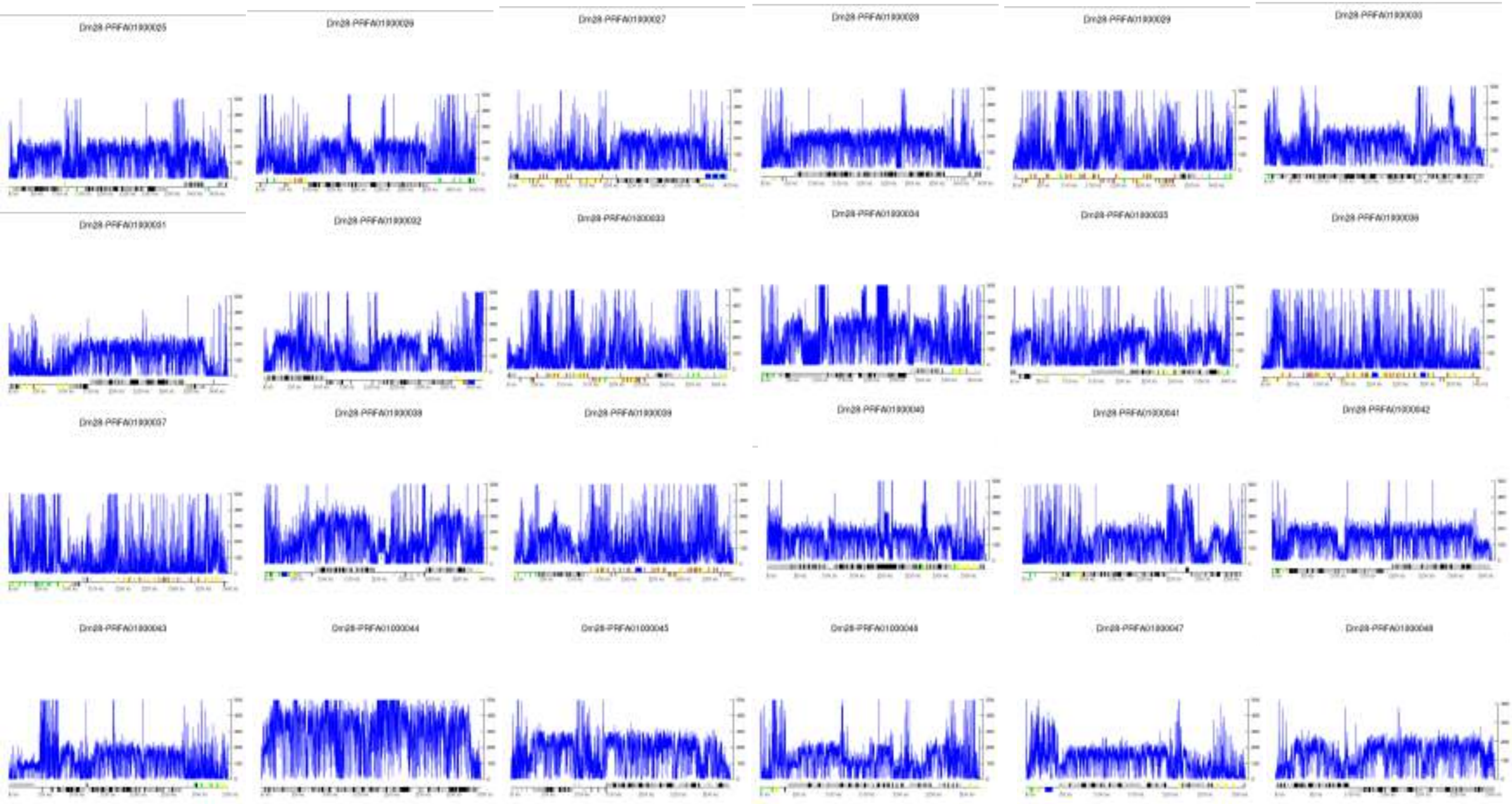
Dm28-PRFA01000025
Dm28-PRFA01000026
Dm28-PRFA01000027
Dm28-PRFA01000028
Dm28-PRFA01000029
Dm28-PRFA01000030
Dm28-PRFA01000031
Dm28-PRFA01000032
Dm28-PRFA01000033
Dm28-PRFA01000034
Dm28-PRFA01000035
Dm28-PRFA01000036
Dm28-PRFA01000037
Dm28-PRFA01000038
Dm28-PRFA01000039
Dm28-PRFA01000040
Dm28-PRFA01000041
Dm28-PRFA01000042
Dm28-PRFA01000043
Dm28-PRFA01000044
Dm28-PRFA01000045
Dm28-PRFA01000046
Dm28-PRFA01000047
Dm28-PRFA01000048

Dn28.PRFA01000049

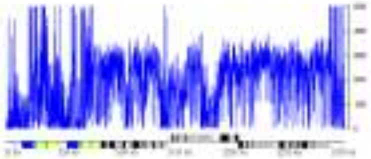

Dn28.PRFA01000050

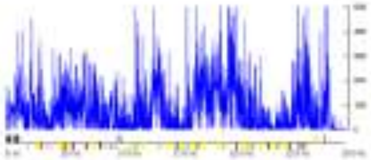

Reference - Sylvio (TcI) - reads -TcII

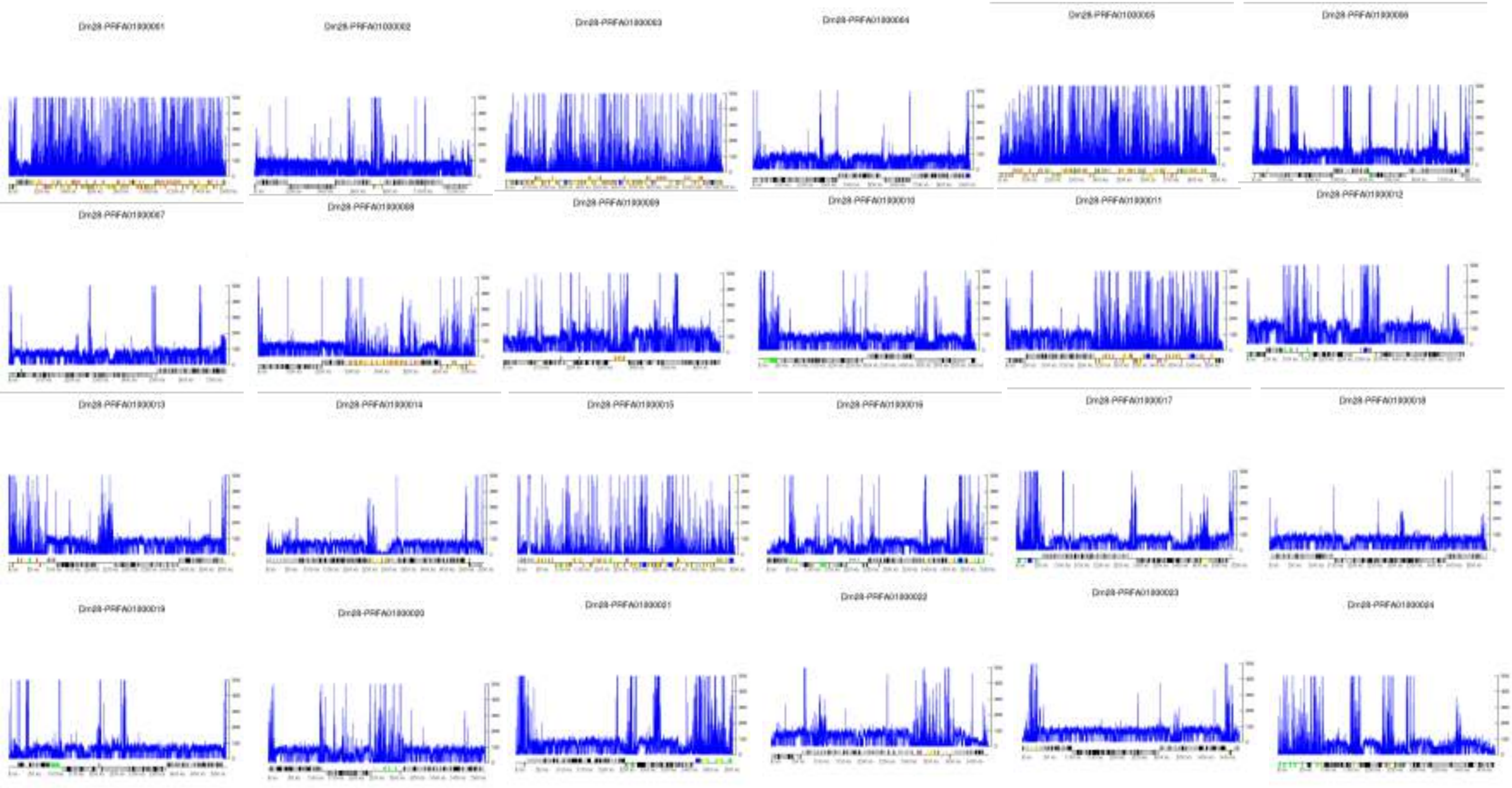

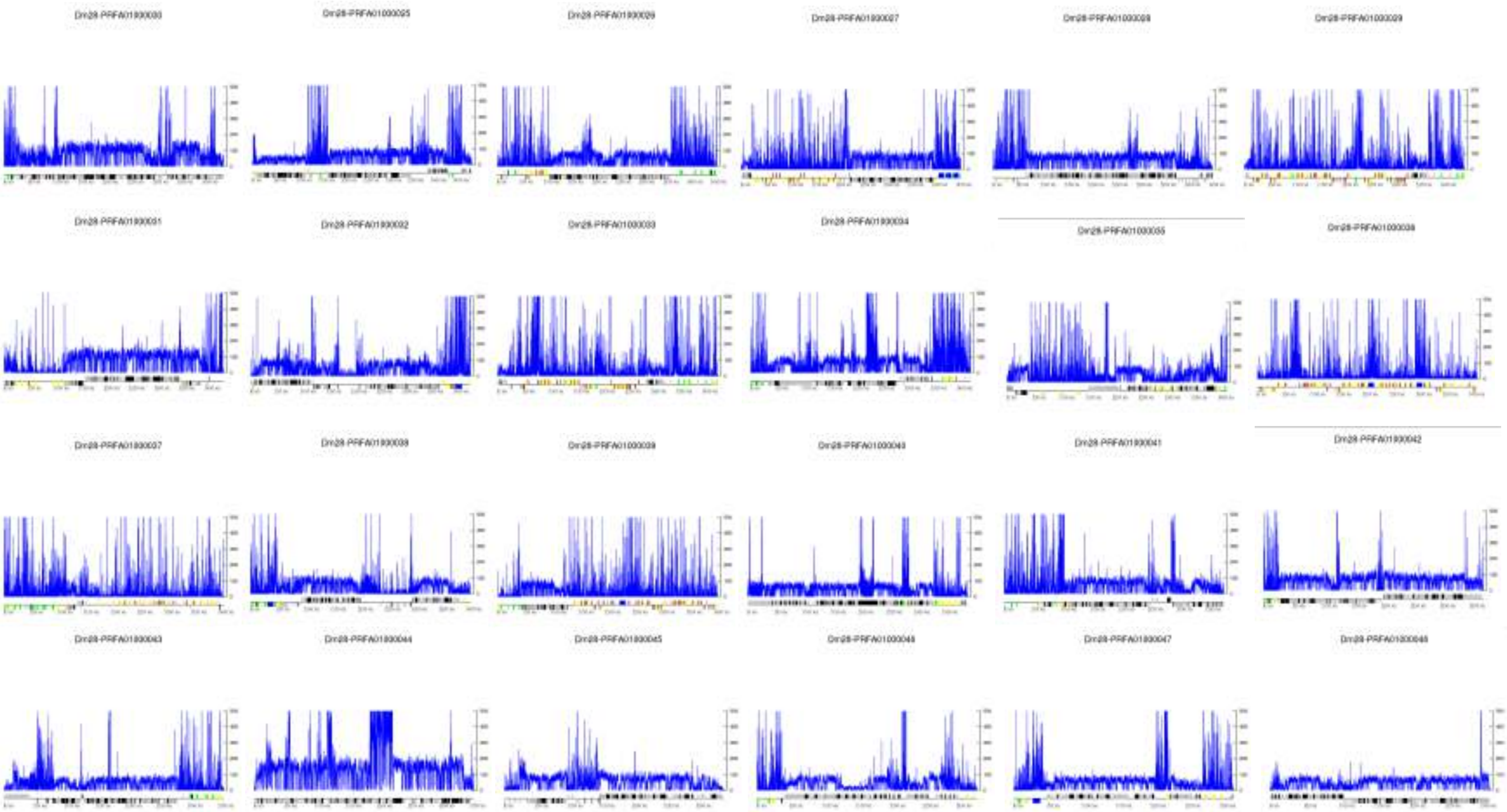
Dm28-PRFA01000030
Dm28-PRFA01000025
Dm28-PRFA01000026
Dm28-PRFA01000027
Dm28-PRFA01000028
Dm28-PRFA01000029
Dm28-PRFA01000031
Dm28-PRFA01000032
Dm28-PRFA01000033
Dm28-PRFA01000034
Dm28-PRFA01000035
Dm28-PRFA01000036
Dm28-PRFA01000037
Dm28-PRFA01000038
Dm28-PRFA01000039
Dm28-PRFA01000040
Dm28-PRFA01000041
Dm28-PRFA01000042
Dm28-PRFA01000043
Dm28-PRFA01000044
Dm28-PRFA01000045
Dm28-PRFA01000046
Dm28-PRFA01000047
Dm28-PRFA01000048

Dm28-PRFA01000049

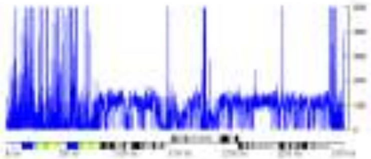

Dm28-PRFA01000050

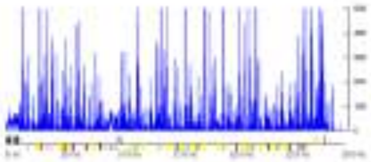

Reference - Sylvio (TcI) - reads -TcVI

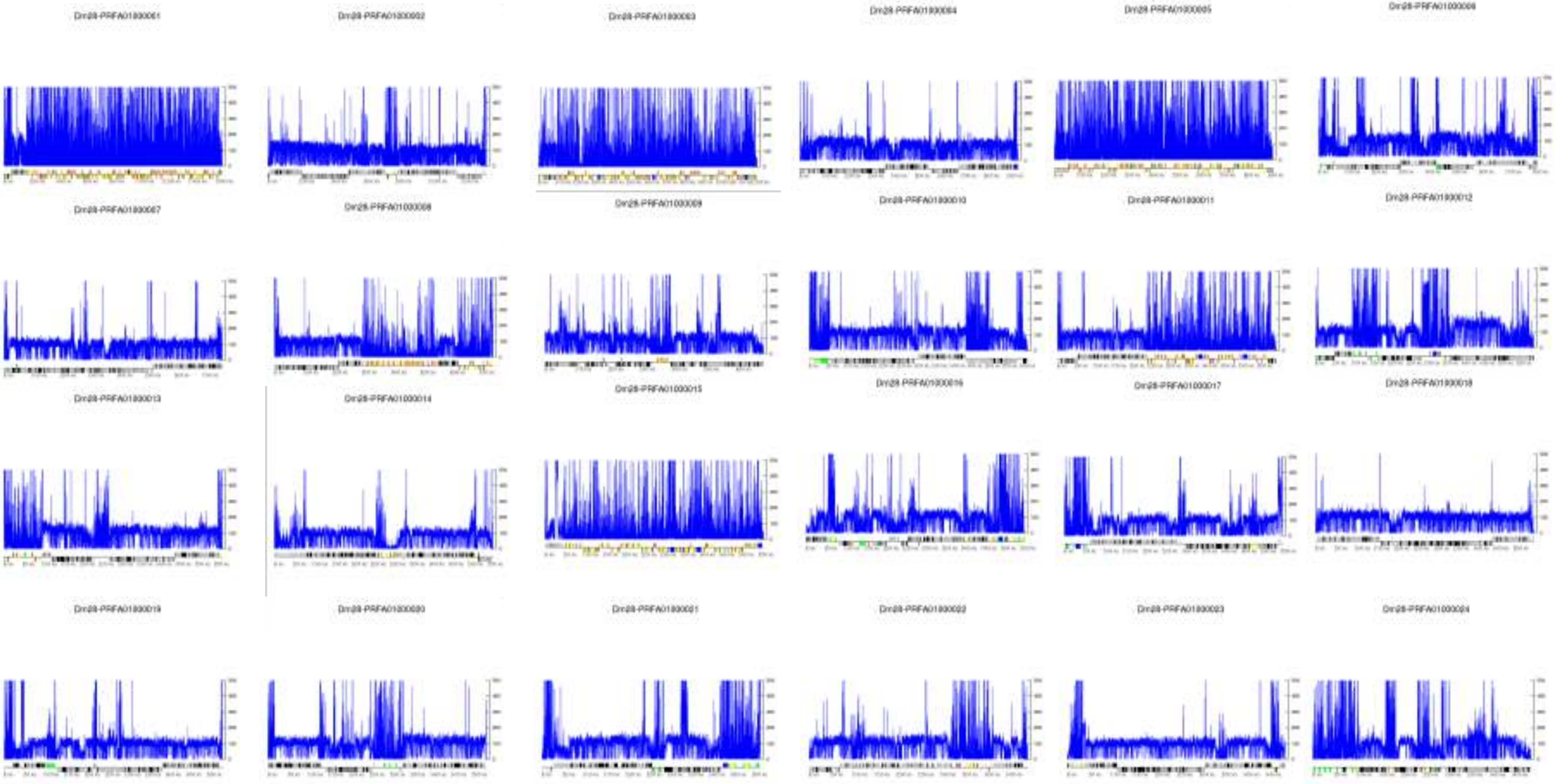

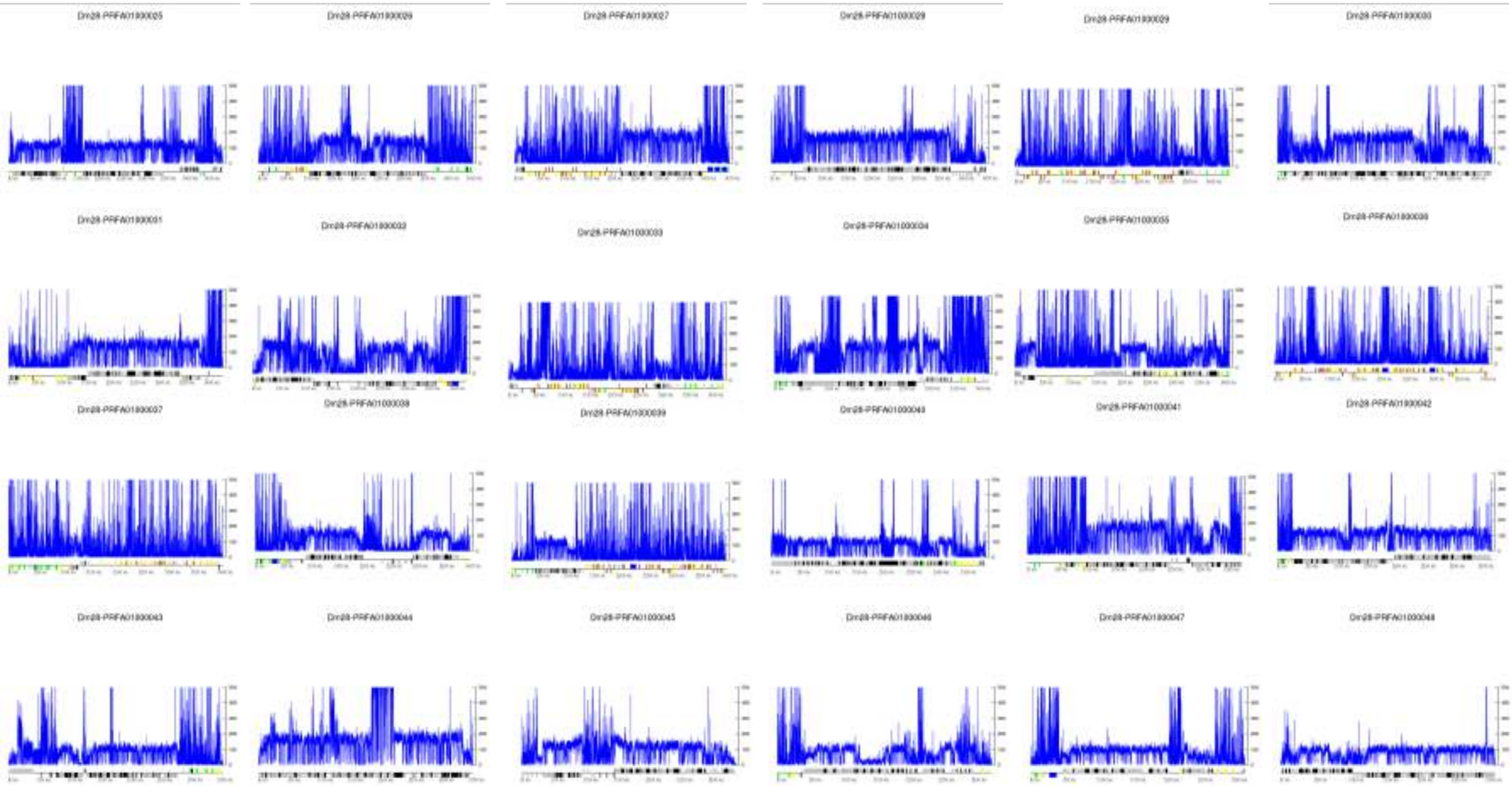
Dm28-PRFA01000025
Dm28-PRFA01000026
Dm28-PRFA01000027
Dm28-PRFA01000028
Dm28-PRFA01000029
Dm28-PRFA01000030
Dm28-PRFA01000031
Dm28-PRFA01000032
Dm28-PRFA01000033
Dm28-PRFA01000034
Dm28-PRFA01000035
Dm28-PRFA01000036
Dm28-PRFA01000037
Dm28-PRFA01000038
Dm28-PRFA01000039
Dm28-PRFA01000040
Dm28-PRFA01000041
Dm28-PRFA01000042
Dm28-PRFA01000043
Dm28-PRFA01000044
Dm28-PRFA01000045
Dm28-PRFA01000046
Dm28-PRFA01000047
Dm28-PRFA01000048

Dm28-PRFA01000049

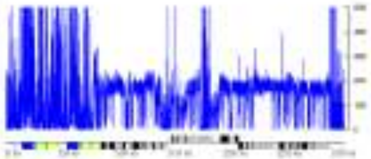

Dm28-PRFA01000050

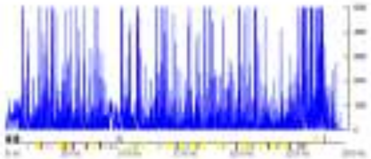

Reference - Y (TcII) - reads -TcI

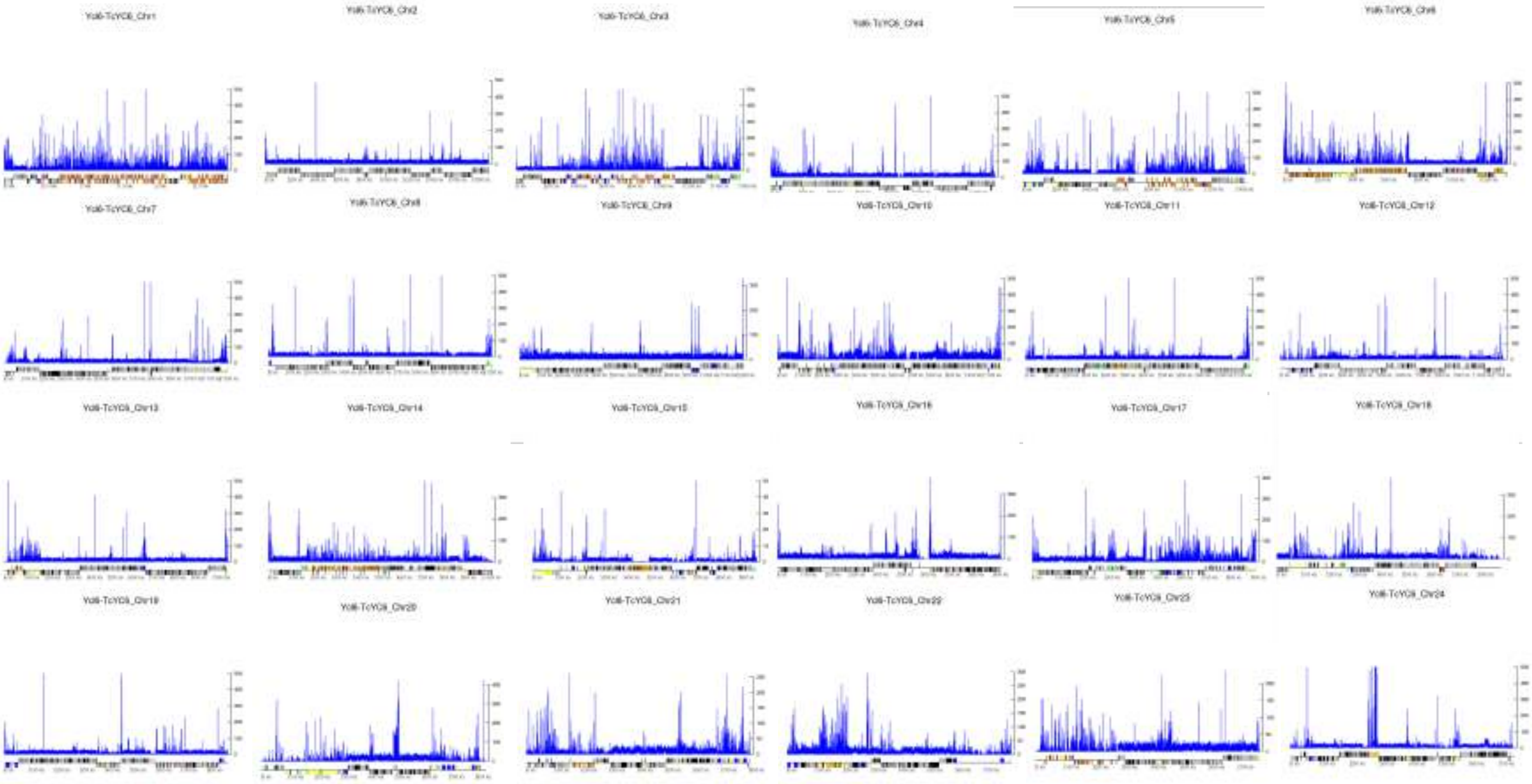

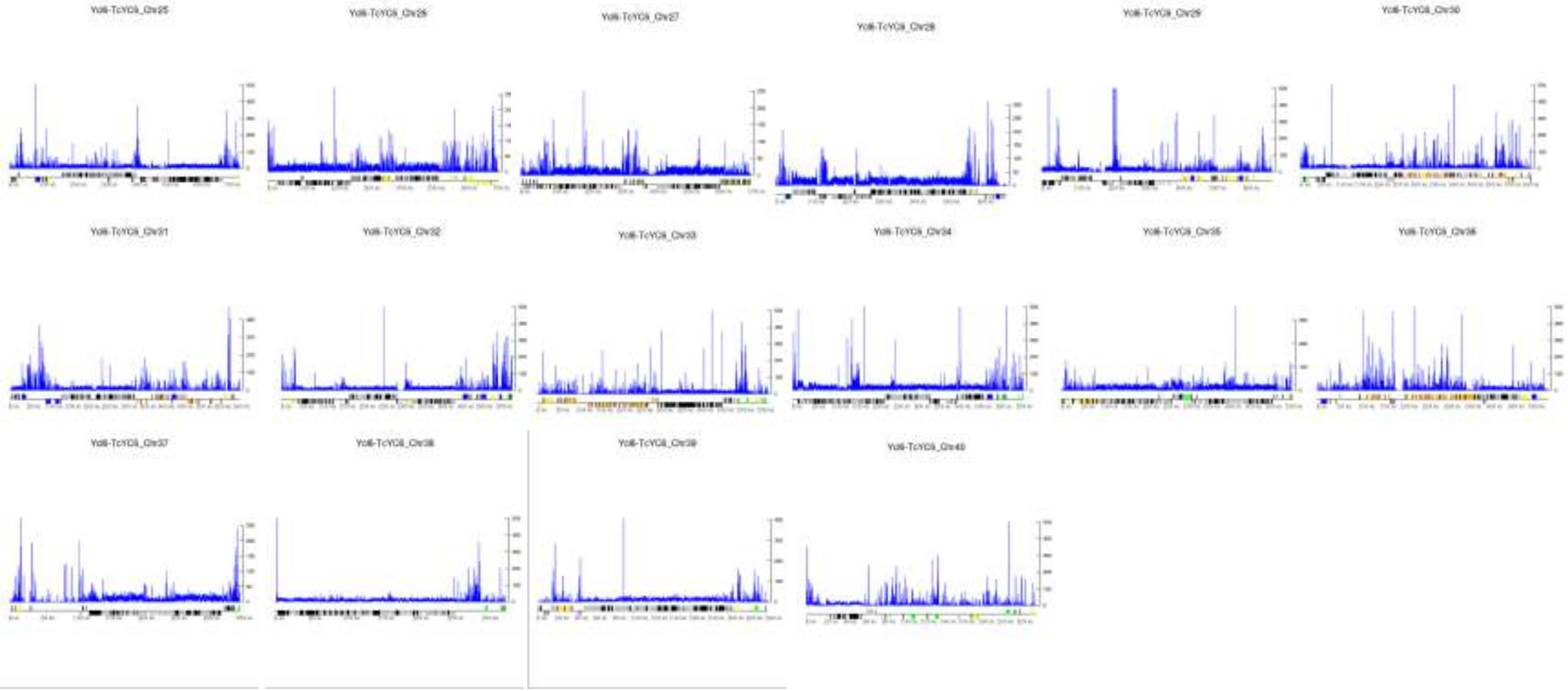
Yoli-TcYC6_Chr25
Yoli-TcYC6_Chr26
Yoli-TcYC6_Chr27
Yoli-TcYC6_Chr28
Yoli-TcYC6_Chr29
Yoli-TcYC6_Chr30
Yoli-TcYC6_Chr31
Yoli-TcYC6_Chr32
Yoli-TcYC6_Chr33
Yoli-TcYC6_Chr34
Yoli-TcYC6_Chr35
Yoli-TcYC6_Chr36
Yoli-TcYC6_Chr37
Yoli-TcYC6_Chr38
Yoli-TcYC6_Chr39
Yoli-TcYC6_Chr40

Reference - Y (TcII) - reads -TcII

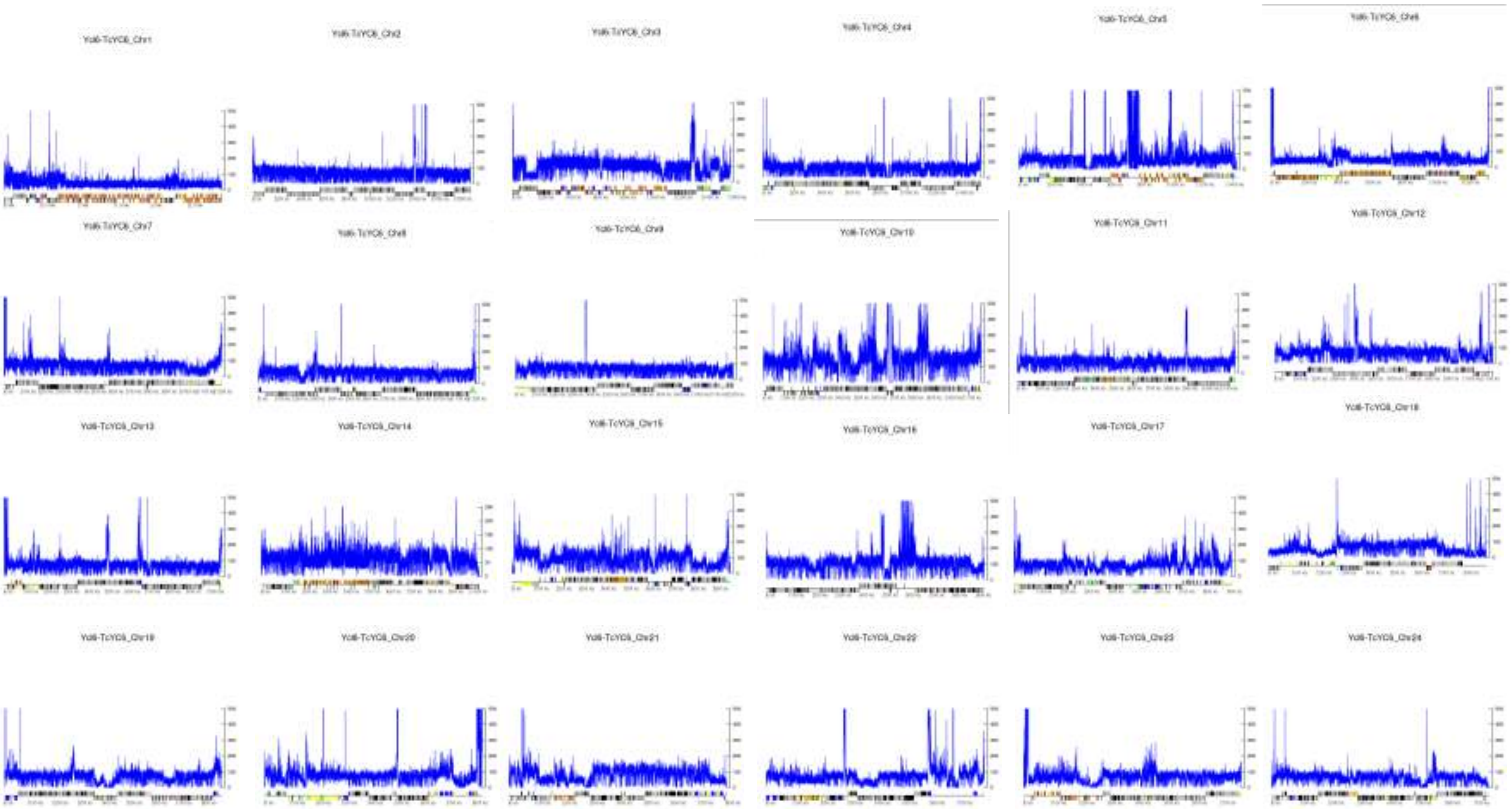

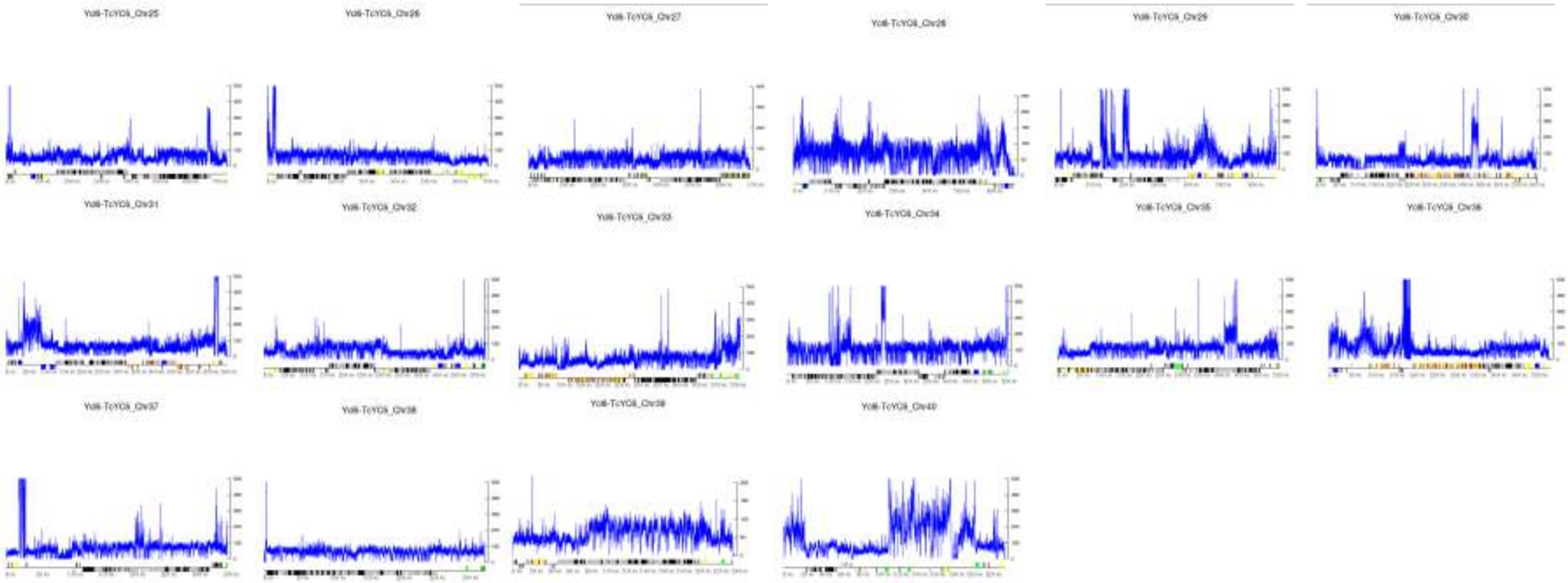

Reference - Y (TcII) - reads -TcVI

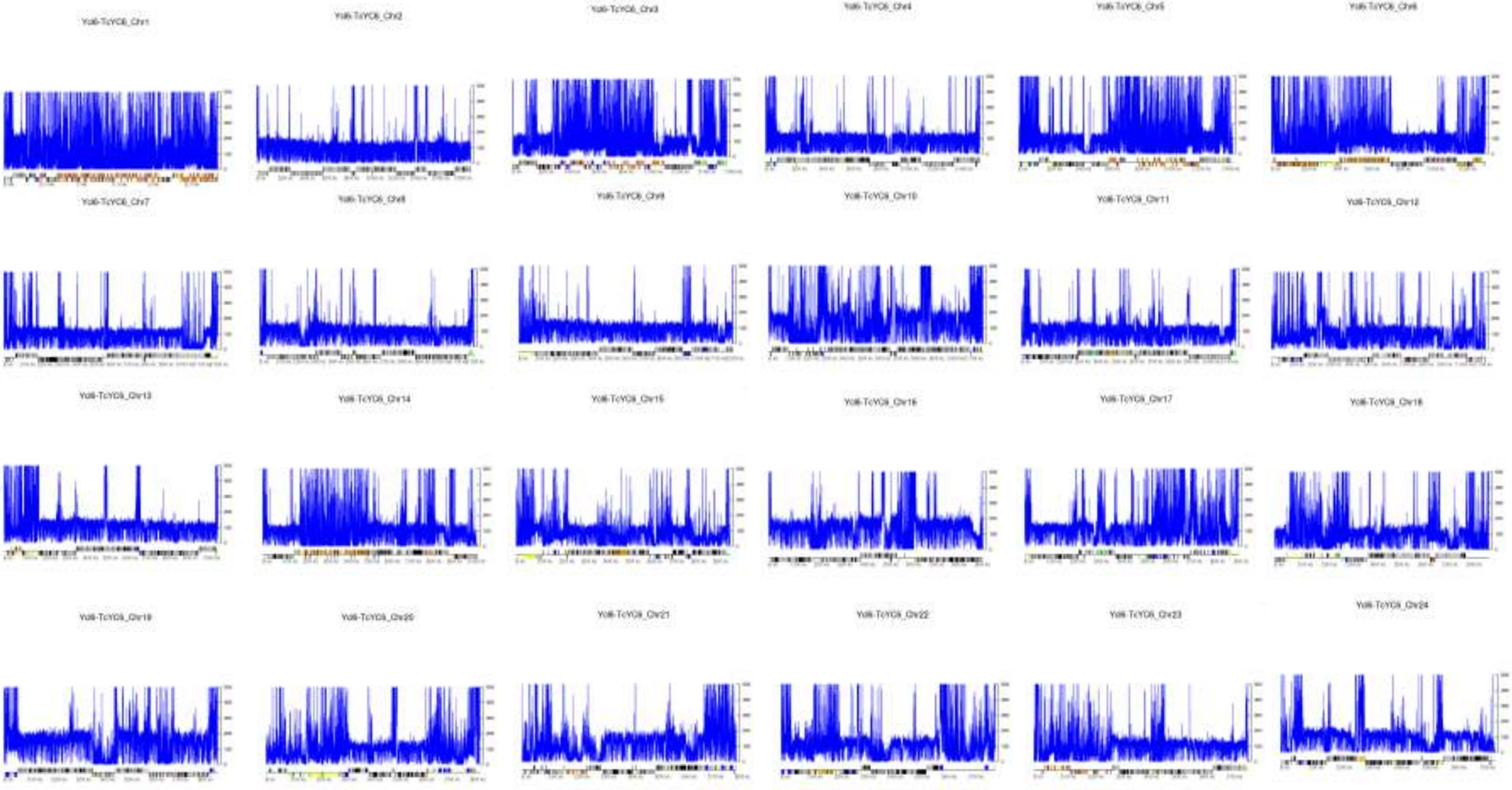

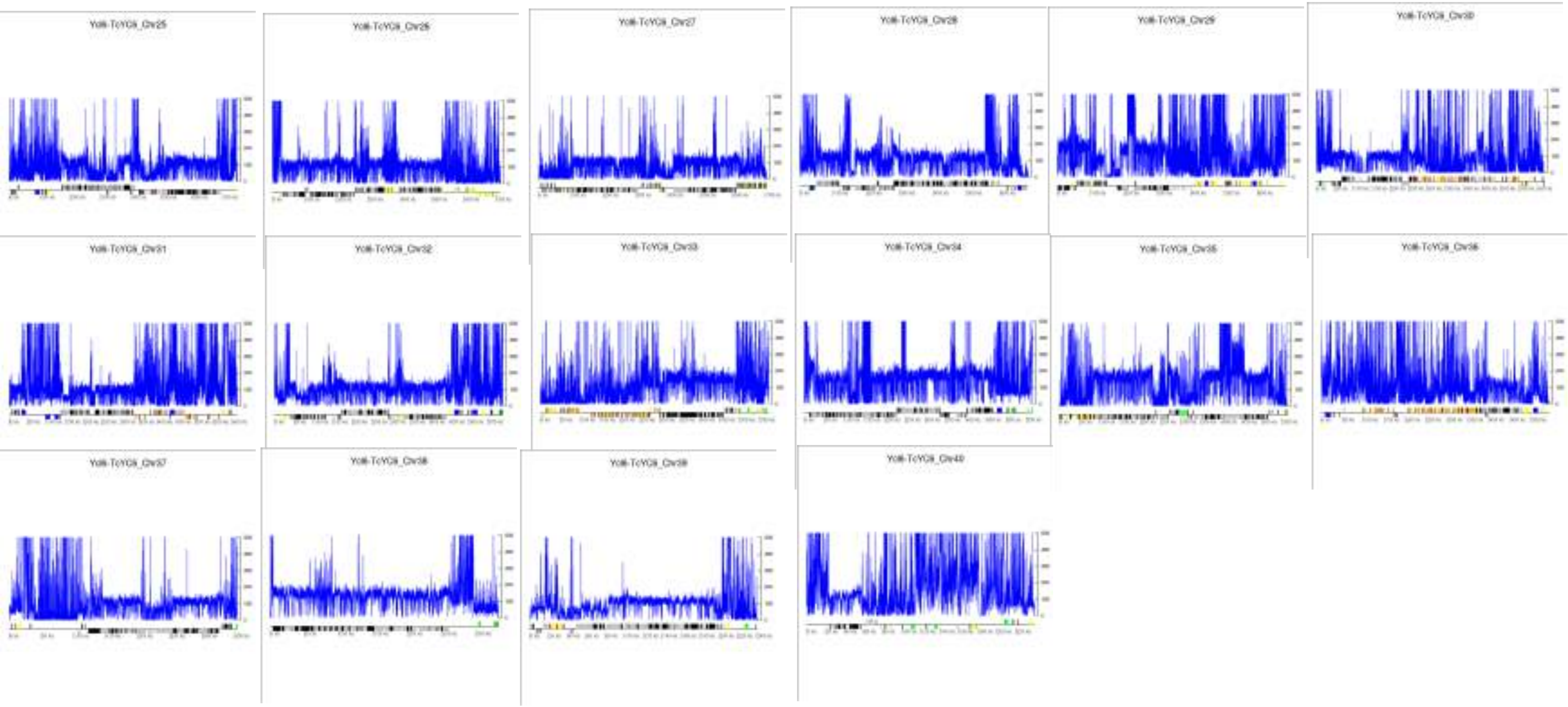

Supplement: Fig S1 [file mbio.02319-22-s0002.pdf]
