# Supplementary material for: Accessing the Variability of Multicopy Genes in Complex Genomes using Unassembled Next-Generation Sequencing Reads: The Case of Trypanosoma cruzi Multigene Families
Source: mBio. 2022 Oct 20;13(6):e02319-22. doi: 10.1128/mbio.02319-22 (PMC9765020; doi:10.1128/mbio.02319-22)
Supplement: Text S1 [file mbio.02319-22-s0001.docx]

**Supplementary methods:**

**Genome coverage and genome size estimations**

The quality of sequencing reads was evaluated with FastQC [(1)](https://paperpile.com/c/gErfVg/wnlnr) and adapter sequence and low-quality reads were removed with Trimmomatic [(2)](https://paperpile.com/c/gErfVg/9HaAH), with mean quality higher than 30 in a 5-nucleotide window and minimum size of 50 nucleotides.

The genome coverage was estimated based on the mean coverage of 1,563 single copy genes [(3)](https://paperpile.com/c/gErfVg/cBql2). Briefly, each *T. cruzi* read library was mapped in the CL Brener reference haplotype that is phylogenetically closer to it. Hence, TcI and TcIII read libraries were mapped in *T. cruzi* CL Brener Non-Esmeraldo-like reference genome version 46, while TcII, TcIV, TcV and TcVI libraries were mapped in the *T. cruzi* CL Brener Esmeraldo-like reference genome version 46, both obtained from the TriTrypDB [(4)](https://paperpile.com/c/gErfVg/d16MF), using BWA-MEM v. 0.7.12 [(5)](https://paperpile.com/c/gErfVg/70D4Z). The use of Esmeraldo-like or Non-Esmeraldo-like haplotype references has a minor impact on genome coverage estimation, as it was based on single copy genes that are conserved among both haplotypes. The mapped reads were filtered by mapping quality 30 using SAMtools v1.1 [(6)](https://paperpile.com/c/gErfVg/UEkgi), and the read depth coverage (RDC) of each region was estimated with BEDTools genomecov v2.16.2 [(7)](https://paperpile.com/c/gErfVg/bQoRX). Next, the genome coverage was estimated as the mean coverage of the 1,563 single copy genes using PERL scripts. The genome coverage of each read library is described in the S1 table. To estimate the genome size, each read library was simultaneously mapped in the *T. cruzi* CL Brener Esmeraldo-like, Non-Esmeraldo-like and unassigned contigs version 46 using BWA-MEM, encompassing all the assembled CL Brener nuclear genome regions. Next, the percentage and number of mapped reads was estimated with SAMtools flagstat. The genome size was estimated by the product of the number of reads and the mean read size, divided by the genome coverage. The estimated genome size for each isolate can be seen in the S1 Table.

To evaluate if there were multigene families’ genes collapsed in current genomes, assembled with long reads, the coverage of each position in each chromosome was evaluated using Illumina reads from TcI, TcII and TcVI isolates. The reference genomes from Sylvio (TcI), Y (TcII) and CL Brener were downloaded from TriTrypDB version 52 [(4)](https://paperpile.com/c/gErfVg/d16MF). Whole genomic sequencing reads from three isolates, SRR3676317 (TcI), SRR6357355 (TcII) and SRR6357354 (TcVI) were downloaded from NCBI SRA and mapped individually in each reference with BWA men. The coverage of each genomic position was determined with BedTools genomecov [(7)](https://paperpile.com/c/gErfVg/bQoRX), and the gene coordinates were recovered from GFF files, obtained in TriTrypDB. The images were generated in R, using genoplot R.

**Maximum likelihood core genome phylogeny of the *T. cruzi* strains/isolates and Principal Component Analysis (PCA) of *T. cruzi* whole genome variability**

To estimate the phylogeny of 36 *T. cruzi* strains and isolates, each read library was mapped to the same reference genome, *T. cruzi* CL Brener Esmeraldo-like version 46, using BWA-MEM v. 0.7.12 [(5)](https://paperpile.com/c/gErfVg/70D4Z). The mapped reads were filtered by mapping quality 30 using SAMtools v1.1 [(6)](https://paperpile.com/c/gErfVg/UEkgi). Duplicated reads were marked using Picard v1.119 (http://broadinstitute.github.io/picard/). RealignerTargetCreator (GATK v3.3) was used to re-align reads and SNP calls were performed with HaplotypeCaller (GATK v3.3), with minimal confidence threshold for calling of 30 (phred scale) and minimum threshold confidence for emission of 10 (phred scale). GATK SelectVariants was used to report only SNPs, and select SNP positions with read depth of at least 10 and phred quality higher than 30. A consensus nuclear genomic sequence was generated for each sample using GATK v3.3 FastaAlternateReferenceMaker [(8)](https://paperpile.com/c/gErfVg/hLG9N). Next, Pilon v. 1.22 [(9)](https://paperpile.com/c/gErfVg/M5Osl) was used to correct the consensus genomic sequence, using the flags --changes --diploid and --fix snps. To estimate the *T. cruzi* maximum likelihood phylogeny, the sequences of the 1,563 single copy genes described in Reis-Cunha 2015 [(3)](https://paperpile.com/c/gErfVg/cBql2) were extracted from the consensus nuclear sequences with BEDTools.v2.23 getfasta, using the coordinates in the General Feature Format (GFF) file, and concatenated in a single supermatrix sequence of 2,355,325 nucleotides. The jModelTest v2.1.7 program was used to estimate the best fitting nucleotide substitution model for the maximum likelihood phylogeny, using AIC, and BIC, which both suggested the GTR model, with 0.91 invariable sites and a value of alpha of 0.64 for the gamma distribution. The phylogenetic analysis was performed with PhyML v3.1 [(10)](https://paperpile.com/c/gErfVg/RMuG3), using the parameters estimated by jModelTest [(11)](https://paperpile.com/c/gErfVg/5lDBy) and 1,000 bootstrap replicates. The tree visualization was generated with ITOL [(12)](https://paperpile.com/c/gErfVg/EXVf). The Principal Component Analysis (PCA) of the whole genome variability of these strains was performed with the R “adegenet” package. The whole-genome differential SNPs among the 36 strains were obtained with the “DNAbin2genind” and “tab” functions, while the PCA evaluation was performed with the “dudi.pca” function. The PCA plot image was generated with R, using ggplot2.

**K-mer clustering parametrization**

This selection was based on four parameters: 1- elapsed clusterization time; 2- number of generated clusters; 3- number of k-mers that clustered with k-mers from other Multiple Expectation Maximizations for Motif Elicitation MEMEs (false positives) and 4- number of “singlets”, represented by k-mers without clusters. To select k-mers specific to each MEME, all generated MASP k-mers were used as a query in a BLASTx [(13)](https://paperpile.com/c/gErfVg/OPx0), with exact matches, using as subject the MASP MEMEs. Next, all k-mers that had a match with any of the MASP MEMEs were recovered, and clustered with UCLUST using the --optimal and --nofastalign options (every seed will be aligned to the query and assigned to the highest identity threshold); --rev (align in the plus and minus strand); --nucleo, (input sequence uses a nucleotide alphabet) and a cutoff of global identity varying from 0.75 to 0.95, by increases of 0.05. Based on these analyses, the global identity of 0.75 was selected for downstream analysis.

1. To select the optimal UCLUSTv1.2.22 parameters, the correspondence of MASP k-mers to the MASP MEMEs (see the material and methods section) was assessed using similarity cutoffs varying from 75% to 95%. A UCLUST clustering cutoff of 75% identity was selected, as only 26 (0.0347%) k-mers were grouped in clusters (motifs) with k-mers from other MEMEs, and only 9 (0.0012%) k-mers were singlets (did not cluster with any other k-mer). This resulted in a total of 0.047% errors, when compared with 0.060%; 0.144%, 0.391% and 11.19% errors, respectively, to the 80%, 85%, 90% and 95% cutoffs (S6 Fig).

**Comparison between multigene families’ expansions among isolates and their phylogeny**

Tanglegrams were generated to evaluate the correspondence between the multigene family’s cluster copy number variation (CNV), variability and the core-genome phylogeny based on single copy genes, as well as to compare each multigene family CNV and variability among each other (S4 Fig). There was a high correspondence between the multigene families dendrograms and core-genome phylogeny (S4 Fig A-C). Most of the incongruences occurred in small branches within DTUs or in the Tc231 (TcIII), CanIII (TcIV) and Tc9280 (TcV) strains. The Tc231 strain clustered closer to TcI strains when analyzing the core-genome phylogeny (S4 Fig), as well as MASPs (S4 Fig B), but clustered closer to the hybrid strains in TcMUC and TS (S4 Fig A and C). This could represent a higher correspondence between clusters from the TcMUC and TS in the hybrid DTUs and the TcIII parental strain. When the multigene family’s cluster dendrograms were compared with each other, there was a greater correspondence between MASP and TcMUC (S4 Fig D) than between MASP and TS (S4 Fig E) and TcMUC and TS (S4 Fig F). The multigene families dendrograms were supported with high bootstrap and AU support, where all branching’s had equal or above 80% bootstrap support, with exception of some internal TcI branches and in the CL Brener and SRR3676277 branch for the MASP multigene family dendrogram (S4 Fig B).

**Comparison of the most conserved cluster and the sum of coverages of all motifs**

We compared two metrics to assess the overall copy number of each family. 1-The sum of coverages of all motifs from a family; 2-The coverage of the most representative motif. To select the most representative cluster for each family, first clusters were filtered by a coefficient of variation (CoV - standard deviation/mean) lower than 1, to remove repetitive expansions. Then, for each family, a consensus sequence of clusters with the top 5 highest mean coverage across all isolates was generated using seqinR (https://cran.r-project.org/web/packages/seqinr/index.html) (S7 Fig A). A fasta of the consensus sequence where positions were conserved in at least 50% of the kmers in the cluster and a logo representation (ggseqlogo) was generated (https://cran.r-project.org/web/packages/ggseqlogo/index.html). This consensus sequence was used in a BLAST analysis [(13)](https://paperpile.com/c/gErfVg/OPx0) optimized for short queries (-dust no -task "blastn-short" -evalue 10000 -word_size 7 -max_target_seqs 10000) against all the coding sequences from the three CL Brener haplotypes and DM28 (TriTrypDB v.46), where only matches with coverage and identity higher than 70% were kept. The representative clusters were present in 75-92% of the full genes and 55-81% of genes annotated as pseudogenes (S7 Fig B), being localized in the 5’ region of the genes (S7 Fig C, D and E). This shows that even though the selected motifs are present in the majority of the genes from each family, no single motif alone is able to account for the whole variability of these massive gene families. The lower representation of the motifs in the pseudogenes may be due to pseudogenes’ potential higher evolutionary rate, or to misassembles in the reference genomes. Next, we estimated the correlation between the motif copy number and the sum of coverages for all motifs in the multigene families MASP (r=0.92, p-value =7.841 x10-^12^), TcMUC (r=0.95, p-value = 3.561x10^-14^) and TS (r= 0.76, p-value = 3.238 x 10^-6^) (S7 Fig F). Finally, to assess if the gene size of each multigene family’ varies among the evaluated DTUs, the size of the genes was estimated by subtracting the final and initial coordinates in the GFF file (TriTrypDB v 46) for each family MASP, TcMUC and TS in each reference (S7 Fig G). The strong correlation, combined with the facts that the most representative motifs were not present in all genes (specially pseudogenes) and that the distribution of sizes of the genes is similar among DTUs, led us to select the sum of coverages as a metric to assess multigene families copy number in each isolate.

**Cluster copy number and aneuploidies correlation**

To evaluate if the cluster's variation among *T. cruzi* strains is mainly a consequence of differential aneuploidy patterns, the cluster copy number was compared with the expected copy number of multigene families based on chromosome duplications. Initially, the Chromosomal Copy Number Variations (CCNV) in all *T. cruzi* strains was estimated using the SCoPE methodology [(3)](https://paperpile.com/c/gErfVg/cBql2), with the CL Brener 41 chromosomes as reference. Next, the number of nucleotides encoding multigene families (NNEMF) in each chromosome for both CL Brener haplotypes (Esmeraldo-like and Non-Esmeraldo) was estimated using GFF gene coordinates, obtained from TriTrypDB version 46 [(4)](https://paperpile.com/c/gErfVg/d16MF). Then, to estimate the “expected” copy number of each multigene family corrected by chromosome copy number, the NNEMF was multiplied by the chromosome estimated somy. Spearman correlation of this estimate and cluster copy numbers was performed in R. There was a low to moderate correlation between ploidy and cluster CNV for the multigene families TcMUC (rho = 0.385, P = 0.020), MASP (rho = 0.433, P = 0.008) and TS (rho = 0.504, P = 0.001).

***In silico* selection of conserved and potentially immunogenic k-mers**

The selection of conserved and potentially immunogenic k-mers derived from *T. cruzi* multigene families was performed *in silico*, using five steps:

**1- High copy number in all *T. cruzi* evaluated strains:** the distribution and the quantiles of copy-number of all clusters from the three multigene families were generated in R. All k-mers from clusters with a coverage equal or higher than the 75% quantile, excluding spots with “0” counts, were recovered. This cutoff value was 16.25 to MASP, 14.42 to TcMUC and 26.9 to TS.

**2- Exact k-mer presence in all *T. cruzi* evaluated strains**: as the clusters are composed by k-mers with high similarity, but with punctual nucleotide differences, only k-mers with identical sequences in all 36 evaluated *T. cruzi* strains were selected.

**3- K-mer encoded by complete genes**: as *T. cruzi* multigene families present a high number of pseudogenes, only k-mers that contained an exact BLASTn match (identity 100% along the entire sequence) with at least one CL Brener’s non-pseudogenes were recovered, using bash scripts. The coding sequence of each k-mer was determined based on the reading frame of its best-matching non-pseudogene and translated using Perl scripts with the Bio::SeqIO library. As all k-mers had 30 nucleotides, the generated peptides had between 9 and 10 amino acids, depending on the reading frame.

**4- Rank the k-mer-generated peptide sequences based on B-cell epitope and disorder predictions**: the k-mer-derived peptides were ranked based on their “immunogenicity score”, estimated by a combination of scores from BepiPred B-cell epitope v 1.0 [(14)](https://paperpile.com/c/gErfVg/ACiMA) and IUPred disordered sequence regions predictions [(15)](https://paperpile.com/c/gErfVg/jODR6). The top-scoring 600 peptides from each family were recovered for the following analysis.

**5- Filtering identical and highly similar peptides**: different k-mer sequences could generate the same or highly similar peptides. Hence, to reduce redundancy, when two or more peptides shared more than 8 amino acids, only the one with the highest epitope prediction score was maintained.

**Sera libraries**

Each experimental group contained six C57BL/6 mice six to eight weeks old, which were intraperitonially infected with trypomastigotes from the aforementioned strains. The DTU of *T. cruzi* parasites were previously confirmed by genotyping PCR, using the markers COII [(16)](https://paperpile.com/c/gErfVg/qMPgO), rDNA 24Sα [(17)](https://paperpile.com/c/gErfVg/PjEd7) and Mini-exon SL-IRac [(18)](https://paperpile.com/c/gErfVg/bIs3G). *Mycoplasma* contamination in tissue-culture cell-derived trypomastigotes was evaluated by PCR, using primers described by Timenetsky 2006 [(19)](https://paperpile.com/c/gErfVg/vUENo). The parasitemia during the acute phase was evaluated in intervals of five days, from the day of infection until day 30 post-infection, by collecting 5µL of blood from the mice’s tail and counting the number of parasites in 50 fields in an optical microscope. The reactivity of the sera to *T. cruzi* during the acute phase was evaluated at the same time points by ELISA, as described in [(20)](https://paperpile.com/c/gErfVg/fQVyz), using as antigen *T. cruzi* CL Brener epimastigote crude extracts and horseradish peroxidase-conjugated anti-mouse IgM secondary antibody (Sigma). To obtain anti- *T. cruzi* sera from chronic infections, the mice parasitemia was initially confirmed 5-10 days post-infection. Then, the absence of detectable parasitemia in optical microscopy three months after infection was assumed as a confirmation of the chronical stage of the infection.

**Selection of the best pots-infection interval to collect the mice sera**

As the parasitemia peak dates is highly divergent among *T. cruzi* strains from different DTUs, to select the most reactive time-point in the acute phase for each DTU, the reactivity of the mice sera pools was previously evaluated by ELISA, using CL Brener epimastigote crude extract as antigen. This leaded to the selection of the sera collected in the 15-days post-infection for Y (TcII) and CL Brener (TcVI) and 25-days post-infection for Colombiana (TcI), as the highest reactive sera.

**Spot synthesis and immunoblotting**

The peptides were synthesized on specific spots in a derivatized cellulose membrane, starting with the C-terminal of the last amino acid. The Fmoc group was removed with 4-methylpiperidine 25% in dimethylformamide (DMF). The amino acids were activated using DIC (diisopropylcarbodiimide) Oxyma Pure at 1.1 M and deposited in the membrane in two coupling cycles without a new deprotection step between them. The free amine residues were acetylated with acetic anhydride in 20% DMF. The newly coupled amino acid Fmoc protector group was removed with 25% 4-methylpiperidine in DMF. These steps were repeated until all the amino acids were added. At the end of the synthesis, the membrane was submerged during one hour in a cleavage solution composed by 95% (v/v) trifluoracetic acid (TFA) associated with 2.5% (v/v) water and 2,5% (v/v) triisopropylsilane (TIPS) to remove the protective groups from the amino acids side chains. After, the membrane was washed 4 times with dichloromethane (DCM), 4 times with DMF and 2 times with ethanol. The membrane was dried, and the spots checked on UV-light.

For the immunoblot experiments, the membrane was blocked for 16 hours with 5% BSA and 4% sucrose in PBS, washed 3 times for 10 minutes with 0.1% Tween-20 in PBS and incubated with one of the aforementioned pools of sera diluted 1:500 in PBS Tween 0.1% for 2 hours. Subsequently, the membrane was washed as described above, incubated with horseradish peroxidase-conjugated anti-mouse IgG or anti-mouse IgM or anti-human IgG (Sigma-Aldrich), respectively, for the experiments using samples from mice in the acute, chronic stages or human patients in the chronic stage of the disease; diluted 1:10,000 in PBS Tween 0.1% for 1 hour. The membrane was washed and visualized by chemiluminescence using Luminata Forte Western HRP substrate (Merck), with exposures of 1 or 10 minutes for acute or chronic phase sera, respectively, on an ImageQuant LAS 4000 digital imaging system (GE Healthcare). After data acquisition, the membrane was regenerated for use with another pool of sera. The regeneration was performed by washing the membrane 3 times with DMF for 10 minutes, followed by incubation for 16 hours with an 8 M urea, 1% SDS solution. The membrane was then washed twice for 30 minutes in the 8 M urea, 1% SDS solution, washed once with deionized water for 2 minutes, and then washed 3 times in 55% ethanol and 10% acetic acid for 10 minutes. Lastly, the membrane was washed for 2 minutes with deionized water.

**Densitometric evaluation of the immunoblotting assays**

The densitometric value of each spot was calculated using ImageJ software and Protein Array Analyzer plug-in (<http://image.bio.methods.free.fr/ImageJ/?Protein-Array-Analyzer-for-ImageJ.html>). Densitometric values were normalized and used to compare between families and host stages. To remove background noise reactivity, the mean densitometric value of the four lower-reacting spots from each experiment was subtracted from the densitometric value of each spot from the same membrane. For the pools of sera from mice infected with *T. cruzi* strains, a minimum reactivity cutoff of “15,833.20” for the IgM and “4,508.46” for the IgG membranes was established, based on the mean plus three times the standard deviation of the reactivity of all spots in the membrane evaluated with the uninfected mice sera. For the pools of sera from human patients, a minimum cutoff reactivity of “4,932.40” was established, based on the mean plus three standard deviations from the non-Chagasic human sera. To compare the reactivity of the mice sera to the three multigene families in the acute and chronic stages, the reactivity of each spot of each immunoblotting experiment for each multigene family was normalized, by dividing its densitometric value by the mean densitometric value of the family, and used to generate dendrograms, with the R Pvclust package (https://cran.r-project.org/web/packages/pvclust/index.html), based on Manhattan distance, average clustering method and 1,000 bootstrap replicates. The correlation of each pair of membrane reactivity was performed with the Spearman correlation, in R.

**References:**

1. [Andrews S, Others. 2010. FastQC: a quality control tool for high throughput sequence data. Babraham Bioinformatics, Babraham Institute, Cambridge, United Kingdom.](http://paperpile.com/b/gErfVg/wnlnr)

2. [Bolger AM, Lohse M, Usadel B. 2014. Trimmomatic: a flexible trimmer for Illumina sequence data. Bioinformatics 30:2114–2120.](http://paperpile.com/b/gErfVg/9HaAH)

3. [Reis-Cunha JL, Rodrigues-Luiz GF, Valdivia HO, Baptista RP, Mendes TAO, de Morais GL, Guedes R, Macedo AM, Bern C, Gilman RH, Lopez CT, Andersson B, Vasconcelos AT, Bartholomeu DC. 2015. Chromosomal copy number variation reveals differential levels of genomic plasticity in distinct Trypanosoma cruzi strains. BMC Genomics 16:499.](http://paperpile.com/b/gErfVg/cBql2)

4. [Aslett M, Aurrecoechea C, Berriman M, Brestelli J, Brunk BP, Carrington M, Depledge DP, Fischer S, Gajria B, Gao X, Gardner MJ, Gingle A, Grant G, Harb OS, Heiges M, Hertz-Fowler C, Houston R, Innamorato F, Iodice J, Kissinger JC, Kraemer E, Li W, Logan FJ, Miller JA, Mitra S, Myler PJ, Nayak V, Pennington C, Phan I, Pinney DF, Ramasamy G, Rogers MB, Roos DS, Ross C, Sivam D, Smith DF, Srinivasamoorthy G, Stoeckert CJ Jr, Subramanian S, Thibodeau R, Tivey A, Treatman C, Velarde G, Wang H. 2010. TriTrypDB: a functional genomic resource for the Trypanosomatidae. Nucleic Acids Res 38:D457–62.](http://paperpile.com/b/gErfVg/d16MF)

5. [Li H. 2013. Aligning sequence reads, clone sequences and assembly contigs with BWA-MEM. arXiv [q-bioGN].](http://paperpile.com/b/gErfVg/70D4Z)

6. [Li H, Handsaker B, Wysoker A, Fennell T, Ruan J, Homer N, Marth G, Abecasis G, Durbin R, 1000 Genome Project Data Processing Subgroup. 2009. The Sequence Alignment/Map format and SAMtools. Bioinformatics 25:2078–2079.](http://paperpile.com/b/gErfVg/UEkgi)

7. [Quinlan AR, Hall IM. 2010. BEDTools: a flexible suite of utilities for comparing genomic features. Bioinformatics 26:841–842.](http://paperpile.com/b/gErfVg/bQoRX)

8. [McKenna A, Hanna M, Banks E, Sivachenko A, Cibulskis K, Kernytsky A, Garimella K, Altshuler D, Gabriel S, Daly M, DePristo MA. 2010. The Genome Analysis Toolkit: a MapReduce framework for analyzing next-generation DNA sequencing data. Genome Res 20:1297–1303.](http://paperpile.com/b/gErfVg/hLG9N)

9. [Walker BJ, Abeel T, Shea T, Priest M, Abouelliel A, Sakthikumar S, Cuomo CA, Zeng Q, Wortman J, Young SK, Earl AM. 2014. Pilon: an integrated tool for comprehensive microbial variant detection and genome assembly improvement. PLoS One 9:e112963.](http://paperpile.com/b/gErfVg/M5Osl)

10. [Guindon S, Delsuc F, Dufayard J-F, Gascuel O. 2009. Estimating maximum likelihood phylogenies with PhyML. Methods Mol Biol 537:113–137.](http://paperpile.com/b/gErfVg/RMuG3)

11. [Posada D. 2008. jModelTest: phylogenetic model averaging. Mol Biol Evol 25:1253–1256.](http://paperpile.com/b/gErfVg/5lDBy)

12. [Letunic I, Bork P. 2021. Interactive Tree Of Life (iTOL) v5: an online tool for phylogenetic tree display and annotation. Nucleic Acids Res 49:W293–W296.](http://paperpile.com/b/gErfVg/EXVf)

13. [Altschul SF, Gish W, Miller W, Myers EW, Lipman DJ. 1990. Basic local alignment search tool. J Mol Biol 215:403–410.](http://paperpile.com/b/gErfVg/OPx0)

14. [Larsen JEP, Lund O, Nielsen M. 2006. Improved method for predicting linear B-cell epitopes. Immunome Res 2:2.](http://paperpile.com/b/gErfVg/ACiMA)

15. [Dosztányi Z, Csizmok V, Tompa P, Simon I. 2005. IUPred: web server for the prediction of intrinsically unstructured regions of proteins based on estimated energy content. Bioinformatics 21:3433–3434.](http://paperpile.com/b/gErfVg/jODR6)

16. [de Freitas JM, Augusto-Pinto L, Pimenta JR, Bastos-Rodrigues L, Gonçalves VF, Teixeira SMR, Chiari E, Junqueira ACV, Fernandes O, Macedo AM, Machado CR, Pena SDJ. 2006. Ancestral genomes, sex, and the population structure of Trypanosoma cruzi. PLoS Pathog 2:e24.](http://paperpile.com/b/gErfVg/qMPgO)

17. [Souto RP, Fernandes O, Macedo AM, Campbell DA, Zingales B. 1996. DNA markers define two major phylogenetic lineages of Trypanosoma cruzi. Mol Biochem Parasitol 83:141–152.](http://paperpile.com/b/gErfVg/PjEd7)

18. [Burgos JM, Altcheh J, Bisio M, Duffy T, Valadares HMS, Seidenstein ME, Piccinali R, Freitas JM, Levin MJ, Macchi L, Macedo AM, Freilij H, Schijman AG. 2007. Direct molecular profiling of minicircle signatures and lineages of Trypanosoma cruzi bloodstream populations causing congenital Chagas disease. Int J Parasitol 37:1319–1327.](http://paperpile.com/b/gErfVg/bIs3G)

19. [Timenetsky J, Santos LM, Buzinhani M, Mettifogo E. 2006. Detection of multiple mycoplasma infection in cell cultures by PCR. Braz J Med Biol Res 39:907–914.](http://paperpile.com/b/gErfVg/vUENo)

20. [Reis-Cunha JL, Mendes TA de O, de Almeida Lourdes R, Ribeiro DR dos S, Machado-de-Avila RA, de Oliveira Tavares M, Lemos DS, Câmara ACJ, Olórtegui CC, de Lana M, da Cunha Galvão LM, Fujiwara RT, Bartholomeu DC. 2014. Genome-wide screening and identification of new Trypanosoma cruzi antigens with potential application for chronic Chagas disease diagnosis. PLoS One 9:e106304.](http://paperpile.com/b/gErfVg/fQVyz)
