# Supplementary figures and images for: Accessing the Variability of Multicopy Genes in Complex Genomes using Unassembled Next-Generation Sequencing Reads: The Case of Trypanosoma cruzi Multigene Families
Source: mBio. 2022 Oct 20;13(6):e02319-22. doi: 10.1128/mbio.02319-22 (PMC9765020; doi:10.1128/mbio.02319-22)

**Cluster dendrogram with AU/BP values (%)**

Height

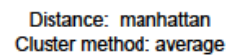

Supplement: Fig S3 [file mbio.02319-22-s0004.pdf]
