# Supplementary material for: Accessing the Variability of Multicopy Genes in Complex Genomes using Unassembled Next-Generation Sequencing Reads: The Case of Trypanosoma cruzi Multigene Families
Source: mBio. 2022 Oct 20;13(6):e02319-22. doi: 10.1128/mbio.02319-22 (PMC9765020; doi:10.1128/mbio.02319-22)

S4 Fig: Comparative tanglegrams of single copy core-genome phylogeny and multigene family's clusters CNV. This image corresponds to comparative tanglegrams of: A) Core-genome (CG) phylogeny and TcMUC clusters; B) CG phylogeny and MASP clusters; C) CG phylogeny and TS clusters; D) MASP and TcMUC clusters; E) MASP and TS clusters and F) TcMUC and TS clusters. The dendrograms were rooted in Tc9280 clade, and the branches were flipped (but not replaced) to adjust to the linking between nodes. The DTU of origin of each sample is represented by colors, where blue, red, pink, orange, purple, green correspond, respectively, to TcI, TcII, TcIII, TcIV, TcV, TcVI.

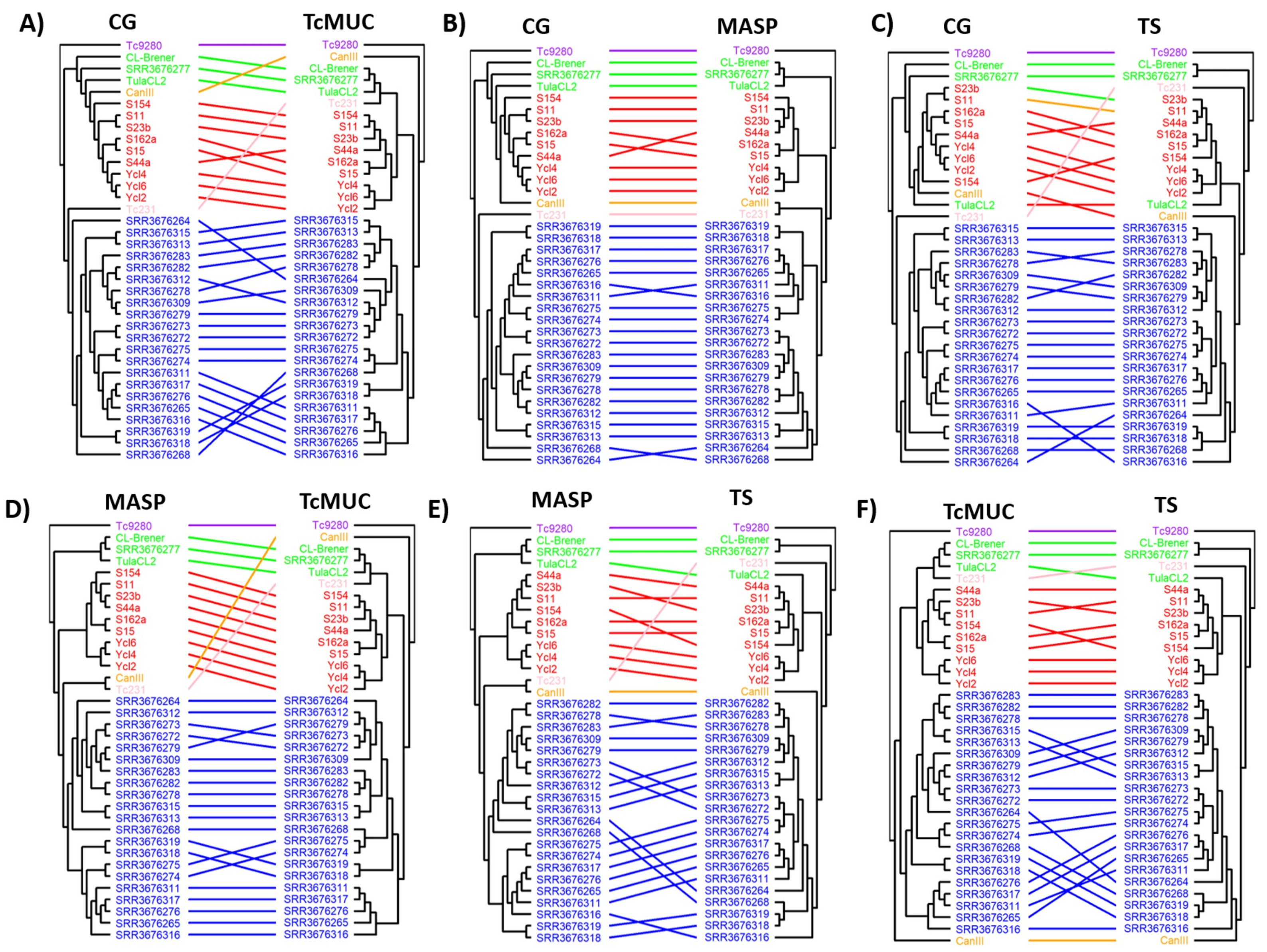

Supplement: Fig S4 [file mbio.02319-22-s0005.pdf]
