# Supplementary material for: Accessing the Variability of Multicopy Genes in Complex Genomes using Unassembled Next-Generation Sequencing Reads: The Case of Trypanosoma cruzi Multigene Families
Source: mBio. 2022 Oct 20;13(6):e02319-22. doi: 10.1128/mbio.02319-22 (PMC9765020; doi:10.1128/mbio.02319-22)
Supplement: Fig S2 [file mbio.02319-22-s0003.pdf]

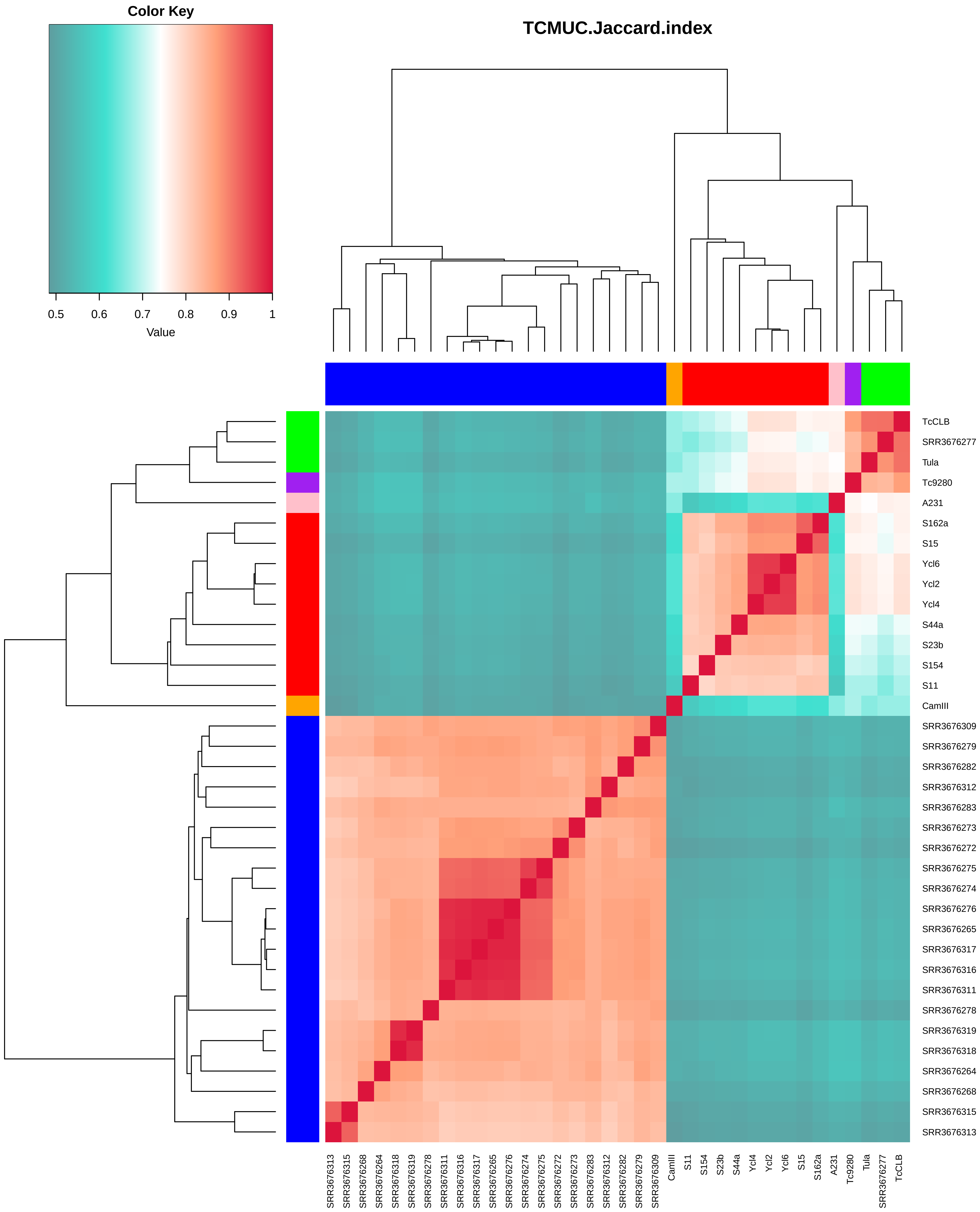
Color Key
0.5
0.6
0.7
0.8
0.9
1
Value
TCMUC.Jaccard.index
TcCLB
SRR3676277
Tula
Tc9280
A231
S162a
S15
Ycl6
Ycl2
Ycl4
S44a
S23b
S154
S11
CamIII
SRR3676309
SRR3676279
SRR3676282
SRR3676312
SRR3676283
SRR3676273
SRR3676272
SRR3676275
SRR3676274
SRR3676276
SRR3676265
SRR3676317
SRR3676316
SRR3676311
SRR3676278
SRR3676319
SRR3676318
SRR3676264
SRR3676268
SRR3676315
SRR3676313

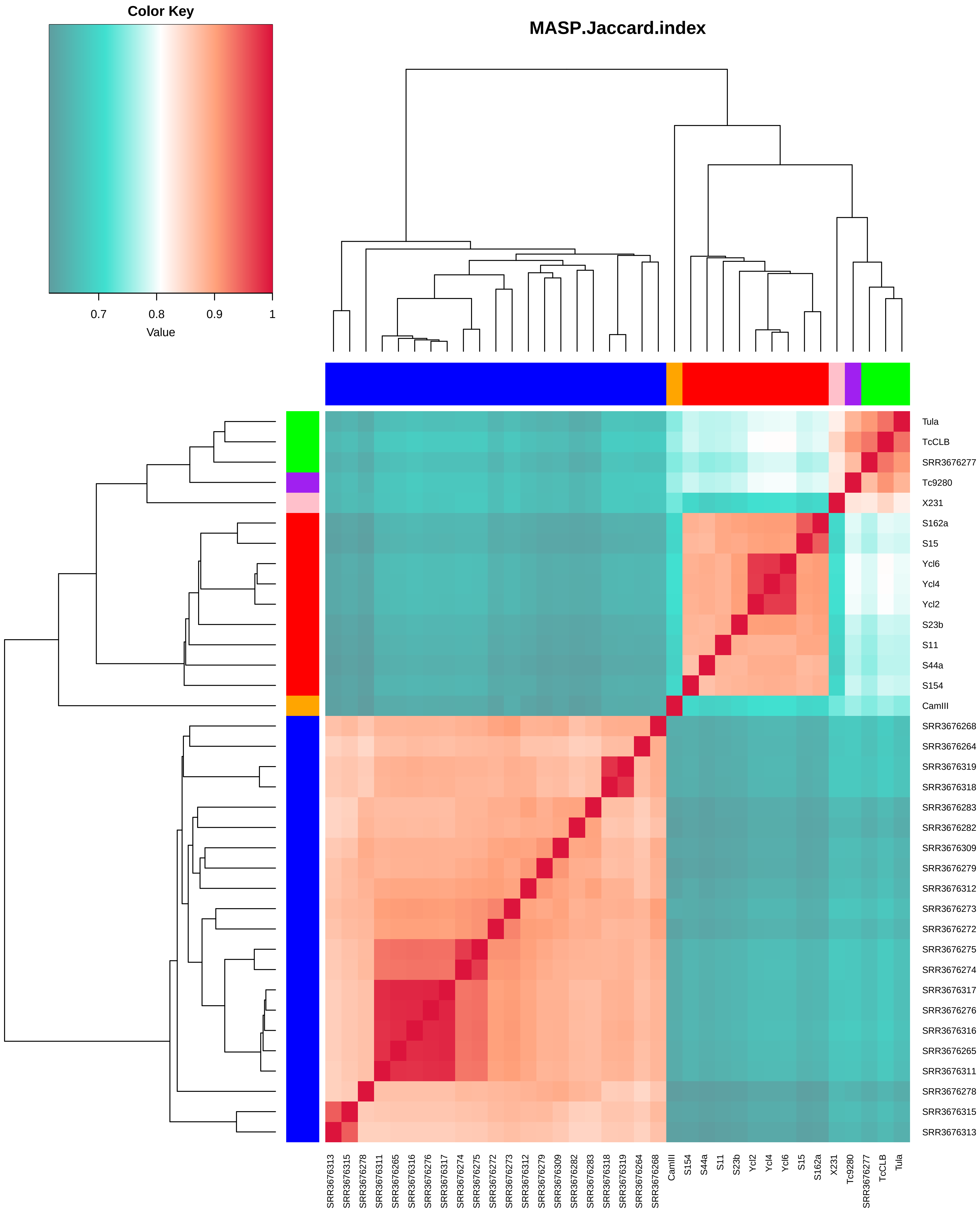

Color Key
0.7
0.8
0.9
1
Value
MASP.Jaccard.index
Tula
TcCLB
SRR3676277
Tc9280
X231
S162a
S15
Ycl6
Ycl4
Ycl2
S23b
S11
S44a
S154
CamIII
SRR3676268
SRR3676264
SRR3676319
SRR3676318
SRR3676283
SRR3676282
SRR3676309
SRR3676279
SRR3676312
SRR3676273
SRR3676272
SRR3676275
SRR3676274
SRR3676317
SRR3676276
SRR3676316
SRR3676265
SRR3676311
SRR3676278
SRR3676315
SRR3676313

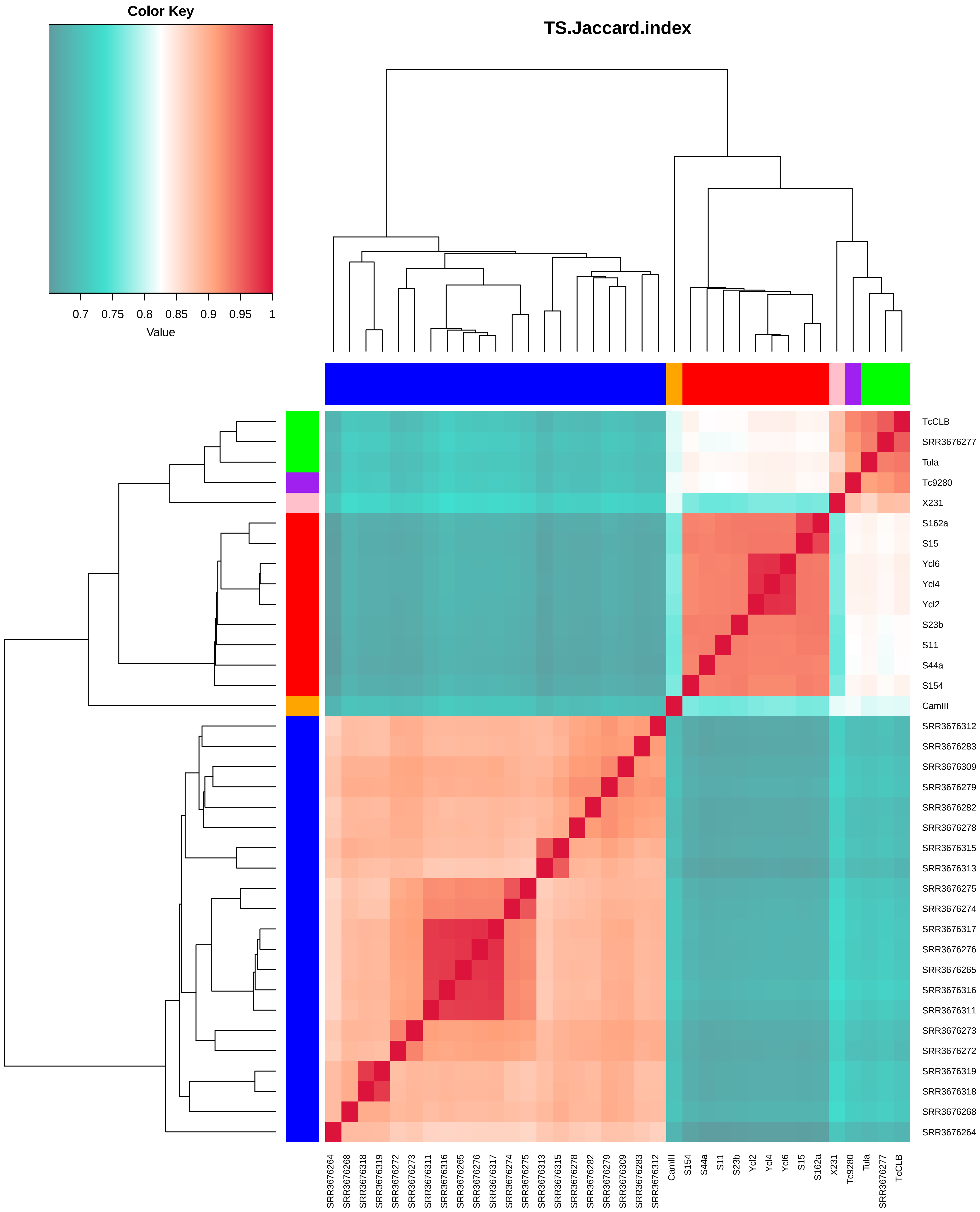
Color Key
0.7 0.75 0.8 0.85 0.9 0.95 1
Value
TS.Jaccard.index
TcCLB
SRR3676277
Tula
Tc9280
X231
S162a
S15
Ycl6
Ycl4
Ycl2
S23b
S11
S44a
S154
CamIII
SRR3676312
SRR3676283
SRR3676309
SRR3676279
SRR3676282
SRR3676278
SRR3676315
SRR3676313
SRR3676275
SRR3676274
SRR3676317
SRR3676276
SRR3676265
SRR3676316
SRR3676311
SRR3676273
SRR3676272
SRR3676319
SRR3676318
SRR3676268
SRR3676264

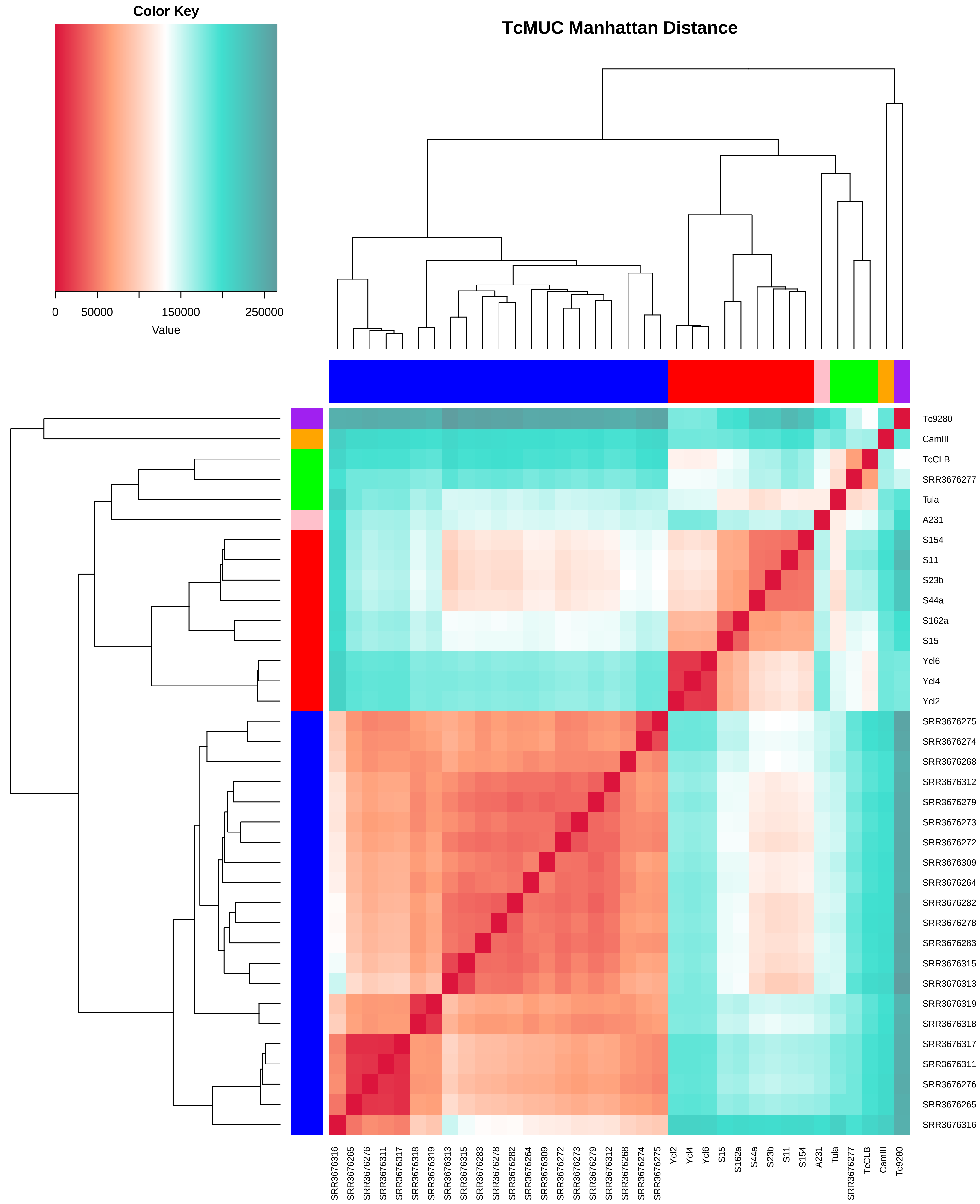

Color Key
0
50000
150000
250000
Value
TcMUC Manhattan Distance
Tc9280
CamIII
TcCLB
SRR3676277
Tula
A231
S154
S11
S23b
S44a
S162a
S15
Ycl6
Ycl4
Ycl2
SRR3676275
SRR3676274
SRR3676268
SRR3676312
SRR3676279
SRR3676273
SRR3676272
SRR3676309
SRR3676264
SRR3676282
SRR3676278
SRR3676283
SRR3676315
SRR3676313
SRR3676319
SRR3676318
SRR3676317
SRR3676311
SRR3676276
SRR3676265
SRR3676316

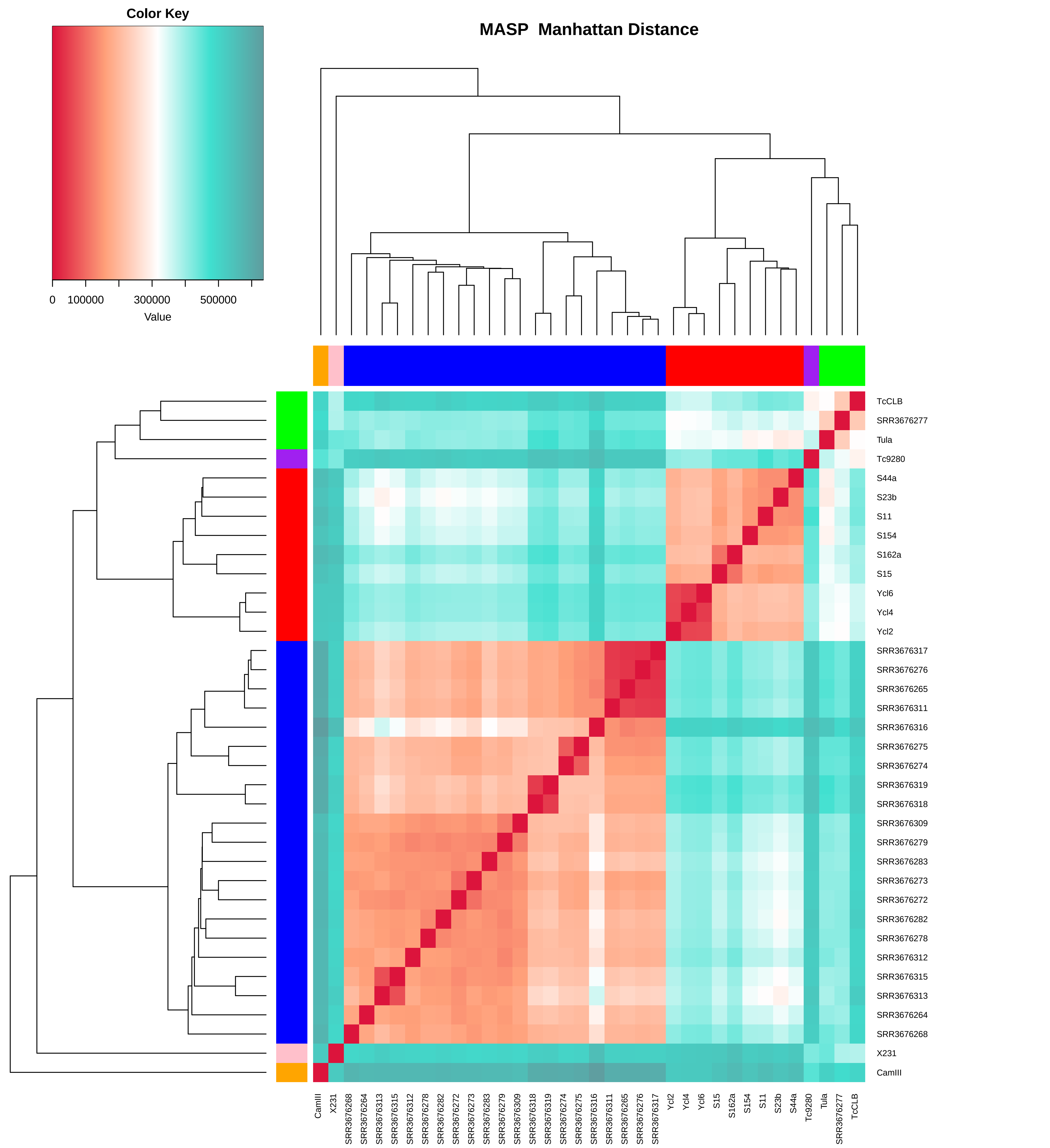
Color Key
0
100000
300000
500000
Value
MASP Manhattan Distance
TcCLB
SRR3676277
Tula
Tc9280
S44a
S23b
S11
S154
S162a
S15
Ycl6
Ycl4
Ycl2
SRR3676317
SRR3676276
SRR3676265
SRR3676311
SRR3676316
SRR3676275
SRR3676274
SRR3676319
SRR3676318
SRR3676309
SRR3676279
SRR3676283
SRR3676273
SRR3676272
SRR3676282
SRR3676278
SRR3676312
SRR3676315
SRR3676313
SRR3676264
SRR3676268
X231
CamIII

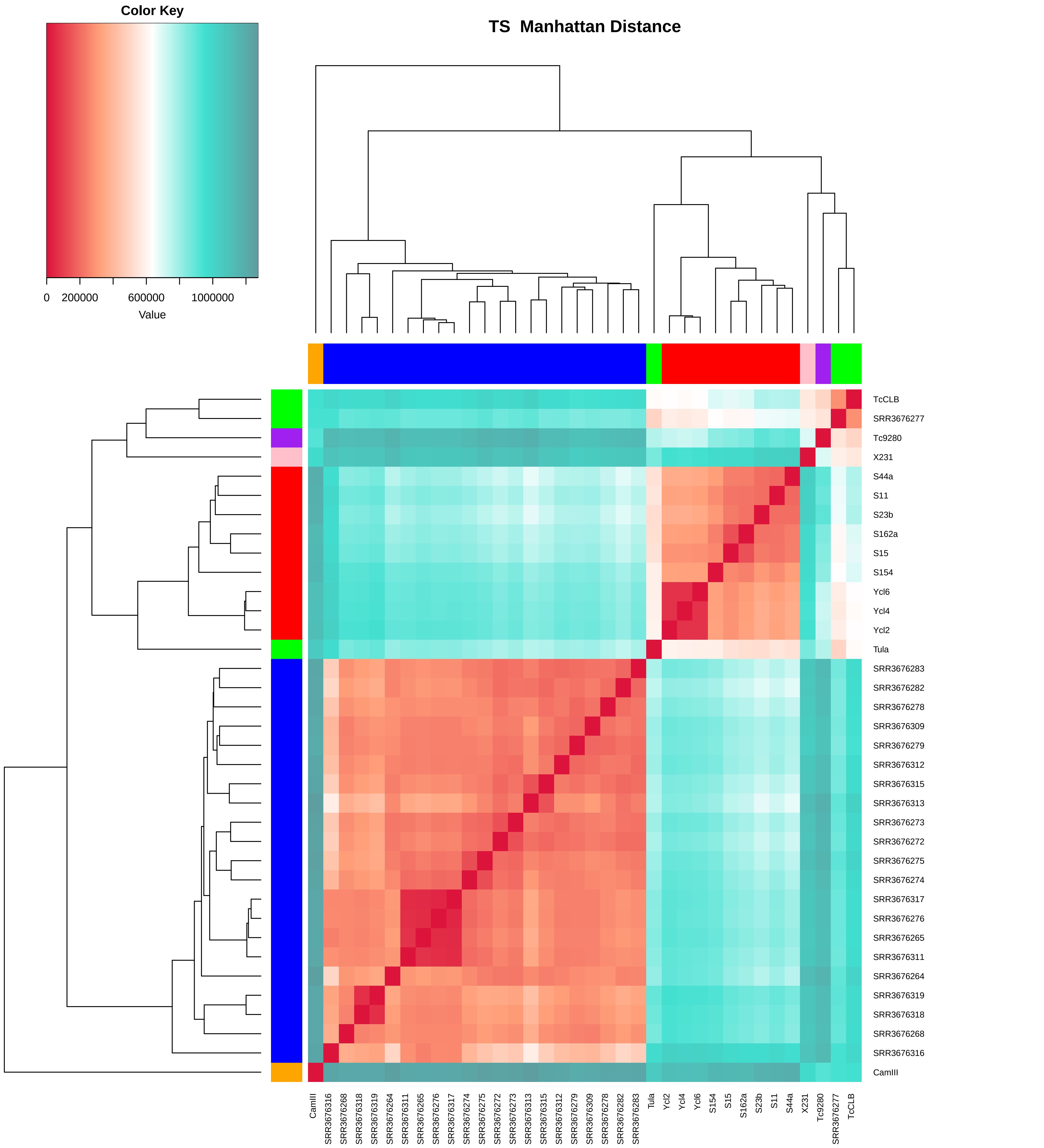
TS Manhattan Distance
Color Key
0
200000
600000
1000000
Value
TcCLB
SRR3676277
Tc9280
X231
S44a
S11
S23b
S162a
S15
S154
Ycl6
Ycl4
Ycl2
Tula
SRR3676283
SRR3676282
SRR3676278
SRR3676309
SRR3676279
SRR3676312
SRR3676315
SRR3676313
SRR3676273
SRR3676272
SRR3676275
SRR3676274
SRR3676317
SRR3676276
SRR3676265
SRR3676311
SRR3676264
SRR3676319
SRR3676318
SRR3676268
SRR3676316
CamIII
