# Supplementary material for: Accessing the Variability of Multicopy Genes in Complex Genomes using Unassembled Next-Generation Sequencing Reads: The Case of Trypanosoma cruzi Multigene Families
Source: mBio. 2022 Oct 20;13(6):e02319-22. doi: 10.1128/mbio.02319-22 (PMC9765020; doi:10.1128/mbio.02319-22)

S5 Fig: Venn diagram of the multigene family's reactive peptides to the sera of mice in the acute and chronic phases of *T. cruzi* infection. A total of 40 TcMUC, 113 MASP and 182 TS peptides were evaluated. The number in parenthesis below each DTU, ex. TcI (19), represents the number of peptides that were reactive with the sera of mice infected with TcI DTU, in the acute stage. "Acute": results for the sera of mice in the acute stage. "Chronic": result for the sera of mice in the chronic stage. "Acute and Chronic": peptides that had above cutoff values in the acute and chronic stages simultaneously. Percentage values correspond to the fraction of the reactive peptides that were observed in each quadrant.

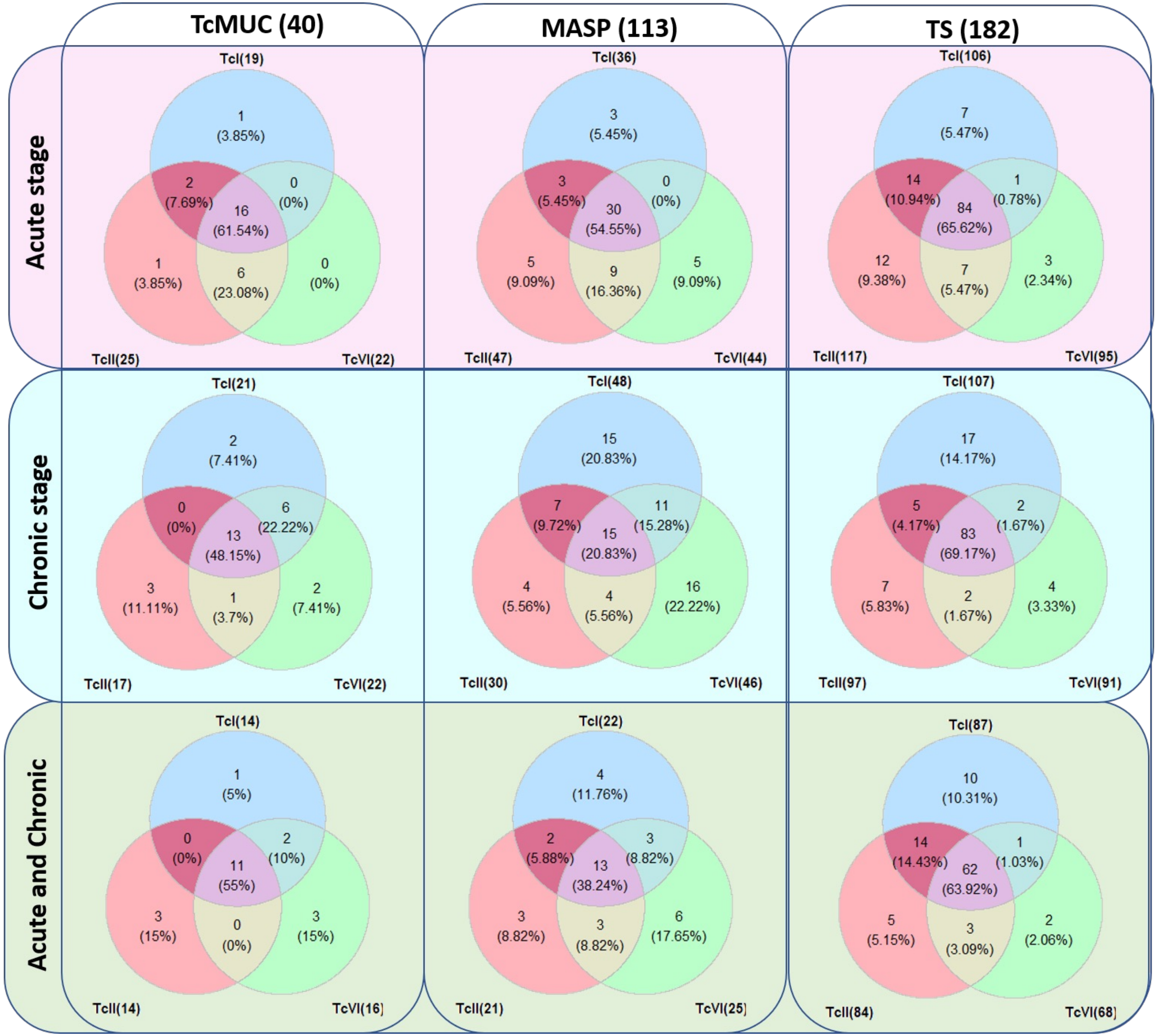

Supplement: Fig S5 [file mbio.02319-22-s0006.pdf]
