# Supplementary material for: Accessing the Variability of Multicopy Genes in Complex Genomes using Unassembled Next-Generation Sequencing Reads: The Case of Trypanosoma cruzi Multigene Families
Source: mBio. 2022 Oct 20;13(6):e02319-22. doi: 10.1128/mbio.02319-22 (PMC9765020; doi:10.1128/mbio.02319-22)

S6 Figure: Selection of the UCLUST clustering identity cutoff. To select the UCLUST clustering cutoff, the correspondence between k-mers which match the MASP MEMEs described in EL-Sayed 2005 and the generated clusters were evaluated. A) Several parameters were estimated, as the time spent in clustering, number of clusters generated, k-mers from different MEMEs that grouped in the same cluster, number of singlets (k-mers not assigned to any cluster), the number of cluster with less than 10 k-mers, mean and median k-mer/cluster ratios. The "Errors (%)" column corresponds to the sum of the values from the "k-mers clustered in incorrect clusters" and "Singlets", representing the percent of erroneous or non-assigned k-mers. B) Density plot of the distribution of the number of k-mers in clusters for the 0.75 to 0.90% cutoffs. The 0.95% was not represented as it collapsed the image, as most of its clusters presented a low k-mer count (median 2).

A)

	Time (seconds)	Number of clusters	K-mers clustered in incorrect motifs	Singlets	Errors (%)	Clusters with 10 or less Kmers	Mean K-mers/Cluster	Median K-mers/Cluster
Cutoff0.75	704	631	26 (0.034%)	9 (0.012%)	0.0468	97	118	61
Cutoff0.80	1071	1012	24 (0.032%)	21(0.028%)	0.0600	203	73	37
Cutoff0.85	2972	3116	7 (0.009%)	101(0.135%)	0.1444	1224	24	14
Cutoff0.90	5868	6160	5 (0.006%)	288(0.385%)	0.391	3658	12	9
Cutoff0.95	27286	31751	0 (0%)	8365(11.195%)	11.195	31742	2	2

B)

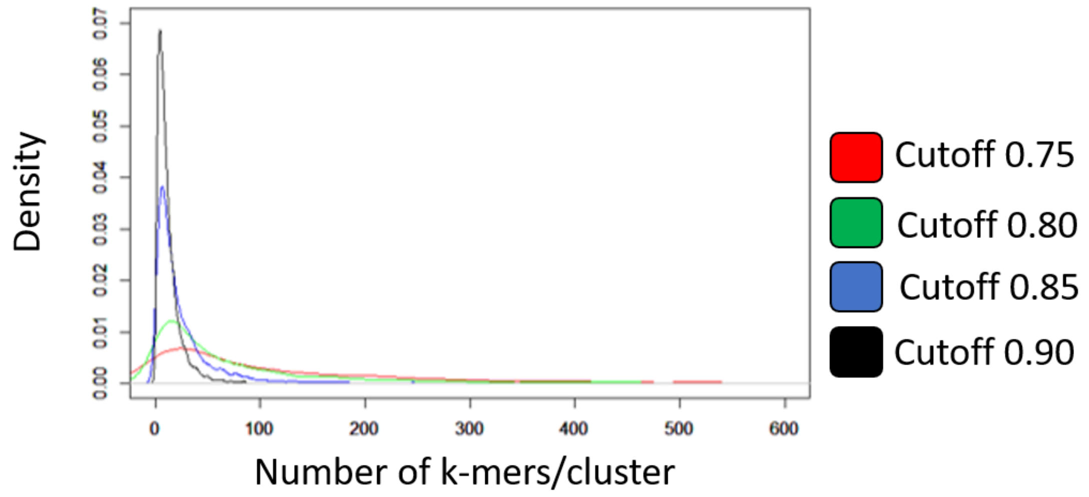

Supplement: Fig S6 [file mbio.02319-22-s0007.pdf]
