# Supplementary material for: Accessing the Variability of Multicopy Genes in Complex Genomes using Unassembled Next-Generation Sequencing Reads: The Case of Trypanosoma cruzi Multigene Families
Source: mBio. 2022 Oct 20;13(6):e02319-22. doi: 10.1128/mbio.02319-22 (PMC9765020; doi:10.1128/mbio.02319-22)

S7 Fig: Estimating the gene copy number of multigene families: A) Consensus sequence of all the k-mers in the most conserved cluster for each multigene family: MASP Cluster 25448; TcMUC Cluster 1488 and TS Cluster 3938. In the image, "M" represents a gap position. **B)** Number of BLAST matches in genes for each consensus of the conserved clusters. Freq = Number of genes with a match with the consensus sequence; Total genes: Total number of annotated genes of the family in the evaluated references; Rep = (Representativity): Proportion of genes with matches with the consensus of the cluster. **C)**, **D)** and **E)** correspond to the density of the distribution of the initial (I coord - red) and final (F coord - cyan) BLAST match coordinates, respectively, for the multigene families MASP, TcMUC and TS. This shows that the selected conserved motifs are localized in the 5' region of the genes, for the three families. **F)** Correlation between the copy number of the most relevant cluster (X axis) and the sum of coverages of all motifs (Y axis). **G)** Multigene family's gene size. CBEL = CL Brener Esmo like; CBNE = CL Brener Non-Esmeraldo-like; CBUC = CL Brener Unassigned contigs; DM28C = DM28 strain.

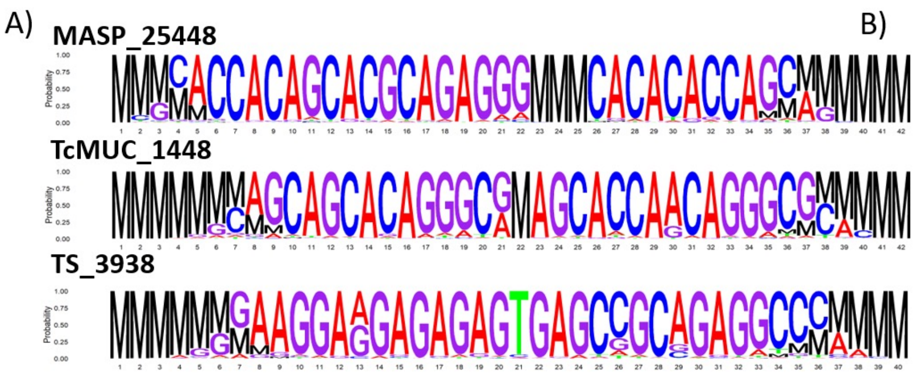

B)

**MASP-Cluster_25448**				
**Annotation**	**Freq**	**Total Genes**		**Rep**
MASP	1657	Complete	1797	0.92
Mucin-associated surface protein (MASP) (pseudogene)	315	Pseudogene	389	0.81
mucin TcMUCII	2			
hypothetical protein	1			
trans-sialidase	1			
TcMU-Cluster_1448				
Annotation	Freq	Total Genes		Rep
TcMUC	1066	Complete	1219	0.87
mucin TcMUC(pseudogene)	89	Pseudogene	200	0.45
MASP	19			
hypothetical protein	13			
MASP (pseudogene)	5			
Trypomastigote small surface antigen protein	3			
TS-Cluster_3938	Freq	Total Genes		Rep
Annotation	1082	Complete	1446	0.75
trans-sialidase (pseudogene)	394	Pseudogene	720	0.55
complement regulatory protein	4			
hypothetical protein	4			
neutral sphingomyelinase activation associated factor	3			
complement regulatory protein	1			

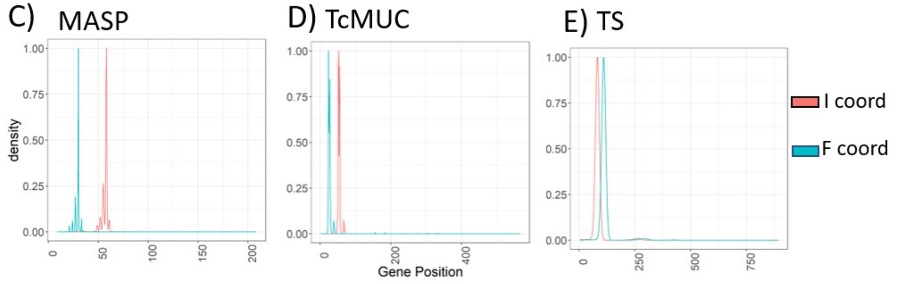

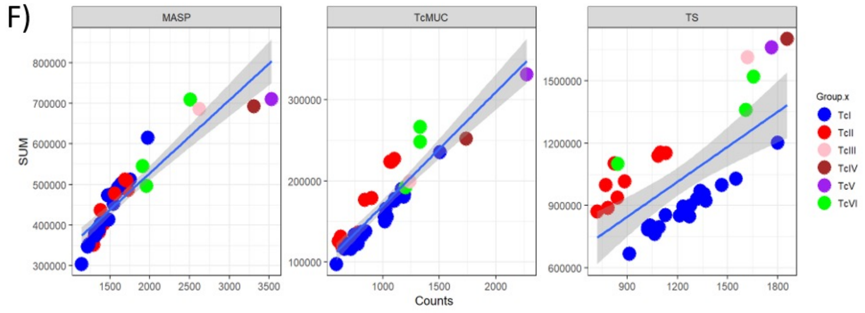

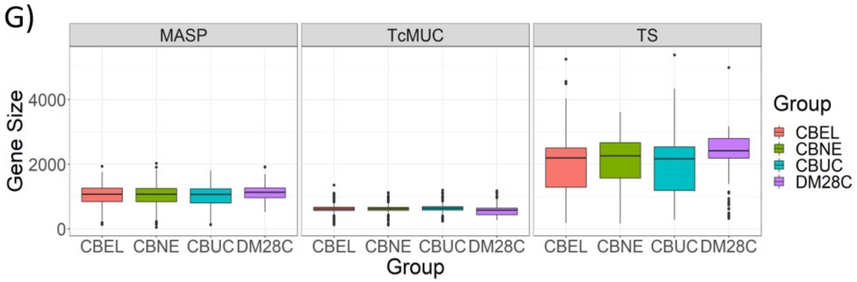

Supplement: Fig S7 [file mbio.02319-22-s0008.pdf]
